# Stereochemical Features of Glutathione-dependent Enzymes in the *Sphingobium* sp. Strain SYK-6 β-Aryl Etherase Pathway*[Fn FN2]

**DOI:** 10.1074/jbc.M113.536250

**Published:** 2014-02-07

**Authors:** Daniel L. Gall, Hoon Kim, Fachuang Lu, Timothy J. Donohue, Daniel R. Noguera, John Ralph

**Affiliations:** From the Departments of ‡ Civil and Environmental Engineering,; ¶ Biochemistry, and; ‖ Bacteriology and; the ** Environmental Chemistry and Technology Program, University of Wisconsin, Madison, Wisconsin 53706 and; the § United States Department of Energy Great Lakes Bioenergy Research Center, Wisconsin Energy Institute, University of Wisconsin, Madison, Wisconsin 53726

**Keywords:** Bacterial Metabolism, Enzyme Catalysis, Glutathione, Lignin Degradation, Thiol, beta-*S*-Thioetherase, beta-Aryl Etherase, Glutathione *S*-Transferase, Stereoselectivity, Stereospecificity

## Abstract

Glutathione-dependent enzymes play important protective, repair, or metabolic roles in cells. In particular, enzymes in the glutathione *S*-transferase (GST) superfamily function in stress responses, defense systems, or xenobiotic detoxification. Here, we identify novel features of bacterial GSTs that cleave β-aryl ether bonds typically found in plant lignin. Our data reveal several original features of the reaction cycle of these GSTs, including stereospecific substrate recognition and stereoselective formation of β-*S*-thioether linkages. Products of recombinant GSTs (LigE, LigP, and LigF) are β-*S*-glutathionyl-α-keto-thioethers that are degraded by a β-*S*-thioetherase (LigG). All three Lig GSTs produced the ketone product (β-*S*-glutathionyl-α-veratrylethanone) from an achiral side chain-truncated model substrate (β-guaiacyl-α-veratrylethanone). However, when β-etherase assays were conducted with a racemic model substrate, β-guaiacyl-α-veratrylglycerone, LigE- or LigP-catalyzed reactions yielded only one of two potential product (β-*S*-glutathionyl-α-veratrylglycerone) epimers, whereas the other diastereomer (differing in configuration at the β-position (*i.e.* its β-epimer)) was produced only in the LigF-catalyzed reaction. Thus, β-etherase catalysis causes stereochemical inversion of the chiral center, converting a β(*R*)-substrate to a β(*S*)-product (LigE and LigP), and a β(*S*)-substrate to a β(*R*)-product (LigF). Further, LigG catalyzed glutathione-dependent β-*S*-thioether cleavage with β-*S*-glutathionyl-α-veratrylethanone and with β(*R*)-configured β-*S*-glutathionyl-α-veratrylglycerone but exhibited no or significantly reduced β-*S*-thioether-cleaving activity with the β(*S*)-epimer, demonstrating that LigG is a stereospecific β-thioetherase. We therefore propose that multiple Lig enzymes are needed in this β-aryl etherase pathway in order to cleave the racemic β-ether linkages that are present in the backbone of the lignin polymer.

## Teaser

**Background:** A bacterial β-etherase pathway uses glutathione-dependent enzymes for catabolism of β-ether-linked substructures found in lignin.

**Results:** Racemic β-ether-linked substructures are stereoselectively converted to thioether-linked monoaromatic compounds by β-etherase pathway enzymes.

**Conclusion:** Multiple enzymes with complementary stereochemical features are needed to catabolize racemic lignin substructures.

**Significance:** Understanding the β-etherase pathway metabolism of lignin enhances our potential to use lignin for agricultural, industrial, and biotechnological purposes.

## Introduction

Glutathione (GSH) and GSH-dependent enzymes play crucial roles in cellular stress responses, protection against cell damage, or detoxification of xenobiotic compounds (1–4). In well studied members of the glutathione *S*-transferase (GST) family, the GSH thiol initiates a nucleophilic attack on the substrate during catalysis. Here we report new and novel aspects of GSTs that are implicated in lignin degradation.

Lignin is a major component of plant cell walls that is composed of polymerized aromatic subunits (5–7). This polymer provides both structural rigidity and protection from pathogens (8, 9). Lignin could be of economic value if efficient depolymerization strategies can be developed (10). β-*O*-4′-Aryl ether (henceforth termed β-ether) bonds comprise the majority of the linkages between monomer-derived aromatic units in lignin (11), so cleavage of these bonds could yield valuable products (12, 13). Here, we report on activities of GST family members that are proposed to cleave β-ether bonds typical of those found in the lignin backbone.

**FIGURE 1.**
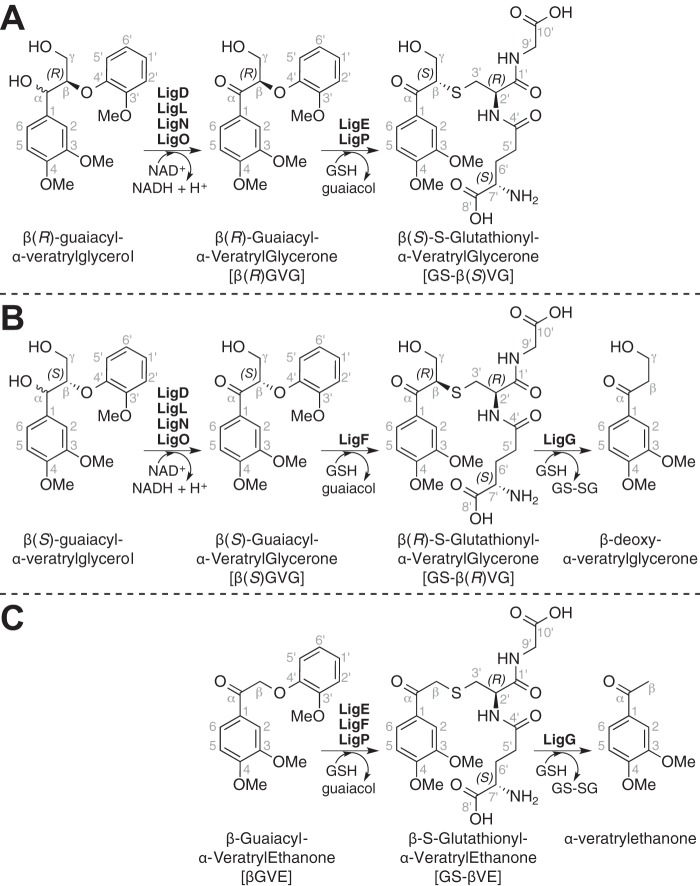
**The proposed β-etherase pathway in *Sphingobium* sp. strain SYK-6.** Panels (*A*) and (*B*) show pathways for metabolism of β(*R*)- and β(*S*)-configured β-aryl ether model compounds, with Lig dehydrogenases (LigD, LigL, LigN, and LigO) catalyzing oxidation of the benzylic alcohol in model substrates β(*R*)- and β(*S*)-guaiacyl-α-veratrylglycerol, producing β(*R*)GVG and β(*S*)GVG. In the presence of GSH, βGVG enantiomers are cleaved to guaiacol and one of two GS-conjugated β-epimers, GS-β(*S*)VG or GS-β(*R*)VG, by either LigE, LigP, or LigF. Subsequent GSH-dependent cleavage of GS-β(*R*)VG by LigG generates β-deoxy-α-veratrylglycerone and *GS-SG. C* shows the reaction products of Lig enzymes when the achiral model compound βGVE is used as a substrate. In this case, the product of β-etherase activity is GS-βVE, and the product of GS-βVE thioether cleavage by LigG is α-veratrylethanone.

The GST proteins that we are studying are part of a bacterial pathway (Fig. 1, *A* and *B*) that allows growth on complex aromatic substrates (14–20). Although many GST family members have been well studied, the properties of these enzymes are not well known. Plant lignin is synthesized via radical coupling of aromatic monomers, the hydroxy-cinnamyl alcohols or monolignols, mainly by their chemical condensation with the growing polymer. Because monolignols invariably couple at their β-positions, chiral centers are generated in each chain extension step. Each dimeric unit in the polymer contains two chiral centers, but the polymer is racemic (21, 22). The mechanisms by which the enzymes in this degradative Lig pathway process the racemic β-ether units found in lignin is an enduring knowledge gap.

In the best studied β-ether degradation pathway from the α-proteobacterium *Sphingobium* sp. strain SYK-6, the benzylic ketones proposed to be β-etherase substrates (14, 16, 17) are produced by oxidation of their corresponding benzylic alcohols (23). Based on the existence of multiple α-dehydrogenases that oxidize these benzylic alcohols, it is proposed that each enzyme separately oxidizes substrates with different stereochemical configurations at the α-position (19, 21, 24, 25). For example, oxidation of a model dimeric Lig substrate β-guaiacyl-α-veratrylglycerol yields two enantiomeric products, β(*R*)- and β(*S*)-enantiomers (Fig. 1, *A* and *B*) of β-guaiacyl-α-veratrylglycerone.

In the next step of this pathway, three GSH-dependent GST enzymes (LigE, LigP, and LigF) contribute to degradation of substrates containing β-aryl ether linkages like β-guaiacyl-α-veratrylglycerone (βGVG)^2^ (18, 26, 27). In *in vitro* assays with racemic substrates, cell extracts containing LigE and LigP separately degrade the β(*R*)GVG enantiomer while lacking detectable activity with the β(*S*)GVG substrate under identical assay conditions (Fig. 1*A*) (18). In contrast, LigF is active with the β(*S*)GVG enantiomer but has no detectable activity with β(*R*)GVG (Fig. 1*B*). The LigE, LigP, and LigF reaction products include guaiacol and a previously unidentified compound. The properties of the uncharacterized compound obtained using partially purified LigF were consistent with its being a GSH-conjugated aromatic monomer. However, from available data (18), the type and location of the bond that links GSH and the aromatic moiety are unknown, leaving open the chemical identity of the intermediates produced by these enzymes (Fig. 1, *A* and *B*, shows a putative GSH conjugate to veratrylglycerone, namely β-*S*-glutathionyl-α-veratrylglycerone, hereafter referred to as GS-βVG).

Evidence supporting the existence of a GSH conjugate in this pathway includes (*a*) cleavage of β(*S*)GVG and concomitant production of β-deoxy-α-veratrylglycerone (Fig. 1*B*) in coupled assays containing racemic βGVG, GSH, LigF, and LigG (a subsequent pathway enzyme believed to have β-thioetherase activity) and (*b*) the finding that a coupled assay containing LigE, LigG, GSH, and racemic βGVG resulted in β(*R*)GVG degradation but did not produce β-deoxy-α-veratrylglycerone (Fig. 1*A*) (18). Based on these facts and the hypothesis that LigF is a stereoselective GST, LigG is proposed to stereospecifically cleave a putative β-*S*-glutathionyl thioether in the presence of GSH, producing glutathione disulfide (*GS-SG*) and β-deoxy-α-veratrylglycerone (Fig. 1*B*) (18, 19, 28). Nevertheless, direct experimental evidence on the substrates of LigG activity is lacking.

Here, we determine the stereochemical properties of GSH-dependent enzymes in the Lig pathway. We show that the GST family members LigE, LigP, and LigF are each β-etherases and that LigG is a β-thioetherase, and we identify previously uncharacterized pathway intermediates. We demonstrate that the GST family members LigE, LigF, and LigP each catalyze stereospecific cleavage of α-keto-β-aryl ethers, β(*R*)GVG (Fig. 1*A*) or β(*S*)GVG (Fig. 1*B*). We further show that each GST β-etherase is stereoselective, yielding guaiacol and one of two potential β-epimers of GS-βVG. We also demonstrate that LigG is a β-*S*-thioetherase catalyzing the GSH-dependent cleavage of GS-βVG to β-deoxy-α-veratrylglycerone (18). To rationalize the observed stereochemical properties of these proteins, we present a model that links the observed substrate specificity of LigE, LigF, LigP, and LigG to the racemic nature of the lignin polymer.

## EXPERIMENTAL PROCEDURES

### Gene Cloning

#### 

##### General

Manipulation of DNA and preparation of *Escherichia coli* transformant cells were carried out according to standard methods (29). All DNA primers were obtained commercially from Integrated DNA Technologies. All restriction and DNA ligation enzymes were purchased from New England Biolabs. Plasmid pMHTΔ238 was obtained from the DNASU Plasmid Repository. All plasmids with the pM (first two letters) naming convention (*i.e.* all plasmids encoding *Sphingobium* sp. strain SYK-6 Lig enzymes and *Vibrio cholerae* RtxA protease) were obtained commercially from GeneArt® (Invitrogen) and were codon-optimized for expression in *E. coli*.

##### Preparation of Vector pVP102K

Preparation of expression vector pVP102K required the creation of intermediate plasmids pVP100K and pVP101K as follows. Plasmid pVP100K was first constructed by excising a 1,110-bp fragment from pMK1157550 with restriction enzymes BseRI and HindIII and then ligating into the compatible ends at the AsiSI and HindIII sites of pVP80K (obtained from the DNASU Plasmid Repository). Intermediate plasmid pVP101K was then constructed by excising a 852-bp fragment from the 6,123-bp plasmid pVP100K using AsiSI and ligating the free ends together to form 5,271-bp plasmid pVP101K. Expression vector pVP102K was then constructed by amplifying a 4,169-bp fragment from plasmid pVP101K, restricting the resulting amplicon with SacI, and ligating the free ends together, resulting in removal of the maltose-binding protein-encoding region of pVP101K and formation of the 4,153-bp expression vector pVP102K, which was T5 promoter-inducible and contained an in-frame N-terminal octahistidine (NHis_8_) affinity tag and tobacco etch virus (Tev) protease cleavage site upstream of the translation start codon.

##### Preparation of Vector pVP202K

Intermediate 4,078-bp plasmid pVP201K was constructed via excision of a 75-bp fragment encoding the NHis_8_ region of pVP102K by NcoI restriction and subsequent ligation. A 701-bp cysteine protease domain-encoding region of the *V. cholerae* RtxA protease was then amplified from pMA1157486, both the amplicon and vector pVP201K were restricted with SacII and PmeI, and the 681-bp and 4,037-bp fragments were ligated together to form the 4,718-bp expression vector pVP202K, which contained the C-terminal inducible RtxA protease fused to an NHis_8_ affinity tag upstream of the translation stop codon.

##### Preparation of the pVP202KSSLigE Expression Plasmid

The ORF encoding *Sphingobium* sp. strain SYK-6 LigE on plasmid pMKT1025977 was cloned into expression vector pVP202K by the PCR overlap method (30–33). The first round of PCR yielded a 906-bp amplicon, which was used to prime plasmid pVP202K in the second round of PCR, affording the 5,470-bp plasmid pVP202KSSLigE, which was used for expression of LigE.

##### Preparation of the pVP202KSSLigP Expression Plasmid

A 902-bp fragment was amplified from plasmid pJBEI_E2/P (obtained from collaborators at the Joint BioEnergy Institute, Berkeley, CA) and was cloned into vector pVP202K by the PCR overlap method (30–33), affording plasmid pVP202KSSLigP, which was used for the expression of LigP.

##### Preparation of the pVP102KSSLigF Expression Plasmid

A 822-bp fragment containing the LigF-encoding ORF was amplified from plasmid pMKT1025979, the amplicon was restricted with AsiSI and SacII, and an 809-bp fragment was inserted into the AsiI-SacII region of pVP102K, plasmid pVP102KSSLigF, which was used for the expression of LigF.

##### Preparation of the pVP102KSSLigG Expression Plasmid

A 849-bp fragment containing the LigG-encoding ORF from plasmid pMK1118986 was amplified, both the amplicon and vector pVP102K were restricted with AsiSI and SacII, and the 836-bp and 4,067-bp fragments were ligated together, forming the 4,093-bp plasmid pVP102KSSLigG, which was used for the expression of LigG.

### Enzyme Purification

#### 

##### General

Tranformant cultures of *E. coli* strain B834 were grown aerobically with shaking at 37 °C in Luria-Bertani (LB) medium (34, 35) overnight and subcultured (1:100) into 1 liter of autoinduction ZYM-5052 medium (36) supplemented with 100 μg ml^−1^ kanamycin. Autoinducing cultures were grown overnight at 25 °C and then harvested by centrifugation at 10,500 × *g* with a Sorvall RC-5B Superspeed Centrifuge. Pelleted cells were resuspended in ∼30 ml (or 2.5 ml of buffer/g of cells) of Buffer A (50 mm NaH_2_PO_4_, 100 mm NaCl, 10 mm imidazole, 10% glycerol, 1.0% Triton X-100, 0.5 mm *tris*-(2-carboxyethyl)-phosphine hydrochloride (TCEP), pH 8.0) supplemented with 1 mg ml^−1^ lysozyme. Cells were incubated with lysozyme for 30 min at 4 °C, after which time the NaCl concentration was brought up to 300 mm using a 5 m NaCl solution, and the slurry was augmented with 10 μg ml^−1^ RNase A and 5 μg ml^−1^ DNase I and allowed to sit for 15 min. Cells were then subjected twice to compression through a Spectronic Instruments French pressure cell at 137 megapascals, and cell debris was removed by centrifugation at 30,000 × *g*. Histidine-tagged proteins were then purified from cell lysates through a series of chromatographic and size exclusion chromatographic separations using a GE Healthcare ÄKTA Prime Plus FPLC system. First, cell lysates were loaded at 2 ml min^−1^ onto a GE Healthcare XK-16 column packed with 25 ml of Qiagen nickel-nitrilotriacetic acid (Ni-NTA) resin. The column was then washed with 100 ml of Buffer B (50 mm NaH_2_PO_4_, 300 mm NaCl, 25 mm imidazole, 0.5 mm TCEP, pH 8.0), and His-tagged proteins were then eluted with a 30-ml linear gradient to 100% Buffer C (50 mm NaH_2_PO_4_, 300 mm NaCl, 500 mm imidazole, 0.5 mm TCEP, pH 8.0). Collected fractions were then dialyzed for ∼12 h at 4 °C using a 10,000 molecular weight cut-off Thermo Scientific Slide-a-Lyzer dialysis cassette into Buffer D (50 mm NaH_2_PO_4_, 50 mm NaCl, 10 mm imidazole, 0.5 mm TCEP, pH 8.0). Enzyme-specific methods (see below) were then carried out for the removal of recombinant affinity tags. After proteolysis, affinity tags were removed from the preparation by passage over a second GE Healthcare XK-16 column packed with 30 ml of Qiagen Ni-NTA resin. The preparation was then subjected to size exclusion chromatography in Buffer E (10 mm HEPES, 100 mm NaCl, pH 7.7) at a flow rate of 1 ml min^−1^ using a GE Healthcare HiLoad 16/600 Superdex^TM^ 200 pg column. Finally, each enzyme was concentrated in Buffer E to ≥10 mg ml^−1^ by centrifugation at 3,500 × *g* in a 10,000 molecular weight cut-off Pierce concentrator before drop freezing in liquid N_2_. Protein concentrations were determined by the Bradford method (37).

##### Purification of Tev Protease

Tev protease was expressed as a 70.8-kDa fusion to maltose-binding protein (38). To ensure full removal of the 42.6-kDa maltose binding domain by autoproteolysis, cell lysates were allowed to sit for ∼2 h between disruption by French press and Ni-NTA affinity chromatography. Tev protease was then subjected to size exclusion chromatography, where it eluted as a monomeric 28.2-kDa enzyme.

##### Purification of LigE and LigP

LigE and LigP were expressed as either a 56.1-kDa or 55.0-kDa fusion to RtxA and were dialyzed into Buffer D after Ni-NTA affinity chromatography. The RtxA and CHis_8_ tag were then cleaved through 100 μm inositol hexaphosphate induction of RtxA (39–42). The sample was subjected to a second round of Ni-NTA chromatography for removal of the 24.0-kDa RtxA-CHis_8_ domain. SDS-12% PAGE of denatured 32.1-kDa LigE and 31.0-kDa LigP enzymes showed that each preparation was homogeneous (data not shown).

##### Purification of LigF and LigG

NHis_8_-fused recombinant enzymes LigF (33.4 kDa) and LigG (34.1 kDa) were expressed with the amino acid motif for the consensus cut site of Tev protease ENLYFQ (where cleavage occurs after Q) encoded immediately after the N-terminal His tag and prior to the encoding sequence of the respective enzyme (43–46). After the first round of Ni-NTA chromatography, the NHis_8_-tagged enzymes were dialyzed into Buffer D for cleavage of the affinity tags using Tev protease (2 mg of TevΔ238 per 100 mg of recombinant enzyme). Subsequently, the samples were subjected to a second round of Ni-NTA affinity as well as size exclusion chromatography. SDS-12% PAGE of denatured 30.0-kDa LigF and 30.6-kDa LigG preparations showed that each was homogeneous (data not shown).

### NMR Spectroscopy

^1^H and ^13^C NMR spectra were recorded on a Bruker Biospin (Billerica, MA) AVANCE 700-MHz spectrometer fitted with a cryogenically cooled 5-mm TXI gradient probe with inverse geometry (proton coils closest to the sample). All compounds were characterized and assigned using the usual array of homonuclear and heteronuclear two-dimensional correlation (primarily COSY, HSQC, and HMBC) experiments.

### Synthesis of β-Ether-linked Model Compounds

#### 

##### Synthesis of βGVE

**FIGURE 2.**
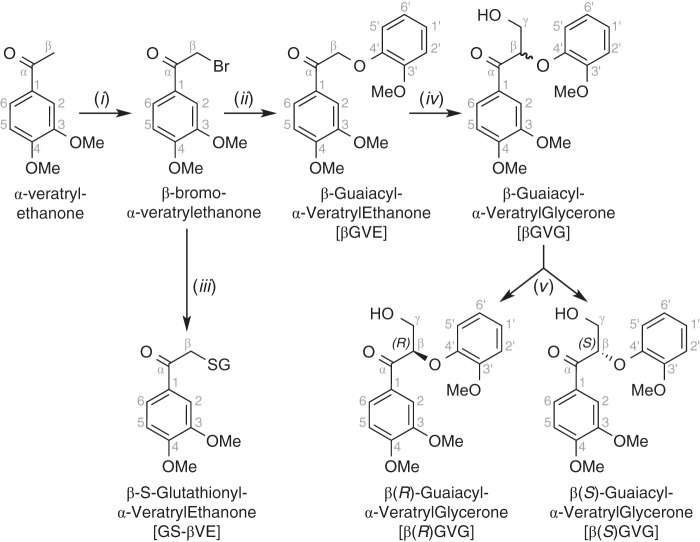
**Scheme for the synthesis of βGVE, GS-βVE, and β-guaiacyl-α-veratrylglycerone (β(*R*)GVG and β(*S*)GVG).** Reagents and conditions were as follows: pyridinium tribromide, EtOAc, 30 min, 59% (*i*); guaiacol, K_2_CO_3_, acetone, 4 h, 82% (*ii*); glutathione, NaHCO_3_, 1:1 acetone/water, 14 h, 100% (*iii*); formaldehyde, K_2_CO_3_, 1,4-dioxane, 3 h, 88% (*iv*); chiral chromatography, ethanol/hexane (*v*). See supplemental material for details.

The synthesis of βGVE (Fig. 2) began with bromination of commercially available α-veratrylethanone to produce crystalline β-bromo-α-veratrylethanone. β-Ether formation was via S_N_2 displacement of the bromide by the phenolate ion of guaiacol, affording crystalline βGVE (47).

##### Synthesis of βGVG

βGVE was condensed with formaldehyde to yield racemic βGVG from which enantiopure β(*R*)GVG and β(*S*)GVG were isolated by preparative chiral chromatography. Additional synthetic details can be found in the supplemental material.

### Synthesis of β-Thioether-linked Model Compounds

#### 

##### Synthesis of GS-βVE

The synthesis of GS-βVE (Fig. 2) commenced with chemically synthesized β-bromo-α-veratrylethanone and guaiacol as starting materials. β-Thioether formation was via S_N_2 displacement of the bromide by the thiolate ion of GSH, yielding GS-βVE, which was then purified by preparative C_18_-reversed phase chromatography.

##### Synthesis of GS-βVG

**FIGURE 3.**
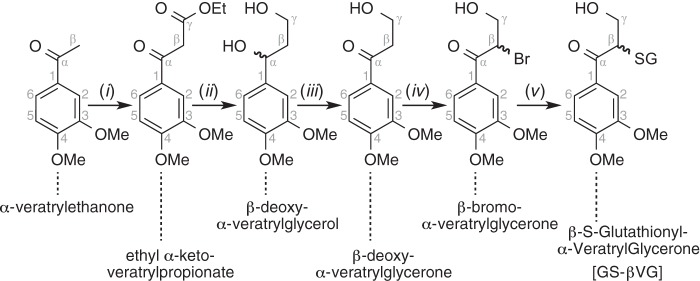
**Scheme for the synthesis of GS-βVG and β-deoxy-α-veratrylglycerone.** Reagents and conditions were as follows: diethyl carbonate, NaH, THF, reflux, 2 h, 88% (*i*); DIBAL-H, THF, 2 h, 91% (*ii*); DDQ, 1,4-dioxane, 30 min, flash chromatography, 74% (*iii*); pyridinium tribromide, EtOAc, 30 min, flash chromatography, 27% (*iv*); glutathione, NaHCO_3_, 1:1 acetone/water, 14 h, 100% (*v*). See supplemental material for details.

Condensation of commercially available α-veratrylethanone with diethyl carbonate yielded ethyl α-keto-veratrylpropionate (Fig. 3). Its reduction produced β-deoxy-α-veratrylglycerol, which was converted to β-deoxy-α-veratrylglycerone through benzylic oxidation. Next, bromination yielded β-bromo-α-veratrylglycerone. Thioether formation with GSH then afforded the desired β-epimers of the GSH conjugate GS-βVG, which was then purified by preparative C_18_-reversed phase chromatography. Additional synthetic details can be found in the supplemental material.

### Synthesis of CS-β(S)VP and CS-β(R)VP

#### 

##### Synthesis of 3,4-Dimethoxycinnamaldehyde Dimethyl Acetal

**FIGURE 4.**
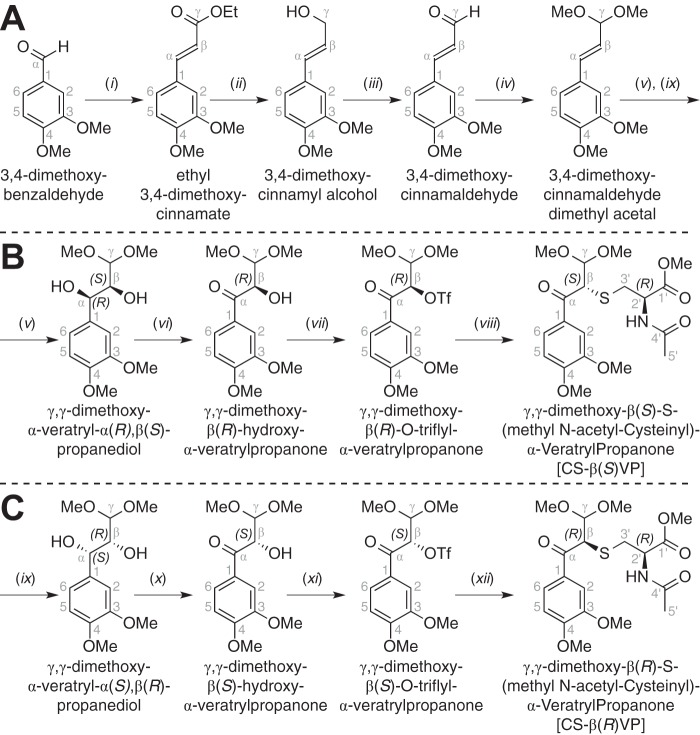
**Scheme for the synthesis of 3,4-dimethoxycinnamaldehyde dimethyl acetal (*A*), CS-β(*S*)VP (*B*), and CS-β(*R*)VP (*C*).** Reagents and conditions were as follows: triethyl phosphonoacetate, NaH, THF, 2 h, 91% (*i*); DIBAL-H, THF, 2 h, 89% (*ii*); DDQ, 1,4-dioxane, 30 min, flash chromatography, 72% (*iii*); *p*-toluenesulfonic acid, trimethyl orthoformate, MeOH, 2 h, 96% (*iv*); AD-mix β, methanesulfonamide, 1:1 *t*-butanol/water, 4 °C, 18 h, 72% (*v*); DDQ, 1,4-dioxane, 30 min, flash chromatography, 73% (*vi*); trifluoromethanesulfonic anhydride, 2,6-lutidine, CH_2_Cl_2_, 2 h, flash chromatography, 75% (*vii*); methyl *N*-acetyl-(*R*)-cysteinate, K_2_CO_3_, dimethyl formamide, 2 h, 53% (*viii*); AD-mix α, methanesulfonamide, 1:1 *t*-butanol/water, 4 °C, 18 h, 83% (*ix*); DDQ, 1,4-dioxane, 30 min, flash chromatography, 81% (*x*); trifluoromethanesulfonic anhydride, 2,6-lutidine, CH_2_Cl_2_, 2 h, flash chromatography, 59% (*xi*); methyl *N*-acetyl-(*R*)-cysteinate, K_2_CO_3_, dimethyl formamide, 2 h, 73%. See supplemental material for details.

Synthesis of the dimethyl acetal (Fig. 4*A*) commenced with the condensation of triethyl phosphonoacetate with 3,4-dimethoxybenzaldehyde, yielding ethyl 3,4-dimethoxycinnamate. Reduction of the ester yielded 3,4-dimethoxycinnamyl alcohol. Oxidation of the ring-conjugated alcohol afforded 3,4-dimethoxycinnamaldehyde. Protection of the carbonyl group then yielded 3,4-dimethoxycinnamaldehyde dimethyl acetal.

##### Synthesis of CS-β(S)VP and CS-β(R)VP

Syntheses of β(*S*)- and β(*R*)-isomers of γ,γ-dimethoxy-β-*S*-(methyl *N*-acetyl cysteinyl)-α-veratrylpropanone (CS-β(*S*)VP and CS-β(*R*)VP) were carried out in parallel (Fig. 4, *B* and *C*), and each thioether was derived from 3,4-dimethoxycinnamaldehyde dimethyl acetal. Then, in separate parallel reactions, the alkene was stereoselectively oxidized to diols γ,γ-dimethoxy-α-veratryl-α(*R*),β(*S*)-propanediol and γ,γ-dimethoxy-α-veratryl-α(*S*),β(*R*)-propanediol. Benzylic oxidation of the diols then yielded ketones γ,γ-dimethoxy-β(*R*)-hydroxy-α-veratrylpropanone and γ,γ-dimethoxy-β(*S*)-hydroxy-α-veratrylpropanone. Triflation of the β-hydroxyls afforded triflate esters γ,γ-di-methoxy-β(*R*)-O-triflyl-α-veratrylpropanone and γ,γ-di-methoxy-β(*S*)-O-triflyl-α-veratrylpropanone. Last, S_N_2 displacement of the triflate groups by the thiolate ion of methyl *N*-acetyl cysteinate yielded β(*S*)- and β(*R*)-isomers of γ,γ-di-methoxy-β-*S*-(methyl *N*-acetyl cysteinyl)-α-veratrylpropanone (CS-β(*S*)VP and CS-β(*R*)VP) with high diastereometric purity. Because S_N_1 displacement of the β-*O*-triflate synthetic intermediates would have yielded a mixture of both CS-βVP β-epimers in the synthesis schemes of both CS-β(*S*)VP (Fig. 4*B*) and CS-β(*R*)VP (Fig. 4*C*), we conclude that formation of the β-*S*-thioether linkages in CS-β(*S*)VP and CS-β(*R*)VP was governed by S_N_2 inversion of the chiral center at β, affording highly diastereometrically pure products.

### Chromatographic Techniques

#### 

##### General

All chromatographic separations were carried out using a Beckman 125NM solvent delivery module equipped with a Beckman 168 UV detector.

##### Preparative C_18_-reversed Phase Chromatography

A prepacked Biotage KP-C_18_ (100 g) reversed phase column was used for the purification of all enzymatically synthesized β-*S*-thioether compounds (GS-βVG and GS-βVE). A mixture of water and methanol was used for the mobile phase at a flow rate of 10 ml min^−1^. The proportions of the total flow made up by each buffer were adjusted over a gradient: 0–15 min, 0% methanol; 15–20 min, gradient from 0 to 100% methanol; 20–35 min, 100% methanol; 35–40 min, gradient from 100 to 0% methanol; 40–50 min, 0% methanol. Fractions with UV absorption at 280 nm were collected, pooled, dried under a stream of nitrogen gas, and analyzed by ^1^H, ^13^C, COSY, HSQC, and HMBC NMR spectroscopy.

##### Analytical C_18_-reversed Phase Chromatography

**FIGURE 5.**
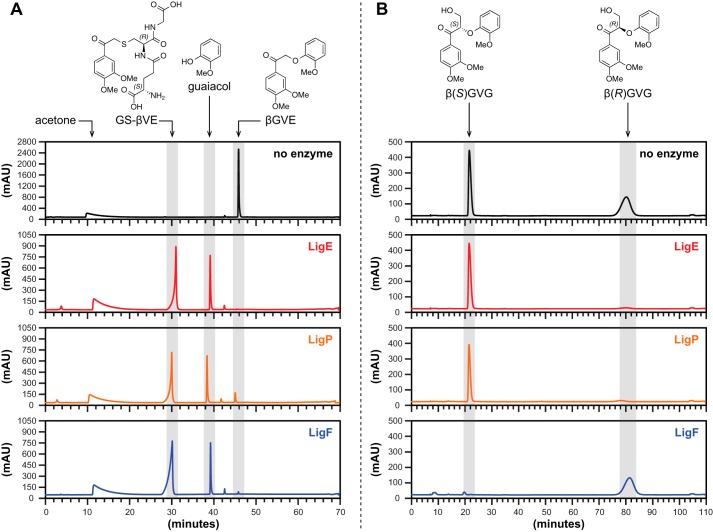
**HPLC chromatogram traces of pre-enzyme addition (black), LigE (*red*), LigP (*orange*), and LigF (*blue*) enzyme assay samples.** *A*, C_18_-reversed phase chromatography of βGVE prior to the addition of and after a 1-h incubation with GSH and either LigE, LigP, or LigF. *B*, chiral chromatography of *racem*-βGVG prior to the addition of and after a 1-h incubation with GSH and either LigE, LigP, or LigF.

Parallel β-etherase reactions were carried out using either LigE, LigP, or LigF (1.0 mg ml^−1^) with glutathione (2.0 mm) and βGVE (1.5 mm) as cosubstrates. Prior to the addition of enzyme, 0.3 ml of the reaction mixture containing βGVE was collected for analysis by C_18_-reversed phase chromatography. After 1 h of incubation with one of the β-etherases, 0.3 ml of the reaction mixture was also collected for HPLC analysis. The 0.3-ml samples from 0 h and 1 h reaction times were then injected into an Ultra Aqueous (Restek Corp., Bellefonte, PA) C_18_-reversed stationary phase column (4 × 120 mm) for separation of GS-βVE, guaiacol, and βGVE (Fig. 5*A*). A mixture of water and methanol was used as the mobile phase at a flow rate of 1.0 ml min^−1^. The methanol fraction of the total flow (with water as the remainder) was adjusted over a gradient as follows: 0–15 min, 0% methanol; 15–40 min, gradient from 0 to 100% methanol; 40–55 min, 100% methanol; 55–60 min, gradient from 100 to 0% methanol; 60–70 min, 0% methanol. GS-βVE, guaiacol, and substrate βGVE eluted from the C_18_ column after retention times (*t_R_*) = 30.5, 39.6, and 46.3 min, respectively.

##### Preparative Chiral Chromatography

To separate chiral enantiomers β(*R*)GVG and β(*S*)GVG, crystalline racemic βGVG (2 mg, 6.0 nmol) was dissolved in ethanol (5 ml) and injected into a CHIRALPAK AY-H column (10 × 250 mm). A mixture of ethanol and hexane was used as the mobile phase at a flow rate of 2.0 ml min^−1^. The ethanol fraction of the total flow (with hexane as the remainder) was adjusted over a gradient as follows: 0–12 min, 50% ethanol; 12–20 min, gradient from 50 to 100% ethanol; 20–90 min, 100% ethanol; 90–95 min, gradient from 100 to 50% ethanol; 95–110 min, 0% ethanol. Fractions containing βGVG enantiomers were collected and pooled, and solvents were dried *in vacuo*. β(*S*)GVG or β(*R*)GVG eluted from the column with *t_R_* = 21.8 and 80.6 min. The aforementioned procedure was repeated four additional times in order to collect ∼5 mg of each enantiomer, to be used for enzymatic syntheses and isolation of GS-βVE, GS-β(*S*)VG, and GS-β(*R*)VG.

##### Analytical Chiral Chromatography

Parallel β-etherase reactions were carried out with either LigE, LigP, or LigF (1.0 mg ml^−1^) with glutathione (2.0 mm) and racemic βGVG (1.5 mm) as cosubstrates. Aliquots (0.5 ml) were collected prior to the addition of enzyme (0-h sample) and again after 1 h of incubation with either LigE, LigP, or LigF. The 0.5-ml samples from 0 and 1 h were then each extracted six times with ethyl acetate, and the solvent was subsequently dried *in vacuo*. Residues from the 0- and 1-h samples were then dissolved in 0.1 ml of ethanol and injected into a Diacel Chemical Industries CHIRALPAK AY-H column (10 × 250 mm) for separation of guaiacol and chiral enantiomers β(*R*)GVG and β(*S*)GVG (Fig. 5*B*). A mixture of ethanol and hexane was used as the mobile phase at a flow rate of 2.0 ml min^−1^. The ethanol fraction of the total flow (the remainder was hexane) was adjusted over a gradient as follows: 0–12 min, 50% ethanol; 12–20 min, gradient from 50 to 100% ethanol; 20–90 min, 100% ethanol; 90–95 min, gradient from 100 to 50% ethanol; 95–110 min, 0% ethanol. Guaiacol, β(*S*)GVG, and β(*R*)GVG eluted from the chiral column with *t_R_* = 8.0, 21.8, and 80.6 min, respectively.

### Isolation of Enzymatic Reaction Products

#### 

##### General

*In vitro* reaction assays for enzymatic synthesis of β-*S*-thioethers were conducted in an aqueous assay buffer (10 mm HEPES, 60 mm NaCl, 100 μm TCEP, 5% acetone, 2 mm GSH, pH 7.5). GSH was added just prior to each assay to avoid disulfide formation, and NaOH was used to readjust the buffer to pH 7.5. All aqueous phase reaction products were desalted and purified by preparative C_18_-reversed phase HPLC, and fractions containing the reaction products were collected and dried over a stream of nitrogen gas. The ^1^H, ^13^C, COSY, HSQC, and HMBC NMR spectra were analyzed for each of the isolated reaction products.

##### In Vitro Enzymatic Synthesis of GS-β(S)VG from LigE or LigP

To 10 ml of the enzyme assay buffer, β(*R*)GVG (3.3 mg, 1.0 mm) was added, and the buffer was separated into two 5-ml aliquots. To one 5-ml aliquot, LigE (1.0 mg, 6.2 μm) was added. LigP (1.0 mg, 6.5 μm) was added to the second 5-ml aliquot. Both reactions were incubated at room temperature for a period of 1 h. Guaiacol and trace amounts of β(*R*)GVG were removed from the reaction mixture by six successive ethyl acetate extractions. The LigE and LigP reaction products eluted from the Biotage KP-C18 column with *t_R_* = 30.9 and *t_R_* = 31.0 min, respectively.

##### In Vitro Enzymatic Synthesis of GS-β(R)VG from LigF

Aside from the use of β(*S*)GVG as the LigF substrate, reaction conditions identical to those used for LigE and LigP were applied for LigF-catalyzed synthesis of GS-β(*R*)VG from β(*S*)GVG. GS-β(*R*)VG eluted from the Biotage KP-C18 column with *t_R_* = 31.1 min.

##### In Vitro Enzymatic Synthesis of GS-βVE from LigE, LigP, and LigF

To 15 ml of the enzyme assay buffer, βGVE (4.5 mg, 1.0 mm) was added, and the buffer was separated into three 5-ml aliquots. To each, either LigE (1.0 mg, 6.2 μm), LigP (1.0 mg, 6.5 μm), or LigF (1.0 mg, 6.7 μm) was added. The three reactions were incubated at room temperature for 1 h. After ethyl acetate extraction, the aqueous reaction products of LigE, LigP, and LigF eluted from the Biotage KP-C18 column after *t_R_* = 31.0, 31.1, and 31.0 min, respectively.

##### In Vitro Enzymatic Synthesis of β-Deoxy-α-veratrylglycerone and α-Veratrylethanone from LigG

To separate 5-ml aliquots of enzyme assay buffer, LigG (1.3 mg, 8.5 μm) and either chemically synthesized GS-βVG (5.0 mg, 1.0 mm each) or GS-βVE (2.4 mg, 1.0 mm) were added, and the buffer was readjusted to pH 7.5 using NaOH. Reactions were incubated at room temperature for 1 h. Six ethyl acetate extractions were carried out, and the organic fractions were pooled. Solvent was then removed by evaporation *in vacuo*, and products (either β-deoxy-α-veratrylglycerone or α-veratrylethanone) were analyzed by NMR spectroscopy (^1^H and ^13^C NMR spectra can be found in the supplemental material. When GS-βVG was used as the substrate, the residual GS-β(*S*)VG in the aqueous fraction was purified further by preparative C_18_-reversed phase HPLC, where GS-β(*S*)VG eluted from the Biotage KP-C18 column with *t_R_* = 31.0 min.

## RESULTS

### 

#### 

##### Characterization of the Etherase Reaction Products Using an Achiral Substrate

The GST family member LigE from strain SYK-6 liberates 4′-methylumbelliferone from the achiral substrate (β-*O*-7′-(4′-methyl)-umbelliferyl-α-guaiacyl-ethanone) (14, 17). This prompted us to use a synthetic achiral substrate (βGVE) (Fig. 1*C*) in assays with the three proposed β-etherases from this strain. We also synthesized (Fig. 2) a β-*S*-glutathione-conjugated model compound, GS-βVE, that is a potential βGVE cleavage product.

When we incubated βGVE with pure recombinant LigE, LigF, or LigP, we found that, as expected from members of this GST superfamily, the substrate's degradation required the presence of GSH (15). In the presence of GSH, degradation of βGVE by each enzyme (Fig. 5*A*) was accompanied by production of guaiacol and an unidentified compound (later identified as GS-βVE). Thus, we conclude that LigE, LigP, and LigF were each active on the achiral βGVE, as predicted (Fig. 1*C*).

**FIGURE 6.**
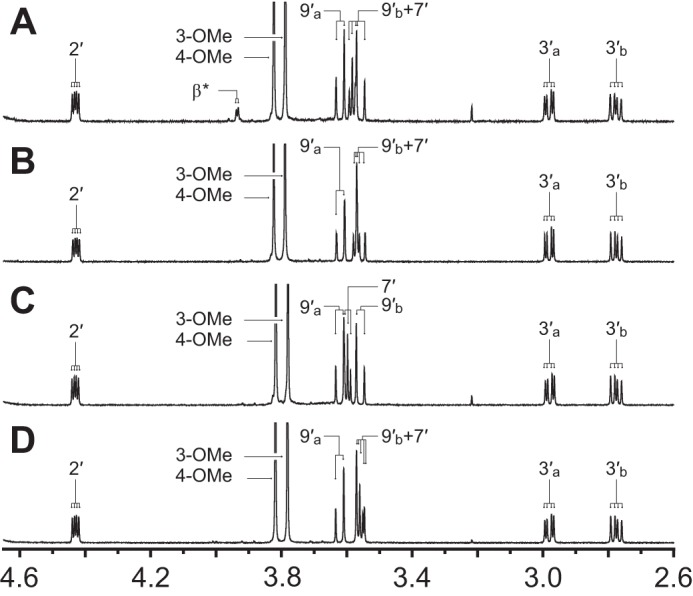
**Aligned ^1^H NMR partial spectra (2.60–4.65 ppm) of the GS conjugate, GS-βVE, in D_2_O.** Proton assignment labels correspond with the carbon to which the proton is bound. *Alphabetical subscripts* differentiate two non-identical geminal protons. Proton peaks that did not integrate as expected are denoted with an *asterisk* (see “Results”). *A*, compound GS-βVE obtained via chemical synthesis. *B–D*, reaction product GS-βVE isolated from LigE (*B*), LigP (*C*), or LigF (*D*) activities using the achiral model compound GβVE as a substrate.

**FIGURE 7.**
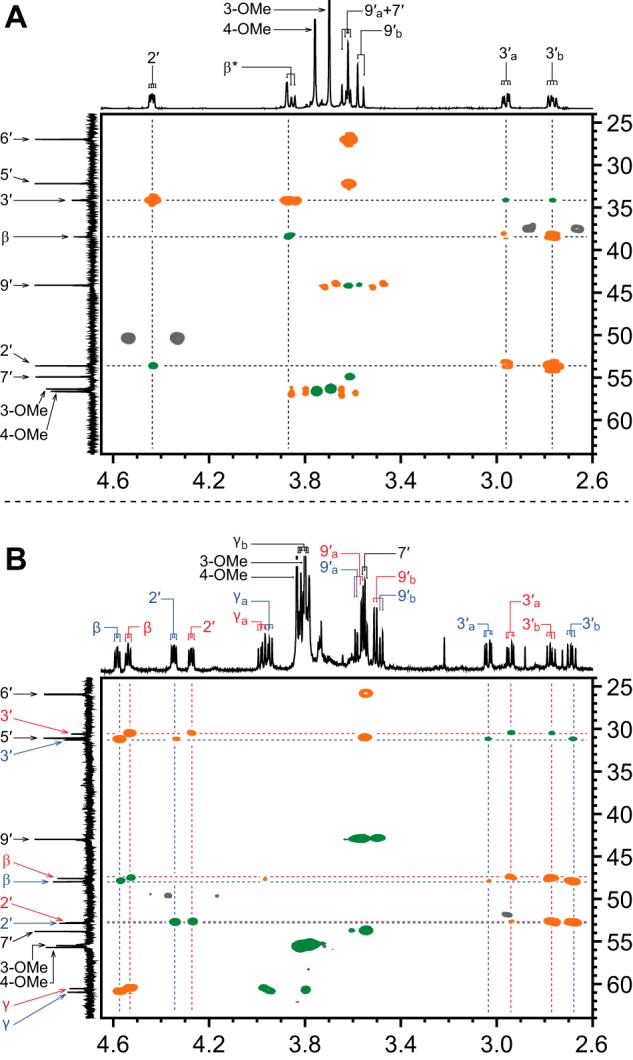
**Partial ^1^H-^13^C two-dimensional HSQC (*green*) and HMBC (*orange*) NMR spectra of β-*S*-thioether-linked compounds in D_2_O, where ^1^H chemical shifts are plotted on the *x* axis (2.60–4.65 ppm), ^13^C chemical shifts are plotted on the *y* axis (24.0–64.0 ppm), and non-^1^H-^13^C-correlating HMBC spectral regions are indicated (*gray*).** Proton assignment labels correspond with the carbon to which the proton is bound. Alphabetical subscripts differentiate two non-identical geminal protons. *A*, chemically synthesized GS-βVE. Proton peaks that did not integrate as expected are denoted with an *asterisk* (see “Results”). *B*, chemically synthesized mixture of GS-β(*S*)VG (*red labels*) and GS-β(*R*)VG (*blue labels*). Overlapping GS-β(*S*)VG and GS-β(*R*)VG ^1^H and ^13^C spectral regions are indicated (*black labels*).

The unidentified product of βGVE degradation from each Lig enzyme was purified for analysis by NMR spectroscopy (supplemental material). The ^1^H NMR spectrum of the product isolated from either LigE, LigP, or LigF reactions using βGVE and GSH (Fig. 6, *B–D*) was identical to that of chemically synthesized GS-βVE (Fig. 6*A*). In addition, long range two-dimensional ^1^H-^13^C NMR (HMBC) correlations (*a*) between the β-protons and the 3′-carbon as well as (*b*) between carbon-β and each 3′H_a_ and 3′H_b_ proton in synthetic GS-βVE (Fig. 7*A*) confirm that the GSH and aromatic moieties are linked via a β-*S*-3′-thioether. The task of identifying carbon-β and the β-protons was complicated by the β-protons' exchange with solvent (D_2_O) deuterons. By analyzing the spectra of chemically synthesized GS-βVE in 90/10% H_2_O/D_2_O as the NMR solvent (with suppression of H_2_O proton signals), we were able to unambiguously assign the β-carbon in the ^13^C spectrum and the β-protons in the ^1^H spectrum (see supplemental material). The HMBC spectrum in this solvent also revealed the above correlations between the β and 3′ nuclei, further establishing the existence of the β-*S*-3′-thioether linkage. Furthermore, both methoxyl moieties (3-OMe and 4-OMe) in GS-βVE could be unambiguously assigned (Fig. 6), along with all aromatics, allowing GβVE β-ether bond cleavage by each GST family member to be fully elucidated. From these results, we conclude that LigE, LigF, and LigP each catalyze GSH-dependent β-etherase activity with achiral substrate GβVE and that β-etherase-catalyzed conversion of βGVE to GS-βVE (Fig. 1*C*) links the β-carbon of the aryl moiety to GSH.

##### β-S-Thioetherase Activity of LigG with the Product of β-Etherase Activity

The identification of GS-βVE as the product of β-etherase activity by the GST family members LigE, LigF, and LigP allowed us to test if this compound is a substrate for the putative GSH-dependent β-*S*-thioetherase, LigG. We incubated LigG from strain SYK-6 with GSH and chemically synthesized GS-βVE and observed GSH-dependent cleavage of GS-βVE and formation of the expected aromatic reaction product α-veratrylethanone (18) (see supplemental material). We therefore conclude that LigG is a GSH-dependent β-*S*-thioetherase that cleaves the GSH conjugate GS-βVE, the intermediate derived from β-etherase activity with achiral substrate βGVE (Fig. 1*C*).

The use of the achiral substrate βGVE documents the combined activities of the GSH-dependent enzymes LigE, LigF, LigP, and LigG in a β-etherase pathway that releases two aromatic monomers from a dimeric model substrate (Fig. 1*C*). In addition, we provide direct experimental evidence that LigG catalyzes thioether cleavage at the β-*S*-glutathionyl linkage in the presence of GSH.

##### Stereoselectivity of the LigE, LigP, and LigF β-Etherases

**FIGURE 8.**
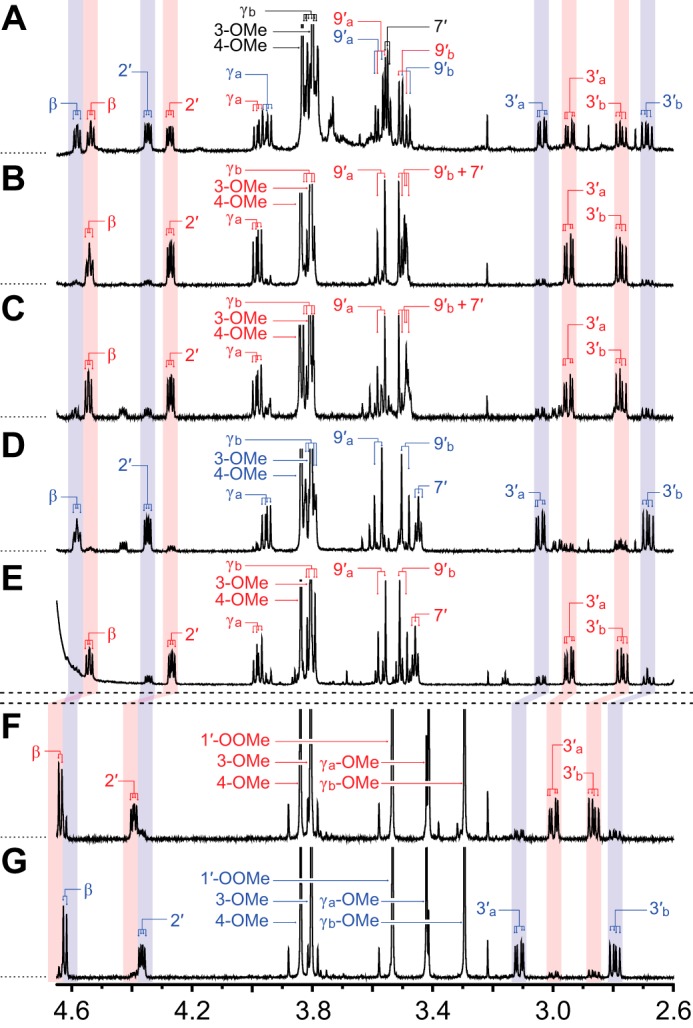
**Aligned ^1^H NMR partial spectra (2.60–4.65 ppm) of both β-epimers of β-*S*-glutathionyl-α-veratrylglycerone (GS-β(*S*)VG and GS-β(*R*)VG) and both β-epimers of γ,γ-dimethoxy-β-*S*-(methyl *N*-acetyl-cysteinyl)-α-veratrylpropanone (CS-β(*S*)VP and CS-β(*R*)VP) in D_2_O.** Proton assignment labels correspond with the carbon to which the proton is bound. Alphabetical subscripts differentiate two non-identical geminal protons or methoxyls. *Red labels* and *shading* are used for the two β(*S*)-configured compounds, GS-β(*S*)VG and CS-β(*S*)VP; *blue labels* and *shading* are used for the two β(*R*)-configured compounds, GS-β(*R*)VG and CS-β(*R*)VP; *black labels* are used for overlapped regions. Regions that differentiate β(*S*)- and β(*R*)-configurations (*i.e.* protons at β, 2′, and 3′) are *shaded across panels. A*, chemically synthesized mixture (1:1) of GS-β(*S*)VG and GS-β(*R*)VG. *B*, GS-β(*S*)VG from LigE. *C*, GS-β(*S*)VG from LigP. *D*, GS-β(*R*)VG from LigF. *E*, residual GS-β(*S*)VG not degraded by LigG. *F*, chemically synthesized CS-β(*S*)VP. *G*, Chemically synthesized CS-β(*R*)VP.

Given the racemic nature of β-ether units in the lignin backbone (21, 22), we analyzed activities of the β-etherases with chemically synthesized βGVG, a substrate, like its lignin counterpart, with a chiral center at the β-position and two enantiomeric configurations, β(*R*)GVG (Fig. 1*A*) and β(*S*)GVG (Fig. 1*B*). To aid these studies, we also synthesized (Fig. 3) the GSH conjugate, β-*S*-glutathionyl-α-veratrylglycerone (GS-βVG), which is the predicted product of β-etherase activity with βGVG. The existence of two β-epimers (GS-β(*S*)VG and GS-β(*R*)VG) in chemically synthesized GS-βVG was demonstrated by ^1^H-^1^H COSY, HSQC, and ^1^H NMR spectral analyses (Fig. 8*A*); the HMBC spectrum confirmed the existence of the β-*S*-3′-thioether linkage (Fig. 7*B*).

To test for β-etherase activity, the racemic substrate βGVG was incubated with GSH and either LigE, LigP, or LigF. At the end of the assay, samples were extracted with ethyl acetate, partitioning guaiacol and residual βGVG enantiomers to the organic layer and β-epimers of GS-βVG to the aqueous layer. After evaporation of ethyl acetate and guaiacol *in vacuo*, residual organics were dissolved in ethanol and analyzed by chiral chromatography to determine if the enzymes cleaved one or both of the substrate enantiomers (Fig. 5*B*). We found that LigF cleaved only the β(*S*)GVG enantiomer, whereas LigE and LigP each cleaved only the β(*R*)GVG isomer. This confirmed that each of these enzymes is stereospecific for a single enantiomer (18, 26).

To further characterize the products of β-etherase activity, we analyzed their activities when either enantiopure β(*R*)GVG or β(*S*)GVG (purified by preparative chiral chromatography) was incubated with GSH and either LigE, LigP, or LigF. We found that LigE converted β(*R*)GVG to GS-β(*S*)VG with a 13:1 molar excess over the other diastereomer (GS-β(*R*)VG), as calculated from integration of the ^1^H NMR spectral peaks (Fig. 8*B*). LigP exhibited similar stereoselectivity because the product of β(*R*)GVG cleavage was a ∼4:1:1 mixture of GS-β(*S*)VG, GS-β(*R*)VG, and an impurity likely to be a β,γ-unsaturated alkene (Fig. 8*C*). In contrast, the product of LigF-catalyzed cleavage of β(*S*)GVG was a 1:7:2 mixture of the two β-epimers of GS-βVG (*i.e.* primarily GS-β(*R*)VG) and the above mentioned alkene impurity (Fig. 8*D*). We found that the abundance of the impurities and the level of the minor isomers in the β-etherase reaction products increased with time after isolation from enzymatic reactions, suggesting that non-enzymatic enolization and dehydration of GS-βVG are responsible for epimerization and side product formation. Thus, we conclude that LigE and LigP each stereoselectively produces GS-β(*S*)VG from β(*R*)GVG, whereas LigF stereoselectively yields GS-β(*R*)VG from β(*S*)GVG.

##### β-S-Thioetherase Activity of LigG with GS-βVG

We also tested for the ability of LigG to catalyze GSH-dependent β-*S*-thioetherase activity in the presence of chemically synthesized GS-βVG (1:1 mixture of GS-β(*S*)VG and GS-β(*R*)VG). We found that the β-*S*-thioether cleavage activity of LigG was insufficient for complete degradation of the synthetic GS-βVG. After chromatographic separation of the LigG assay substrates and reaction products, the ^1^H NMR spectra of the residual GS-βVG showed that LigG had catalyzed β-*S*-thioether cleavage with high stereospecificity toward GS-β(*R*)VG compared with GS-β(*S*)VG. Integration of the ^1^H NMR spectral peaks in the residual GS-βVG (Fig. 8*E*) revealed that GS-β(*S*)VG was at a 5:1 molar excess over the other diastereomer, GS-β(*R*)VG. We also found that LigG-catalyzed degradation of the substrate resulted in the production of β-deoxy-α-veratrylglycerone (see supplemental material), the expected monoaromatic product of GS-β(*R*)VG degradation (Fig. 1*B*). These observations confirm that LigG is a stereospecific β-thioetherase that catalyzes GSH-dependent cleavage of GS-β(*R*)VG, which arises as an intermediate in the β-etherase pathway.

##### β-Etherases Cause Inversion of Chiral Carbon β

Although our data indicated that both GS-β(*S*)VG and GS-β(*R*)VG are intermediates in the β-etherase pathway (Fig. 8, *A–E*), the absolute configurations of the two GS-βVG β-epimers remained unknown. Thus, to assign absolute orientation of the chiral centers at position β in GS-β(*S*)VG and GS-β(*R*)VG, two closely related models with known configurations at position β were synthesized (Fig. 4). The parallel schemes used for β(*S*)-configured (Fig. 4*B*) and β(*R*)-configured (Fig. 4*C*) γ,γ-dimethoxy-β-*S*-(methyl *N*-acetyl cysteinyl)-α-veratrylpropanone (CS-β(*S*)VP and CS-β(*R*)VP) syntheses yielded high purity products with only trace amounts of the undesired β-epimer in each case, as indicated by the ^1^H NMR spectra of CS-β(*S*)VP (Fig. 8*F*) and CS-β(*R*)VP (Fig. 8*G*). Analysis of the ^1^H NMR spectra also revealed that the chemical shifts of protons at β, 2′, and 3′ were affected by the chiral configuration at carbon β, with a pronounced effect observed for the splitting of the two protons at carbon 3′.

The features of these synthetic compounds allowed us to compare them with the products of Lig etherase activity. The shifts of the 3′H_a_ and 3′H_b_ regions in CS-β(*S*)VP (Fig. 8*F*) and CS-β(*R*)VP (Fig. 8*G*) each aligned exclusively with one of the two β-epimers of GS-βVG (Fig. 8, *A–E*). Thus, we propose that the alignment of the CS-β(*S*)VP (Fig. 8*F*) and GS-β(*S*)VG (Fig. 8, *B*, *C*, and *E*) 3′H_a_ and 3′H_b_ spectral regions (and likewise of the CS-β(*R*)VP (Fig. 8*G*) and GS-β(*R*)VG (Fig. 8*D*) alignment) is attributable to the chiral configuration at carbon β. From these results, we conclude that β-etherase catalysis by LigE and LigP causes stereochemical inversion of chiral carbon β from a β(*R*)-substrate to product GS-β(*S*)VG (Fig. 1*A*), whereas LigF carries out inversion of the β(*S*)-substrate chirality in forming product GS-β(*R*)VG (Fig. 1*B*).

## DISCUSSION

The properties of GST family members and many other GSH-dependent enzymes have been well studied due to their important roles in crucial cellular processes (1–4). However, much less is known about the role of GSH and the large number of bacterial GST proteins implicated in catabolic pathways. Our work provides several new insights into the properties of GST family members (LigE, LigF, and LigP) and a GSH-dependent thioetherase (LigG) in the degradation of oligomeric aromatic compounds. The results also provide direct support for the notion that the organism has evolved enzymes to independently deal with both the *R*- and *S*-configured centers in the racemic natural plant lignins (21, 22).

Proteins in the GST family typically use the GSH thiol to initiate a nucleophilic attack on the substrate. We showed that the GST family members LigE, LigF, and LigP each produce a product in which the β-carbon of the substrate is covalently linked to the GSH thiol. We also provided the first experimental evidence that each of these three GST family members have stereospecific and stereoselective β-etherase activity. Our data demonstrate that nucleophilic attack by the GSH thiol on the β-carbon of the substrate is responsible for the β-ether bond cleavage in βGVG and the release of an aromatic monomer (in our case guaiacol) and a second thiol-linked GSH-conjugated monoaromatic product.

Although LigE, LigF, and LigP are each active with β-ether-linked substrates, they are somewhat unusual GST family members because they are stereospecific for the configuration at the β-position of the substrate. We showed that with racemic β-aryl ether-linked model substrates, LigE and LigP each cleave only the β(*R*)-enantiomer, and LigF cleaves only the β(*S*)-stereoisomer. We also found that LigE- and LigP-catalyzed β-etherase reactions exhibit stereoselectivity for the β(*S*)-configured GSH conjugate, whereas LigF yields the β(*R*)-diastereomer. Although the β-etherase catalytic reaction mechanism remains unknown, our findings reveal that β-etherase catalysis causes S_N_2-like inversion of the chiral configuration at carbon β, where β-ether cleavage and β-thioether formation are carried out on opposite faces of the molecule. In sum, our data indicate that these GST family members are both substrate-stereospecific and product-selective.

We also showed that LigG cleaves the GSH conjugates that are produced by β-etherase activity. LigG-mediated cleavage of these GSH conjugates requires the addition of GSH, suggesting that thiol-mediated substrate reduction is needed for this β-*S*-thioetherase activity. Our data therefore provide direct experimental support for the contention that additional activities are not needed to release the other aromatic product of the β-etherase pathway. They also predict that the overall pathway uses GSH to derivatize and release one aromatic product (in a reaction catalyzed by LigE, LigF, or LigP) prior to reductive cleavage and release of the second aromatic (by LigG). Overall, this strategy is reminiscent of the chemical “derivatization followed by reductive cleavage” (DFRC) method used to release aromatic monomers from lignin (48, 49).

Our data show that β-etherase catalysis by the GST family members LigE, LigP, and LigF is achieved through stereospecific β-ether bond cleavage, inversion of the chiral (*R*/*S*)-configuration at carbon β, and concomitant β-*S*-thioether bond formation. We have also unambiguously identified GSH conjugates as the previously uncharacterized glutathionyl-*S*-thioether-linked intermediates of the β-etherase pathway in *Sphingobium* sp. strain SYK-6. Further, given that (*a*) LigE/LigP and LigF stereoselectively produce GS-β(*S*)VG and GS-β(*R*)VG (respectively), (*b*) LigG converts the LigF β-etherase product GS-β(*R*)VG to β-deoxy-α-veratrylglycerone, and (*c*) LigG has little or no activity as a β-thioetherase with the LigE/LigP-produced GS-β(*S*)VG, we conclude that LigG is a stereospecific β-*S*-thioetherase that plays a role in the derivation of monoaromatic compounds in the β-etherase pathway. The fate of the LigE/LigP-produced GSH-conjugated β-etherase pathway intermediate, GS-β(*S*)VG, currently remains unknown. Assumedly, cells either use racemase-like enzymes for the conversion of the inactive β-epimer to the GSH conjugate that is cleaved by LigG, possess a second β-*S*-thioetherase with the required stereospecificity for GSH-dependent cleavage of the β(*S*)-isomer, or employ other metabolic activities that enable the bacterium to utilize GS-β(*S*)VG as a growth substrate.

In sum, we have provided new insights into the enzymes, substrates, and products of a novel catabolic β-etherase pathway (14–20) that has recently been garnering considerable attention. We found a remarkably high degree of stereospecificity and stereoselectivity for these GSH-dependent enzymes. We propose that the existence of multiple GST family member β-etherases with complementary stereochemical features can be rationalized by the combinatorial radical chemistry that is used to synthesize plant lignins and the resulting racemic nature of the β-ether (and other) subunits of this polymer. The existence of β(*R*)- and β(*S*)-configurations in native plant lignins necessitates both β(*R*)- and β(*S*)-stereospecific β-etherases and β-thioetherases. Although these enzymes could cleave other structurally related compounds, the stereochemical features of the enzymes described support a hypothesis that they normally function in a pathway that processes racemic substrates similar to those found in nature. These defined properties of the GSH-dependent enzymes could help in producing valuable chiral products from individual stereoisomers of β-aryl substrates that might be derived from lignin degradation.

## Supplementary Material

Supplemental Data

## Footnotes

2 The abbreviations used are:

βGVG: β-guaiacyl-α-veratrylglycerone
β(*S*)GVG: β(*S*)-guaiacyl-α-veratrylglycerone
β(*R*)GVG: β(*R*)-guaiacyl-α-veratrylglycerone
GS-βVG: β-*S*-glutathionyl-α-veratrylglycerone
GS-β(*S*)VG: β(*S*)-*S*-glutathionyl-α-veratrylglycerone
GS-β(*R*)VG: β(*R*)-*S*-glutathionyl-α-veratrylglycerone
βGVE: β-guaiacyl-α-veratrylethanone
GS-βVE: β-*S*-glutathionyl-α-veratrylethanone
CS-βVP: γ,γ-dimethoxy-β-*S*-(methyl *N*-acetyl cysteinyl)-α-veratrylpropanone
CS-β(*S*)VP: γ,γ-dimethoxy-β(*S*)-*S*-(methyl *N*-acetyl-cysteinyl)-α-veratrylpropanone
CS-β(*R*)VP: γ,γ-dimethoxy-β(*R*)-*S*-(methyl *N*-acetyl-cysteinyl)-α-veratrylpropanone
NHis_8_: N-terminal octahistidine
Ni-NTA: nickel-nitrilotriacetic acid
TCEP: *tris*-(2-carboxyethyl)-phosphine hydrochloride
*t_R_*: retention time
HSQC: (^1^H-^13^C) heteronuclear single-quantum coherence (NMR spectroscopy)
HMBC: (^1^H-^13^C) heteronuclear multiple-bond correlation (NMR spectroscopy)
COSY: (^1^H-^1^H) correlation spectroscopy
Tev: tobacco etch virus.
