# Supplementary material for: Stereochemical Features of Glutathione-dependent Enzymes in the Sphingobium sp. Strain SYK-6 β-Aryl Etherase Pathway
Source: J Biol Chem. 2014 Feb 7;289(12):8656–67. doi: 10.1074/jbc.M113.536250 (PMC3961688; doi:10.1074/jbc.M113.536250)
Supplement: Supplemental Data [file supp_289_12_8656__index.html]

Stereochemical features of glutathione-dependent enzymes in the Sphingobium sp. strain SYK-6 β-aryl etherase pathway — Stereochemical Features of Glutathione-dependent Enzymes in the Sphingobium sp. Strain SYK-6 β-Aryl Etherase Pathway — Stereochemical Features of β-Etherase Pathway Enzymes — Supplemental Data 

# Stereochemical Features of Glutathione-dependent Enzymes in the *Sphingobium* sp. Strain SYK-6 β-Aryl Etherase Pathway

## Supplemental Data

**Files in this Data Supplement:**

- Supplementary Information (.pdf, 3.9 MB) - contains details on organic synthesis and NMR data
