# Supplementary material for: Stereochemical Features of Glutathione-dependent Enzymes in the Sphingobium sp. Strain SYK-6 β-Aryl Etherase Pathway
Source: J Biol Chem. 2014 Feb 7;289(12):8656–67. doi: 10.1074/jbc.M113.536250 (PMC3961688; doi:10.1074/jbc.M113.536250)

Stereochemical features of glutathione-dependent enzymes in the *Sphingobium sp.* strain SYK-6 β-aryl etherase pathway

Daniel L. Gall,[1,2] Hoon Kim,[2,3] Fachuang Lu,[2,3] Timothy J. Donohue,[2,4] Daniel R. Noguera,[1,2,5] and John Ralph[2,3]*

[1]Department of Civil & Environmental Engineering, University of Wisconsin, Madison, WI 53706

[2]U.S. Department of Energy Great Lakes Bioenergy Research Center, Wisconsin Energy Institute, University of Wisconsin, Madison WI 53726

[3]Department of Biochemistry, University of Wisconsin, Madison, WI 53706

[4]Department of Bacteriology, University of Wisconsin, Madison, WI 53706

[5]Environmental Chemistry and Technology Program, University of Wisconsin, Madison WI 53706

SUPPLEMENTARY INFORMATION – SYNTHETIC DETAILS AND NMR DATA

CHEMICAL SYNTHESES:

General. β-Ether-linked model compounds were synthesized according to the method of Adler and Eriksoo (1, 2). R_f values were calculated as the ratio of elution volume to total solvent volume (v/v). Chromatographic elution volumes were calculated as the product of the mobile phase flow rate (in mL min^{-1}) and a given compound's retention time (t_R, in min).

Chemicals and reagents were purchased from Sigma-Aldrich. ^1H and ^{13}C NMR spectra were recorded on a Bruker Biospin (Billerica, MA) AVANCE 700 MHz spectrometer fitted with a cryogenically cooled 5-mm TXI gradient probe with inverse geometry (proton coils closest to the sample). Chemical shifts are reported in parts per million (ppm). The central NMR solvent peaks were used as internal references (δ_H: 2.05 ppm and δ_C: 29.8 ppm for acetone-d_6; δ_H: 4.79 ppm for (HDO in) D_2O (3, 4). J values are recorded in Hz. Carbon and proton assignments for all compounds as labeled in the included ^1H and ^{13}C NMR spectra were determined by via the aid of 2D COSY, HSQC, and HMBC NMR spectra. Merck-EMD Millipore aluminum-backed Silica Gel 60 F_{254} normal-phase thin-layer chromatography plates were used for small-scale separation of organic compounds using a mixture of hexane and ethyl acetate as the mobile solvent. Biotage KP-Sil silica gel was used for preparative separations of organic compounds by flash chromatography using a CombiFlash R_f delivery module using a mixture of hexane and ethyl acetate as the mobile phase.

Synthesis of β-bromo-α-veratrylethanone (Fig. 3). A solution of ethyl acetate (200 mL), compound α-veratrylethanone (7.24 g, 40.2 mmol), and pyridinium tribromide (13.5 g, 42.2 mmol) was prepared in a 500-mL round-bottom flask with stirring. After 30 min, the reaction mixture was washed three times with saturated Na_2CO_3, once with H_2O, and once with brine. The organic layer was then dried over $MgSO_4$ and the solvent was evaporated *in vacuo*. The resulting residue was then dissolved in hot methanol and allowed to cool, affording crystalline β-bromo-α-veratrylethanone (6.1 g, 59% yield).

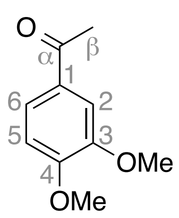

α-Veratrylethanone [from Sigma-Aldrich]:
^{1}H NMR (700 MHz, acetone-d_6) δ 7.61 (dd, 1H, *J* = 8.4, 2.1 Hz, H6); 7.49 (d, 1H, *J* = 2.1 Hz, H2); 7.01 (d, 1H, *J* = 8.4 Hz, H5); 3.88 (s, 3H, 4-OMe); 3.86 (s, 3H, 3-OMe); 2.51 (s, 3H, $H\beta_{a/b/c}$).
^{13}C NMR (176 MHz, acetone-d_6) δ 196.4 (Cα); 154.4 (C4); 150.0 (C3); 131.2 (C1); 123.8 (C6); 111.3 (C5); 111.1 (C2); 56.1 (4-OMe); 55.9 (3-OMe); 26.3 (Cβ).

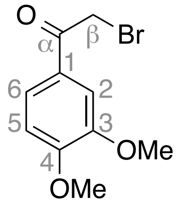

β-Bromo-α-veratrylethanone [synthetic]:
^{1}H NMR (700 MHz, acetone-d_6) δ 7.72 (dd, 1H, *J* = 8.4, 2.1 Hz, H6); 7.55 (d, 1H, *J* = 2.1 Hz, H2); 7.08 (d, 1H, *J* = 8.4 Hz, H5); 4.69 (s, 2H, $H\beta_{a/b}$); 3.92 (s, 3H, 4-OMe); 3.88 (s, 3H, 3-OMe).
^{13}C NMR (176 MHz, acetone-d_6) δ 190.5 (Cα); 155.1 (C4); 150.2 (C3); 127.9 (C1); 124.5 (C6); 111.8 (C5); 111.5 (C2); 56.2 (4-OMe); 56.0 (3-OMe); 32.5 (Cβ).

Synthesis of β-guaiacyl-α-veratrylethanone, βGVE (Fig. 3). To a magnetically stirred solution of acetone (80 mL), β-bromo-α-veratrylethanone (4.0 g, 15.5 mmol), and guaiacol (2.0 g, 16.3 mmol) contained in a 250-mL round-bottom flask, anhydrous K_2CO_3 (4.3 g, 31.0 mmol) was added and the reaction mixture was set to reflux for 4 h. Inorganics were then removed by filtration and the filtrate was evaporated *in vacuo*. The resulting residue was taken up with ethyl acetate and washed twice with aqueous 1 N NaOH, twice with H_2O, and once with brine. The organic layer was then dried over $MgSO_4$ and the solvent was again evaporated *in vacuo*. The residue was then dissolved in hot ethanol and allowed to cool, affording crystalline β-guaiacyl-α-veratrylethanone (3.8 g, 82% yield).

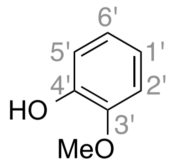

Guaiacol [from Sigma-Aldrich]:
^{1}H NMR (700 MHz, acetone-d_6) δ 7.51 (s, 1H, 4′-OH); 6.93 (dd, 1H, *J* = 7.6, 1.9 Hz, H2′); 6.84 - 6.77 (m, 3H, H1′/H5′/H6′); 3.82 (s, 3H, 3′-OMe).
^{13}C NMR (176 MHz, acetone-d_6) δ 148.3 (C3′); 147.5 (C4′); 121.9 (C6′); 120.4 (C1′ or C5′); 115.8 (C5′ or C1′); 112.4 (C2′); 56.1 (3′-OMe).

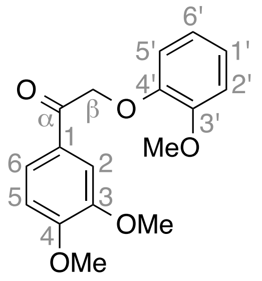

β-guaiacyl-α-veratrylethanone, βGVE [synthetic]:
^{1}H NMR (700 MHz, acetone-d_6) δ 7.76 (dd, 1H, *J* = 8.4, 2.0 Hz, H6); 7.60 (d, 1H, *J* = 2.0 Hz, H2); 7.07 (d, 1H, *J* = 8.4 Hz, H5); 6.98 (dd, 1H, *J* = 8.4, 1.5 Hz, H2′); 6.93 – 6.90 (m, 2H, H1′/H5′); 6.83 (dt, 1H, *J* = 7.7, 1.5 Hz, H6′); 5.37 (s, 2H, $H\beta_{a/b}$); 3.91 (s, 3H, 4-OMe); 3.88 (s, 3H, 3-OMe); 3.83 (s, 3H, 3′-OMe).
^{13}C NMR (176 MHz, acetone-d_6) δ 193.7 (Cα); 154.9 (C4); 150.7 (C3′); 150.2 (C3); 149.1 (C4′); 128.8 (C1); 123.5 (C6); 122.6 (C1′); 121.5 (C6′); 115.3 (C5′); 113.5 (C2′); 111.5 (C5); 111.3 (C2); 72.2 (Cβ); 56.2 (4-OMe); 56.1 (3′-OMe); 56.0 (3-OMe).

Synthesis of racemic β-guaiacyl-α-veratrylglycerone, βGVG. To a magnetically stirred solution of 1,4-dioxane (60 mL), βGVE (3.4 g, 11.5 mmol), and formaldehyde (0.354 g, 11.8 mmol, 0.9 mL of 37% formaldehyde in H_2O) in a 250-mL round-bottom flask, anhydrous K_2CO_3 (3.2 g, 23.1 mmol) was added and the reaction mixture was set to 40 °C. After 3 h, the reaction was cooled to room temperature, carbonates were removed by filtration, and 1,4-dioxane was evaporated *in vacuo*. The residue was

dissolved in ethyl acetate and washed three times with H_2O and once with brine. The organic layer was then dried over $MgSO_4$ and the solvent evaporated *in vacuo*. Racemic βGVG was then crystallized from ethyl acetate and hexane (3.4 g, 88% yield). β(*S*)GVG and β(*R*)GVG were then separated and purified by preparative chiral chromatography as described in the main text.

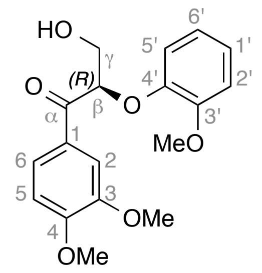

β(*R*)-guaiacyl-α-veratrylglycerone, β(*R*)GVG [synthetic]:
^{1}H NMR (700 MHz, acetone-d_6) δ 7.84 (dd, 1H, *J* = 8.4, 2.0 Hz, H6); 7.65 (d, 1H, *J* = 2.0 Hz, H2); 7.05 (d, 1H, *J* = 8.4 Hz, H5); 6.97 (dd, 1H, *J* = 8.1, 1.4 Hz, H2´); 6.90 (dt, 1H, *J* = 7.9, 1.4 Hz, H1´); 6.86 (dd, 1H, *J* = 8.1, 1.4 Hz, H5´); 6.78 (dt, 1H, *J* = 7.9, 1.4 Hz, H6´); 5.54 (t, 1H, *J* = 5.2 Hz, Hβ); 4.26 (t, 1H, *J* = 6.3 Hz, γ-OH); 4.07 – 4.05 (m, 2H, $H\gamma_{a/b}$); 3.89 (s, 3H, 4-OMe); 3.85 (s, 3H, 3-OMe); 3.79 (s, 3H, 3´-OMe).
^{13}C NMR (176 MHz, acetone-d_6) δ 195.9 (Cα); 154.8 (C4); 150.9 (C3´); 150.0 (C3); 148.4 (C4´); 129.4 (C1); 124.2 (C6); 123.0 (C1´); 121.5 (C6´); 116.8 (C5´); 113.6 (C2´); 112.0 (C2); 111.4 (C5); 83.9 (Cβ); 64.1 (Cγ); 56.1 (4-OMe); 56.1 (3´-OMe); 55.9 (3-OMe).
HPLC (Diacel CHIRALPAK AY-H, Hexane/EtOH = 1/1, flow rate = 2.5 mL min^{-1}) t_R = 80.6 min, R_f = 0.10.

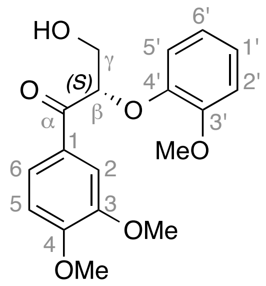

β(*S*)-guaiacyl-α-veratrylglycerone, β(*S*)GVG [synthetic]:
^{1}H NMR (700 MHz, acetone-d_6) δ 7.84 (dd, 1H, *J* = 8.4, 2.0 Hz, H6); 7.65 (d, 1H, *J* = 2.0 Hz, H2); 7.05 (d, 1H, *J* = 8.4 Hz, H5); 6.97 (dd, 1H, *J* = 8.1, 1.4 Hz, H2´); 6.90 (dt, 1H, *J* = 7.9, 1.4 Hz, H1´); 6.86 (dd, 1H, *J* = 8.1, 1.4 Hz, H5´); 6.78 (dt, 1H, *J* = 7.9, 1.4 Hz, H6´); 5.54 (t, 1H, *J* = 5.2 Hz, Hβ); 4.26 (t, 1H, *J* = 6.3 Hz, γ-OH); 4.07 – 4.05 (m, 2H, $H\gamma_{a/b}$); 3.89 (s, 3H, 4-OMe); 3.85 (s, 3H, 3-OMe); 3.79 (s, 3H, 3´-OMe).
^{13}C NMR (176 MHz, acetone-d_6) δ 195.9 (Cα); 154.8 (C4); 150.9 (C3´); 150.0 (C3); 148.4 (C4´); 129.4 (C1); 124.2 (C6); 123.0 (C1´); 121.5 (C6´); 116.8 (C5´); 113.6 (C2´); 112.0 (C2); 111.4 (C5); 83.9 (Cβ); 64.1 (Cγ); 56.1 (4-OMe); 56.1 (3´-OMe); 55.9 (3-OMe).
HPLC (Diacel CHIRALPAK AY-H, Hexane/EtOH = 1/1, flow rate = 2.5 mL min^{-1}) t_R = 21.8 min, R_f = 0.47.

Synthesis of GS-βVE (Fig. 3). Bromide β-bromo-α-veratrylethanone (518 mg, 2.0 mmol) was dissolved in acetone (10 mL) and stirred in a 50-mL round-bottom flask. Glutathione (615 mg, 2.0 mmol) and $NaHCO_3$ (504 mg, 6.0 mmol) were dissolved in H_2O (10 mL) and the aqueous solution was added to the acetone-bromide solution. The reaction was incubated at room temperature overnight (approximately 14 h), after which time, the reaction was diluted with CH_2Cl_2 (10 mL), forcing GS-βVE into the aqueous layer. The aqueous fraction was washed three more times with CH_2Cl_2 and then dried *in vacuo*. GS-βVE was then further purified by C_{18}-reversed phase chromatography. The residue of GS-βVE was dissolved in 0.5 mL of H_2O and injected into a pre-packed Biotage KP-C_{18} (100 g) reversed phase column using a mixture of water and methanol as the mobile phase at a flow rate of 10 mL/min. The ratio of buffers was adjusted as follows: 0–15 min, 0% methanol; 15–20 min, gradient from 0-100% methanol; 20–35 min, 100% methanol; 35–40 min, gradient from 100-0% methanol; 40–50 min, 0% methanol. The mobile phase was carried through the column using a Beckman 125NM solvent delivery module equipped with a Beckman 168 UV detector. Fractions with high UV absorption at 280 nm were collected and pooled. GS-βVE eluted from the Biotage KP-C18 column with a t_R = 31.0 min.

When D_2O was used as the NMR solvent in the analysis of GS-βVE, partial exchange of the βH_2-protons with deuterium suppressed the $H\beta_{a/b}$ signal in both the ^1H and HSQC NMR spectra of GS-βVE. From the correlation between $7'H_{A/B}$ and carbon-β, however, the HMBC spectra revealed that the β-carbon signal was at a chemical shift of approximately 38 ppm. A correlation was also observed between carbon-7′ and the $H\beta_{a/b}$ protons, confirming that the weaker-than-expected signal observed at 3.9 ppm in the ^1H NMR spectra was in fact from βH_2-protons. However, an accurate integration of the $H\beta_{a/b}$ peak could not be obtained for GS-βVE in D_2O as a result of deuterium exchange. To obtain an accurate integration of this moiety, compound GS-βVE was also analyzed with water suppression in 90% H_2O/10% D_2O NMR solvent, enabling identification of all expected protons and carbons.

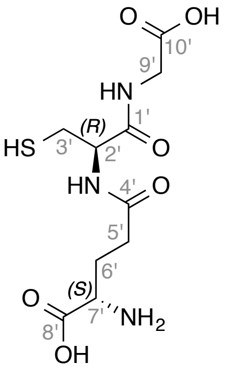

2′(*R*),7′(*S*)-Glutathione, GSH [from Sigma-Aldrich]:
^1H NMR (700 MHz, D_2O) δ 4.43 (dd, 1H, J = 6.6, 5.5 Hz, H2′); 3.84 (d, 2H, J = 1.8 Hz, $H9'_{a/b}$); 3.69 (t, 1H, J = 6.4 Hz, H7′); 2.83 (d, 1H, J = 5.5 Hz, $H3'_a$); 2.82 (d, 1H, J = 6.6 Hz, $H3'_b$); 2.43 (t, 2H, J = 7.3 Hz, $H5'_{a/b}$); 2.05 (dt, 2H, J = 7.3, 6.4 Hz, $H6'_{a/b}$).
^{13}C NMR (176 MHz, D_2O) δ 175.6 (C4′); 174.3 (C10′); 174.3 (C8′); 173.1 (C1′); 56.4 (C2′); 54.4 (C7′); 42.3 (C9′); 31.9 (C5′); 26.7 (C6′); 26.1 (C3′).

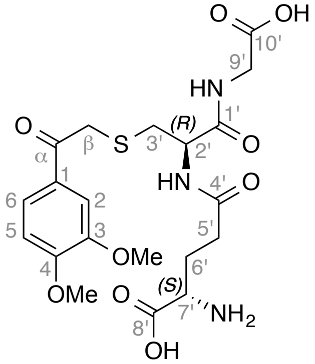

β-S-glutathionyl-α-veratrylethanone, GS-βVE [synthetic]:
^1H NMR (700 MHz, D_2O) δ 7.61 (dd, 1H, J = 8.4, 2.0 Hz, H6); 7.43 (d, 1H, J = 2.0 Hz, H2); 7.00 (d, 1H, J = 8.4 Hz, H5); 4.43 (dd, 1H, J = 9.1, 4.9 Hz, H2′); 3.82 (s, 3H, 4-OMe); 3.79 (s, 3H, 3-OMe); 3.62 (d, 1H, J = 17.3 Hz, $H9'_a$); 3.58 (t, 1H, J = 6.4 Hz, H7′); 3.56 (d, 1H, J = 17.3 Hz, $H9'_b$); 2.98 (dd, 1H, J = 14.3, 4.9 Hz, $H3'_a$); 2.78 (dd, 1H, J = 14.3, 9.1 Hz, $H3'_b$); 2.33 (t, 2H, J = 7.8 Hz, $H5'_{a/b}$); 1.96 (dt, J = 7.8, 6.4 Hz, $H6'_{a/b}$).
^{13}C NMR (176 MHz, D_2O) δ 197.6 (Cα); 176.9 (C10′); 175.6 (C4′); 174.7 (C8′); 172.4 (C1′); 154.1 (C4); 148.7 (C3); 128.3 (C1); 125.3 (C6); 111.5 (C5); 111.2 (C2); 56.6 (4-OMe); 56.3 (3-OMe); 54.8 (C7′); 53.6 (C2′); 44.1 (C9′); 38.4 (Cβ); 34.1 (C3′); 32.1 (C5′); 27.0 (C6′).

^1H NMR (700 MHz, 90% H_2O/10% D_2O) δ 7.53 (dd, 1H, J = 8.9, 1.7 Hz, H6); 7.32 (d, 1H, J = 1.7 Hz, H2); 6.92 (d, 1H, J = 8.9 Hz, H5); 4.43 (dd, 1H, J = 9.4, 4.9 Hz, H2′); 3.92 (s, 2H, $H\beta_{a/b}$); 3.79 (s, 3H, 4-OMe); 3.74 (s, 3H, 3-OMe); 3.62 (d, 1H, J = 17.3 Hz, $H9'_a$); 3.58 (t, 1H, J = 6.7 Hz, H7′); 3.56 (d, 1H, J = 17.3 Hz, $H9'_b$); 2.96 (dd, 1H, J = 14.6, 4.9 Hz, $H3'_a$); 2.77 (dd, 1H, J = 14.6, 9.4 Hz, $H3'_b$); 2.33 (t, 2H, J = 8.0 Hz, $H5'_{a/b}$); 1.96 (dt, J = 8.0, 6.7 Hz, $H6'_{a/b}$).
^{13}C NMR (176 MHz, 90% H_2O/10% D_2O) δ 198.1 (Cα); 176.9 (C10′); 175.7 (C4′); 175.1 (C8′); 172.5 (C1′); 154.2 (C4); 148.8 (C3); 128.5 (C1); 125.4 (C6); 111.6 (C5); 111.5 (C2); 56.7 (4-OMe); 56.4 (3-OMe); 55.1 (C7′); 53.6 (C2′); 44.2 (C9′); 38.4 (Cβ); 34.1 (C3′); 32.2 C5′); 27.3 (C6′).

Synthesis of Ethyl α-keto-veratrylpropionate (Fig. 4). A refluxing mixture of tetrahydrofuran (125 mL), diethyl carbonate (12.73 g, 107.8 mmol), and sodium hydride (2.9 g, 122.4 mmol, 4.9 g of 60% dispersion in mineral oil) was prepared in a 3-necked 250-mL round-bottom flask equipped with a 50-mL cylindrical addition funnel. A solution of α-veratrylethanone (8.8 g, 49.0 mmol) dissolved in tetrahydrofuran (50 mL) was added dropwise to the refluxing mixture over the course of 60 min and the reaction was allowed to proceed for an additional 60 min before it was cooled to room temperature.

Excess sodium hydride was then quenched with the dropwise addition of anhydrous ethanol (10 mL) and saturated NH_4Cl (10 mL). Solvents were then evaporated *in vacuo* and the resulting oil was dissolved in ethyl acetate and washed twice with saturated NH_4Cl (10 mL), twice with H_2O, and once with brine. The organic layer was dried over $MgSO_4$ and solvent again evaporated *in vacuo*. β-keto ester Ethyl α-keto-veratrylpropionate was then crystallized from ethyl acetate and hexane (10.8 g, 88% yield).

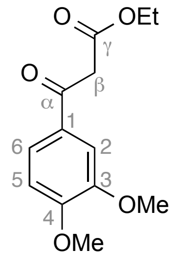

Ethyl α-keto-veratrylpropionate [synthetic]:
^{1}H NMR (700 MHz, acetone-d_6) δ 7.65 (dd, 1H, J = 8.4, 2.1 Hz, H6); 7.52 (d, 1H, J = 2.1 Hz, H2); 7.06 (d, 1H, J = 8.4 Hz, H5); 4.15 (q, 2H, J = 7.1 Hz, Et-methylene$_{a/b}$); 4.04 (s, 2H, H$\beta_{a/b}$); 3.91 (s, 3H, 4-OMe); 3.88 (s, 3H, 3-OMe); 1.21 (t, 3H, J = 7.1 Hz, Et-methyl$_{a/b/c}$).
^{13}C NMR (176 MHz, acetone-d_6) δ 192.0 (Cα); 168.6 (Cγ); 155.0 (C4); 150.2 (C3); 130.2 (C1); 124.2 (C6); 111.5 (C5); 111.3 (C2); 61.4 (Et-methylene); 56.2 (4-OMe); 56.0 (3-OMe); 46.1 (Cβ); 14.5 (Et-methyl).

Synthesis of β-deoxy-α-veratrylglycerol (Fig. 4). To a magnetically stirred solution of Ethyl α-keto-veratrylpropionate (4.0 g, 15.9 mmol) dissolved in tetrahydrofuran (45 mL) in a 250-mL round-bottom flask maintained under an inert atmosphere at 0 °C, diisobutylaluminum hydride (DIBAL-H, 7.8 g, 55.0 mmol, 55 mL of 1.0 M DIBAL-H solution in tetrahydrofuran) was added dropwise. After 2 h, anhydrous ethanol (5 mL) was added dropwise over a period of 10 min to quench the remaining DIBAL-H. A saturated aqueous solution of Rochelle salt (potassium sodium tartrate, 40 mL) was then added dropwise over the course of 20 min and the mixture was allowed to mix for an additional 2 h in order to hydrolyze DIBAL adducts. H_2O (50 mL) and ethyl acetate (30 mL) was then added to the mixture and the organic layer was collected. Five additional ethyl acetate extractions were applied to the aqueous layer and the organic fractions were pooled. The solvents were then evaporated *in vacuo*, affording β-deoxy-α-veratrylglycerol as an oil (3.1 g, 91% yield).

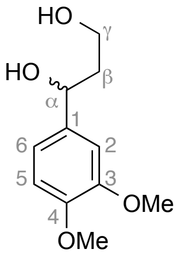

β-Deoxy-α-veratrylglycerol [synthetic]:
^{1}H NMR (700 MHz, acetone-d_6) δ 6.99 (brs, 1H, H2); 6.87 (m, 2H, H5/H6); 4.81 (q, 1H, J = 4.1 Hz, Hα); 4.32 (d, 1H, J = 4.1 Hz, α-OH); 3.79 (s, 3H, 4-OMe); 3.77 (s, 3H, 3-OMe); 3.75 (t, 1H, J = 5.0 Hz, γ-OH); 3.74 – 3.70 (m, 1H, Hγ_a); 3.68 – 3.64 (m, 1H, Hγ_b); 1.92 – 1.87 (m, 1H, Hβ_a); 1.84 – 1.80 (m, 1H, Hβ_b)
^{13}C NMR (176 MHz, acetone-d_6) δ 150.2 (C4); 149.2 (C3); 139.7 (C1); 118.6 (C2); 112.4 (C5); 110.6 (C6); 72.4 (Cα); 60.4 (Cγ); 56.1 (4-OMe); 55.9 (3-OMe); 43.0 (Cβ).

Synthesis of β-deoxy-α-veratrylglycerone (Fig. 4). A solution of β-deoxy-α-veratrylglycerol (2.5 g, 11.8 mmol) dissolved in 1,4-dioxane (30 mL) was prepared in a 250-mL round-bottom flask. 2,3-dichloro-5,6-dicyano-1,4-benzoquinone (DDQ, 2.9 g, 13.0 mmol) was added to the solution and the contents were stirred at room temperature for 30 min. The reaction mixture was then filtered for the removal of DDQ-H. The solvent was then evaporated *in vacuo* and compound β-deoxy-α-veratrylglycerone (1.8 g, 74% yield, R_f = 0.77) was purified from the resulting residue by flash chromatography using Hexane/EtOAc = 1/3. Compound β-deoxy-α-veratrylglycerone was then crystallized from ethyl acetate and hexane.

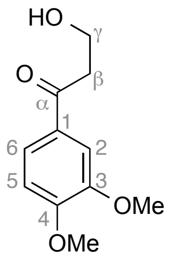

β-Deoxy-α-veratrylglycerone [synthetic]:
^{1}H NMR (700 MHz, acetone-d_6) δ 7.67 (d, 1H, J = 8.4, 2.0 Hz, H6); 7.53 (d, 1H, J = 2.0 Hz, H2); 7.05 (d, 1H, J = 8.4 Hz, H5); 3.93 – 3.90 (m, 2H, $H\gamma_{a/b}$); 3.90 (s, 3H, 4-OMe); 3.87 (s, 3H, 3-OMe); 3.60 (t, 1H, J = 5.7 Hz, γ-OH); 3.16 (d, 2H, J = 6.2 Hz, $H\beta_{a/b}$)
^{13}C NMR (176 MHz, acetone-d_6) δ 198.2 (Cα); 154.5 (C4); 150.1 (C3); 131.3 (C1); 123.5 (C6); 111.4 (C5); 111.1 (C2); 58.6 (Cγ); 56.1 (4-OMe); 56.0 (3-OMe); 41.5 (Cβ).

Synthesis of β-bromo-α-veratrylglycerone (Fig. 4). To a magnetically stirred solution of β-deoxy-α-veratrylglycerone (0.7 g, 3.3 mmol) dissolved in ethyl acetate (20 mL) that was prepared in a 50-mL round-bottom flask, pyridinium tribromide (1.1 g, 3.5 mmol) was added. The reaction was allowed to mix at room temperature for 30 min and was then washed three times with saturated Na_2CO_3, once with H_2O, and once with brine. The organic layer was then dried over $MgSO_4$ and the residue obtained after *in vacuo* evaporation of solvent and products were purified by flash chromatography using a mobile phase of Hexane/EtOAc = 3/1. Collection of the chromatography fractions afforded both a β,γ-epoxide (0.5 g, 73% yield, R_f = 0.68) and the desired bromide β-bromo-α-veratrylglycerone (0.3 g, 27% yield, R_f = 0.22) which was crystallized from ethyl acetate and hexane.

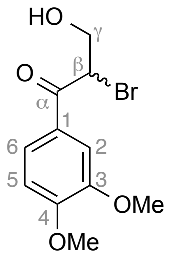

β-Bromo-α-veratrylglycerone [synthetic]:
^{1}H NMR (700 MHz, acetone-d_6) δ 7.78 (dd, 1H, J = 8.5, 2.1 Hz, H6); 7.58 (d, 1H, J = 2.1 Hz, H2); 7.10 (d, 1H, J = 8.5 Hz, H5); 5.46 (dd, 1H, J = 7.8, 5.7 Hz, Hβ); 4.43 (t, 1H, J = 6.2 Hz, γ-OH); 4.20 (dd, 1H, J = 11.3, 7.8 Hz, $H\gamma_a$); 3.93 (dd, 1H, J = 11.3, 5.7 Hz, $H\gamma_b$); 3.93 (s, 3H, 4-OMe); 3.89 (s, 3H, 3-OMe).
^{13}C NMR (176 MHz, acetone-d_6) δ 192.3 (Cα); 155.2 (C4); 150.3 (C3); 128.6 (C1); 124.5 (C6); 111.8 (C2); 111.5 (C5); 63.5 (Cγ); 56.3 (4-OMe); 56.1 (3-OMe); 46.1 (Cβ).

Synthesis of β-S-glutathionyl-α-veratrylglycerone, GS-βVG (Fig. 4). Bromide β-bromo-α-veratrylglycerone (144 mg, 0.5 mmol) was dissolved in acetone (10 mL) and stirred in a 50-mL round-bottom flask. Glutathione (154 mg, 0.5 mmol) and $NaHCO_3$ (126 mg, 1.5 mmol) were dissolved in H_2O (10 mL) and the aqueous solution was added to the acetone-bromide solution. The reaction was incubated at room temperature overnight (approximately 14 h), after which time, the reaction was diluted with CH_2Cl_2 (10 mL), forcing the synthetic mixture of GS-β(*S*)VG and GS-β(*R*)VG into the aqueous layer. The aqueous fraction was washed three more times with CH_2Cl_2 and then dried *in vacuo*. The diastereomeric mixture of GS-β(*S*)VG and GS-β(*R*)VG was then further purified by C_{18}-reversed phase chromatography. The residue of GS-βVG was dissolved in 0.5 mL of H_2O and injected into a pre-packed Biotage KP-C_{18} (100 g) reversed phase column using a mixture of water and methanol as the mobile phase at a flow rate of 10 mL/min. The ratio of buffers was adjusted as follows: 0–15 min, 0% methanol; 15–20 min, gradient from 0-100% methanol; 20–35 min, 100% methanol; 35–40 min, gradient from 100-0% methanol; 40–50 min, 0% methanol. The mobile phase was carried through the column using a Beckman 125NM solvent delivery module equipped with a Beckman 168 UV detector. Fractions with high UV absorption at 280 nm were collected and pooled. The mixture of the two diastereomers eluted from the Biotage KP-C18 column with a t_R = 31.0 min.

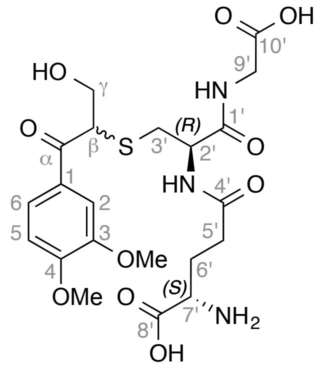

β(*S*)-S-glutathionyl-α-veratrylglycerone, GS-β(*S*)VG and GS-β(*R*)VG [synthetic]:
^{1}H NMR (700 MHz, D_2O) δ 7.68 – 7.66 (m, 2H, ***S***-H6/***R***-H6); 7.47 (d, 2H, *J* = 2.0 Hz, ***S***-H2/***R***-H2); 7.01 (d, 2H, *J* = 8.4 Hz, ***S***-H5/***R***-H5); 4.58 (dd, 1H, *J* = 8.0, 5.9 Hz, ***R***-Hβ); 4.54 (dd, 1H, *J* = 7.9, 5.9 Hz, ***S***-Hβ); 4.35 (dd, 1H, *J* = 9.0, 4.9 Hz, ***R***-H2′); 4.27 (dd, 1H, *J* = 8.8, 5.1 Hz, ***S***-H2′); 3.98 (dd, 1H, *J* = 11.6, 7.9 Hz, ***S***-$H\gamma_a$); 3.95 (dd, 1H, *J* = 11.5, 8.0 Hz, ***R***-$H\gamma_a$); 3.83 (s, 6H, ***S***-4-OMe/***R***-4-OMe); 3.83 - 3.78 (m, 2H, ***S***-$H\gamma_b$/***R***-$H\gamma_b$); 3.80 (s, 6H, ***S***-3-OMe/***R***-3-OMe); 3.58 (d, 1H, *J* = 17.3 Hz, ***R***-$H9'_a$); 3.57 (d, 1H, *J* = 17.3 Hz, ***S***-$H9'_a$); 3.55 (t, 2H, *J* = 6.4 Hz, ***S***-H7′/***R***-H7′); 3.50 (d, 1H, *J* = 17.3 Hz, ***S***-$H9'_b$); 3.49 (d, 1H, *J* = 17.3 Hz, ***R***-$H9'_b$); 3.04 (dd, 1H, *J* = 14.0, 4.9 Hz, ***R***-$H3'_a$); 2.94 (dd, 1H, *J* = 14.2, 5.1 Hz, ***S***-$H3'_a$); 2.77 (dd, 1H, *J* = 14.2, 8.8 Hz, ***S***-$H3'_b$); 2.69 (dd, 1H, *J* = 14.0, 9.0 Hz, ***R***-$H3'_b$); 2.29 – 2.17 (m, 4H, ***S***-$H5'_{a/b}$/***R***-$H5'_{a/b}$); 1.93 – 1.86 (m, 4H, ***S***-$H6'_{a/b}$/***R***-$H6'_{a/b}$).
^{13}C NMR (176 MHz, D_2O) δ 197.4 (***R***-Cα); 197.2 (***S***-Cα); 175.9 (***R***-C10′); 175.9 (***S***-C10′); 175.3 (***R***-C4′); 175.3 (***S***-C4′); 174.7 (***R***-C8′); 174.6 (***S***-C8′); 171.2 (***R***-C1′); 171.1 (***S***-C1′); 153.4 (***R***-C4); 153.4 (***S***-C4); 148.0 (***R***-C3); 147.9 (***S***-C3); 127.9 (***R***-C1); 127.8 (***S***-C1); 124.3 (***R***-C6); 124.2 (***S***-C6); 110.7 (***R***-C2); 110.7 (***S***-C2); 110.6 (***R***-C5); 110.6 (***S***-C5); 60.9 (***R***-Cγ); 60.5 (***S***-Cγ); 55.7 (***R***-4-OMe); 55.7 (***S***-4-OMe); 55.5 (***R***-3-OMe); 55.4 (***S***-3-OMe); 54.1 (***R***-C7′); 54.1 (***S***-C7′); 52.8 (***R***-C2′); 52.7 (***S***-C2′); 47.9 (***R***-Cβ); 47.5 (***S***-Cβ); 43.0 (***R***-C9′); 43.0 (***S***-C9′); 31.3 (***R***-C3′); 31.3 (***R***-C5′); 31.2 (***S***-C5′); 30.6 (***S***-C3′); 26.9 (***R***-C6′); 26.8 (***S***-C6′).

Synthesis of ethyl 3,4-dimethoxy-cinnamate (Fig. 5). A refluxing mixture of tetrahydrofuran (80 mL), 3.4-dimethoxy-benzaldehyde (8.8 g, 53.1 mmol), and sodium hydride (2.9 g, 122.1 mmol, 4.9 g of 60% dispersion in mineral oil) was prepared in a 3-necked 250-mL round-bottom flask equipped with a 50-mL cylindrical addition funnel. A solution of triethyl phosphonoacetate (12.5 g, 55.8 mmol) dissolved in tetrahydrofuran (80 mL) was added dropwise to the refluxing mixture over the course of 30 min and the reaction was allowed to proceed for an additional 60 min before it was cooled to room temperature. Excess sodium hydride was then quenched with the dropwise addition of anhydrous ethanol (15 mL) and saturated NH_4Cl (15 mL). Solvents were then evaporated *in vacuo* and the resulting oil was dissolved in ethyl acetate and washed twice with saturated NH_4Cl (15 mL), twice with H_2O, and once with brine. The organic layer was dried over $MgSO_4$ and solvent again evaporated *in vacuo*. This procedure was carried out a second time, and after pooling the reaction products, ethyl 3,4-dimethoxy-cinnamate was crystallized from ethyl acetate and hexane (22.8 g, 91% yield).

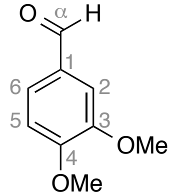

3,4-Dimethoxy-benzaldehyde [from Sigma-Aldrich]:
^{1}H NMR (700 MHz, acetone-d_6) δ 9.87 (s, 1H, Hα); 7.54 (dd, 1H, *J* = 8.2, 1.9 Hz, H6); 7.41 (d, 1H, *J* = 1.9 Hz, H2); 7.15 (d, 1H, *J* = 8.2 Hz, H5); 3.93 (s, 3H, 4-OMe); 3.89 (s, 3H, 3-OMe).
^{13}C NMR (176 MHz, acetone-d_6) δ 191.3 (Cα); 155.6 (C4); 150.7 (C3); 131.2 (C1); 126.8 (C6); 111.8 (C2); 110.1 (C5); 56.3 (4-OMe); 56.0 (3-OMe).

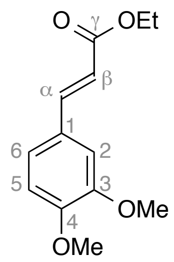

Ethyl 3,4-dimethoxy-cinnamate [synthetic]:
^{1}H NMR (700 MHz, acetone-d_6) δ 7.60 (d, 1H, J = 15.9 Hz, Hα); 7.33 (d, 1H, J = 2.0 Hz, H2); 7.19 (dd, 1H, J = 8.3, 2.0 Hz, H6); 6.98 (d, 1H, J = 8.3 Hz, H5); 6.42 (d, 1H, J = 15.9 Hz, Hβ); 4.19 (q, 2H, J = 7.1 Hz, Et-methylene$_{a/b}$); 3.88 (s, 3H, 4-OMe); 3.85 (s, 3H, 3-OMe); 1.28 (t, 3H, J = 7.1 Hz, Et-methyl$_{a/b/c}$).
^{13}C NMR (176 MHz, acetone-d_6) δ 167.3 (Cγ); 152.4 (C4); 150.5 (C3); 145.2 (Cα); 128.2 (C1); 123.5 (C6); 116.5 (Cβ); 112.2 (C5); 110.9 (C2); 60.5 (Et-methylene); 56.0 (4-OMe); 56.0 (3-OMe); 14.6 (Et-methyl).

Synthesis of 3,4-dimethoxy-cinnamyl alcohol (Fig. 5). To a magnetically stirred solution of Ethyl 3,4-dimethoxy-cinnamate (11.3 g, 47.8 mmol) dissolved in tetrahydrofuran (50 mL) in a 250-mL round-bottom flask maintained under an inert atmosphere at 0 °C, diisobutylaluminum hydride (DIBAL-H, 17.0 g, 120.0 mmol, 120.0 mL of 1.0 M DIBAL-H solution in tetrahydrofuran) was added dropwise. After 2 h, anhydrous ethanol (15 mL) was added dropwise over a period of 10 min to quench the remaining DIBAL-H. A saturated aqueous solution of Rochelle salt (potassium sodium tartrate, 40 mL) was then added dropwise over the course of 20 min and the mixture was allowed to mix for an additional 2 h in order to hydrolyze DIBAL adducts. H_2O (50 mL) and ethyl acetate (30 mL) was then added to the mixture and the organic layer was collected. Five additional ethyl acetate extractions were applied to the aqueous layer and the organic fractions were pooled. The solvents were then evaporated *in vacuo*, affording 3,4-dimethoxy-cinnamyl alcohol. This procedure was carried out a second time, and after pooling the reaction products products, 3,4-dimethoxy-cinnamyl alcohol was obtained as an oil (16.5 g, 89% yield).

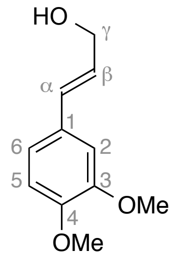

3,4-Dimethoxy-cinnamyl alcohol [synthetic]:
^{1}H NMR (700 MHz, acetone-d_6) δ 7.06 (d, 1H, J = 2.0 Hz, H2); 6.91 (dd, 1H, J = 8.2, 2.0 Hz, H6); 6.87 (d, 1H, J = 8.2 Hz, H5); 6.52 (d, 1H, J = 15.9 Hz, Hα); 6.27 (dt, 1H, J = 15.9, 5.6 Hz, Hβ); 4.21 (td, 2H, J = 5.6, 1.7 Hz, Hγ$_{a/b}$); 3.85 (t, 1H, J = 5.6 Hz, γ-OH); 3.82 (s, 3H, 4-OMe); 3.79 (s, 3H, 3-OMe).
^{13}C NMR (176 MHz, acetone-d_6) δ 150.3 (C4); 149.9 (C3); 131.2 (C1); 130.0 (Cα); 128.7 (Cβ); 120.1 (C6); 112.5 (C5); 110.1 (C2); 63.3 (Cγ); 56.0 (3-OMe); 55.9 (4-OMe).

Synthesis of 3,4-dimethoxy-cinnamaldehyde (Fig. 5). A solution of 3,4-dimethoxy-cinnamyl alcohol (16.4 g, 84.4 mmol) dissolved in 1,4-dioxane (60 mL) was prepared in a 250-mL round-bottom flask. 2,3-dichloro-5,6-dicyano-1,4-benzoquinone (DDQ, 20.1 g, 88.6 mmol) was added to the solution and the contents were stirred at room temperature for 30 min. The reaction mixture was then filtered for the removal of DDQ-H. The solvent was evaporated *in vacuo* and the resulting residue was purified by flash chromatography using Hexane/EtOAc = 1/1 (R_f = 0.82). 3,4-Dimethoxy-cinnamaldehyde was then crystallized from ethyl acetate and hexane (11.7 g, 72% yield).

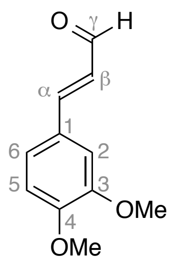

3,4-Dimethoxy-cinnamaldehyde [synthetic]:
^{1}H NMR (700 MHz, acetone-d_6) δ 9.65 (d, 1H, J = 7.7 Hz, Hγ); 7.58 (d, 1H, J = 15.8 Hz, Hα); 7.36 (d, 1H, J = 2.0 Hz, H2); 7.27 (dd, 1H, J = 8.3, 2.0 Hz, H6); 7.03 (d, 1H, J = 8.3 Hz, H5); 6.69 (dd, 1H, J = 15.8, 7.7 Hz, Hβ); 3.89 (s, 3H, 3-OMe); 3.87 (s, 3H, 4-OMe).
^{13}C NMR (176 MHz, acetone-d_6) δ 193.9 (Cγ); 153.7 (Cα); 153.1 (C4); 150.6 (C3); 128.1 (C1); 127.5 (Cβ); 124.3 (C6); 112.3 (C5); 111.1 (C2); 56.1 (4-OMe); 56.1 (3-OMe).

Synthesis of 3,4-dimethoxy-cinnamaldehyde dimethyl acetal (Fig. 5). A solution of 3,4-dimethoxy-cinnamaldehyde (11.4 g, 59.3 mmol) and trimethyl orthoformate (18.9 g, 177.9 mmol, 19.5 mL) in anhydrous methanol (100 mL) was prepared in a 250-mL round-bottom flask. *p*-Toluene sulfonic acid (*p*TSA, 0.4 g, 2.1 mmol) was added to the solution and the contents were stirred at room temperature for 3 hr. The reaction mixture was then poured into a 2:1 emulsion of EtOAc and cold aqueous $NaHCO_3$ (saturated). The organic layer was then washed once with 1N NaOH, twice with saturated Na_2CO_3, twice with water, and once with brine. After drying over $MgSO_4$, the solvent was evaporated *in vacuo* and 3,4-dimethoxy-cinnamaldehyde dimethyl acetal was crystallized from ethyl acetate and hexane (13.6 g, 96% yield).

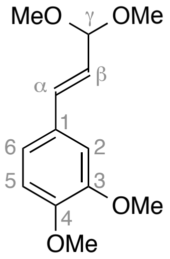

3,4-Dimethoxy-cinnamaldehyde dimethyl acetal [synthetic]:
^{1}H NMR (700 MHz, acetone-d_6) δ 7.13 (d, 1H, J = 1.9 Hz, H2); 6.98 (dd, 1H, J = 8.3, 1.9 Hz, H6); 6.91 (d, 1H, J = 8.3 Hz, H5); 6.63 (d, 1H, J = 16.1 Hz, Hα); 6.08 (dd, 1H, J = 16.1, 5.1 Hz, Hβ); 4.90 (d, 1H, J = 5.1 Hz, Hγ); 3.84 (s, 3H, 4-OMe); 3.80 (s, 3H, 3-OMe); 3.29 (s, 6H, $\gamma_{a/b}$-OMe).
^{13}C NMR (176 MHz, acetone-d_6) δ 150.5 (C3); 150.4 (C4); 133.6 (Cα); 130.1 (C1); 124.9 (Cβ); 120.9 (C6); 112.4 (C5); 110.3 (C2); 104.0 (Cγ); 56.0 (4-OMe); 56.0 (3-OMe); 52.6 (γ_a-OMe); 52.6 (γ_b-OMe).

Synthesis of γ,γ-dimethoxy-α-veratryl-α(*R*),β(*S*)-propanediol (Fig. 5). To a magnetically stirred 1:1 solution of *t*-butanol and H_2O (150 mL each) in a 250-mL round-bottom flask, AD mix β (28.2 g) and methanesulfonamide (1.9 g, 20.1 mmol) were dissolved at room temperature. The temperature of the mixture was lowered to 4 °C, and 3,4-dimethoxy-cinnamaldehyde dimethyl acetal (4.8 g, 20.1 mmol) was added, and the contents were mixed for 18 h. The reaction mixture was then poured into a 2:1 emulsion of EtOAc and H_2O. The aqueous layer was then extracted from four times with EtOAc. After drying over $MgSO_4$, the solvent was evaporated *in vacuo* and the residue was purified further by flash chromatography (1/3 Hexane/EtOAc, R_f = 0.14), affording γ,γ-dimethoxy-α-veratryl-α(*R*),β(*S*)-propanediol as an oil (3.9 g, 72% yield).

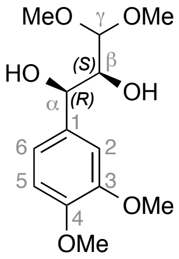

γ,γ-Dimethoxy-α-veratryl-α(*R*),β(*S*)-propanediol [synthetic]:
^{1}H NMR (700 MHz, acetone-d_6) δ 7.04 (d, 1H, J = 1.9 Hz, H2); 6.91 (dd, 1H, J = 8.2, 1.9 Hz, H6); 6.87 (d, 1H, J = 8.2 Hz, H5); 4.74 (dd, 1H, J = 6.2, 2.8 Hz, Hα); 4.35 (d, 1H, J = 6.1 Hz, Hγ); 4.07 (d, 1H, J = 6.2 Hz, α-OH); 3.79 (s, 3H, 3-OMe); 3.78 (s, 3H, 4-OMe); 3.73 (d, 1H, J = 6.1 Hz, β-OH); 3.61 (td, 1H, J = 6.1, 2.8 Hz, Hβ); 3.41 (s, 3H, γ_a-OMe); 3.38 (s, 3H, γ_b-OMe).
^{13}C NMR (176 MHz, acetone-d_6) δ 149.8 (C3); 149.2 (C4); 136.6 (C1); 119.4 (C6); 112.2 (C5); 111.4 (C2); 105.6 (Cγ); 75.6 (Cβ); 72.9 (Cα); 56.0 (4-OMe); 55.9 (3-OMe); 55.5 (γ_a-OMe); 54.3 (γ_b-OMe).

Synthesis of γ,γ-dimethoxy-β(*R*)-hydroxy-α-veratrylpropanone (Fig. 5). A solution of γ,γ-dimethoxy-α-veratryl-α(*R*),β(*S*)-propanediol (3.9 g, 14.5 mmol) dissolved in 1,4-dioxane (25 mL) was prepared in a 250-mL round-bottom flask. 2,3-dichloro-5,6-dicyano-1,4-benzoquinone (DDQ, 3.6 g, 15.9 mmol) was added to the solution and the contents were stirred at room temperature for 30 min. The reaction mixture was then filtered for the removal of DDQ-H. The solvent was evaporated *in vacuo* and the resulting residue was purified by flash chromatography (1/1 Hexane/EtOAc, R_f = 0.22), yielding γ,γ-dimethoxy-β(*R*)-hydroxy-α-veratrylpropanone as an oil (2.9 g, 73% yield).

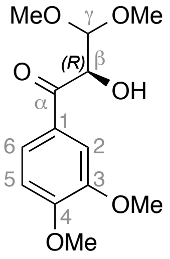

γ,γ-Dimethoxy-β(*R*)-hydroxy-α-veratrylpropanone [synthetic]:
^{1}H NMR (700 MHz, acetone-d_6) δ 7.72 (dd, 1H, *J* = 8.5, 2.0 Hz, H6); 7.57 (d, 1H, *J* = 2.0 Hz, H2); 7.04 (d, 1H, *J* = 8.5 Hz, H5); 5.05 (dd, 1H, *J* = 7.7, 5.1 Hz, Hβ); 4.52 (d, 1H, *J* = 5.1 Hz, Hγ); 4.38 (d, 1H, *J* = 7.7 Hz, β-OH); 3.90 (s, 3H, 4-OMe); 3.87 (s, 3H, 3-OMe); 3.43 (s, 3H, $γ_a$-OMe); 3.32 (s, 3H, $γ_b$-OMe).
^{13}C NMR (176 MHz, acetone-d_6) δ 197.6 (Cα); 154.8 (C4); 149.9 (C3); 129.4 (C1); 124.8 (C6); 112.0 (C2); 111.2 (C5); 106.8 (Cγ); 73.5 (Cβ); 56.1 (4-OMe); 56.1 (3-OMe); 56.0 ($γ_b$-OMe); 54.9 ($γ_a$-OMe).

Synthesis of γ,γ-dimethoxy-β(*R*)-O-triflyl-α-veratrylpropanone (Fig. 5). To a magnetically stirred solution of γ,γ-dimethoxy-β(*R*)-hydroxy-α-veratrylpropanone (0.7 g, 2.6 mmol) and CH_2Cl_2 (16 mL) held in a 100-mL round-bottom flask under inert atmosphere at room temperature, 2,6-lutidine (0.4 g, 3.4 mmol, 0.4 mL) was added and the temperature lowered to 0 °C. Trifluoromethanesulfonic anhydride (1.1 g, 3.9 mmol, 3.9 mL of a 1.0 M solution in CH_2Cl_2) was then added and the contents were stirred at room temperature for 2 h as the mixture was brought back to room temperature. The solvent was evaporated *in vacuo* and the resulting residue was purified by flash chromatography (1/1 Hexane/EtOAc, R_f = 0.72), yielding γ,γ-dimethoxy-β(*R*)-O-triflyl-α-veratrylpropanone as an oil (0.8 g, 75% yield).

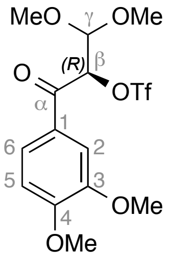

γ,γ-Dimethoxy-β(*R*)-O-triflyl-α-veratrylpropanone [synthetic]:
^{1}H NMR (700 MHz, acetone-d_6) δ 7.78 (dd, 1H, *J* = 8.5, 2.1 Hz, H6); 7.56 (d, 1H, *J* = 2.1 Hz, H2); 7.12 (d, 1H, *J* = 8.5 Hz, H5); 6.18 (d, 1H, *J* = 5.3 Hz, Hβ); 4.89 (d, 1H, *J* = 5.3 Hz, Hγ); 3.94 (s, 3H, 4-OMe); 3.90 (s, 3H, 3-OMe); 3.49 (s, 3H, $γ_a$-OMe); 3.41 (s, 3H, $γ_b$-OMe).
^{13}C NMR (176 MHz, acetone-d_6) δ 188.9 (Cα); 155.8 (C4); 150.4 (C3); 128.2 (C1); 125.2 (C6); 119.3 (CF_3); 111.5 (C2); 111.5 (C5); 103.7 (Cγ); 84.7 (Cβ); 56.9 ($γ_b$-OMe); 56.3 (4-OMe); 56.1 (3-OMe); 56.0 ($γ_a$-OMe).

Synthesis of γ,γ-dimethoxy-β(*S*)-S-(methyl N-acetyl-cysteinyl)-α-veratrylpropanone, CS-β(*S*)VP (Fig. 5). To a magnetically stirred solution of N-acetyl cysteine methyl ester (0.3 g, 1.6 mmol), K_2CO_3 (0.2 g, 1.7 mmol), and anhydrous dimethyl formamide held in a 100-mL round-bottom flask under inert atmosphere at room temperature, γ,γ-dimethoxy-β(*R*)-O-triflyl-α-veratrylpropanone (0.7 g, 1.6 mmol) was added and the contents were stirred at room temperature for 2 h. The mixture was poured into a 1:1 emulsion of EtOAc and H_2O, and the organic layer was washed once with saturated $NaHCO_3$, once with saturated Na_2CO_3, twice with 1N NaOH, twice with H_2O, and once with brine. After drying the organic layer over $MgSO_4$, solvents were evaporated *in vacuo*, affording γ,γ-dimethoxy-β(*S*)-S-(N-acetyl methyl ester)-α-veratrylpropanone, CS-β(*S*)VP as an oil (0.4 g, 53% yield).

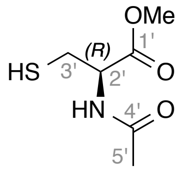

Methyl N-acetyl-2′(*R*)-cysteinate [from Sigma-Aldrich]:
^{1}H NMR (700 MHz, acetone-d_6) δ 7.53 (bs, 1H, NH); 4.70 – 4.66 (m, 1H, H2′); 3.70 (s, 3H, 1′-OOMe); 2.96 – 2.85 (m, 2H, $H3'_{a/b}$); 1.96 (s, 3H, 5′).
^{13}C NMR (176 MHz, acetone-d_6) δ 171.4 (C1′); 170.2 (C4′); 55.2 (C2′); 55.1 (1′-OOMe); 52.5 (C3′); 26.7 (C5′).

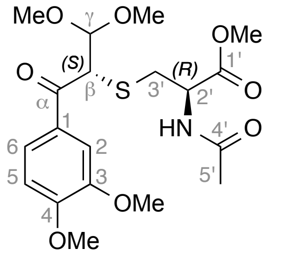

γ,γ-Dimethoxy-β(*S*)-S-(methyl N-acetyl-cysteinyl)-α-veratrylpropanone, CS-β(*S*)VP [synthetic]:

^{1}H NMR (700 MHz, D_2O) δ 7.68 (dd, 1H, *J* = 8.4, 1.7 Hz, H6); 7.46 (d, 1H, *J* = 1.7 Hz, H2); 7.04 (d, 1H, *J* = 8.4 Hz, H5); 4.81 (d, 1H, *J* = 7.6 Hz, Hγ); 4.64 (d, 1H, *J* = 7.6 Hz, Hβ); 4.39 (dd, 1H, *J* = 8.4, 5.0 Hz, H2′); 3.84 (s, 3H, 4-OMe); 3.81 (s, 3H, 3-OMe); 3.54 (s, 3H, 1′-OOMe); 3.41 (s, 3H, γ_a-OMe); 3.30 (s, 3H, γ_b-OMe); 3.00 (dd, 1H, *J* = 14.2, 5.0 Hz, $H3'_a$); 2.86 (dd, 1H, *J* = 14.2, 8.4 Hz, $H3'_b$); 1.77 (s, 3H, 5′).

^{13}C NMR (176 MHz, D_2O) δ 197.1 (Cα); 174.7 (C4′); 172.9 (C1′); 154.8 (C4); 149.2 (C3); 129.0 (C1); 125.6 (C6); 111.8 (C2); 111.6 (C5); 105.3 (Cγ); 57.0 (γ_b-OMe); 56.7 (4-OMe); 56.5 (3-OMe); 55.8 (γ_a-OMe); 53.8 (1′-OOMe); 53.1 (C2′); 50.0 (Cβ); 32.1 (C3′); 22.1 (C5′).

^{1}H NMR (700 MHz, acetone-d_6) δ 7.76 (dd, 1H, *J* = 8.4, 2.1 Hz, H6); 7.56 (d, 1H, *J* = 2.1 Hz, H2); 7.50 (d, 1H, *J* = 7.7 Hz, NH); 7.07 (d, 1H, *J* = 8.4 Hz, H5); 4.83 (d, 1H, *J* = 8.5 Hz, Hγ); 4.67 (ddd, 1H, *J* = 7.7, 6.6, 6.2 Hz, H2′); 4.64 (d, 1H, *J* = 8.5 Hz, Hβ); 3.92 (s, 3H, 4-OMe); 3.89 (s, 3H, 3-OMe); 3.63 (s, 3H, 1′-OOMe); 3.50 (s, 3H, γ_a-OMe); 3.30 (s, 3H, γ_b-OMe); 3.06 (m, 2H, $H3'_{a/b}$); 1.92 (s, 3H, 5′).
^{13}C NMR (176 MHz, acetone-d_6) δ 193.8 (Cα); 171.8 (C1′); 170.0 (C4′); 155.0 (C4); 150.3 (C3); 129.8 (C1); 124.2 (C6); 111.7 (C2); 111.4 (C5); 106.5 (Cγ); 56.2 (4-OMe); 56.1 (3-OMe); 55.9 (γ_b-OMe); 54.8 (γ_a-OMe); 53.3 (1′-OOMe); 52.4 (C2′); 49.6 (Cβ); 33.6 (C3′); 22.6 (C5′).

Synthesis of γ,γ-dimethoxy-α-veratryl-α(*S*),β(*R*)-propanediol (Fig. 5). To a magnetically stirred 1:1 solution of *t*-butanol and H_2O (190 mL each) in a 250-mL round-bottom flask, AD mix α (50.8 g) and methanesulfonamide (3.5 g, 36.3 mmol) were dissolved at room temperature. The temperature of the mixture was lowered to 4 °C, and 3,4-dimethoxy-cinnamaldehyde dimethyl acetal (8.6 g, 36.3 mmol) was added, and the contents were mixed for 18 h. The reaction mixture was then poured into a 2:1 emulsion of EtOAc and H_2O. The aqueous layer was then extracted from four times with EtOAc. After drying over $MgSO_4$, the solvent was evaporated *in vacuo* and the residue was purified further by flash chromatography (1/3 Hexane/EtOAc, R_f = 0.14), affording γ,γ-dimethoxy-α-veratryl-α(*S*),β(*R*)-propanediol as an oil (8.2 g, 83% yield).

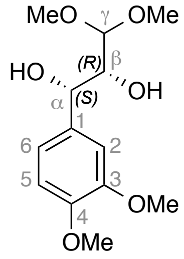

γ,γ-Dimethoxy-α-veratryl-α(*S*),β(*R*)-propanediol [synthetic]:

^{1}H NMR (700 MHz, acetone-d_6) δ 7.04 (d, 1H, *J* = 1.9 Hz, H2); 6.91 (dd, 1H, *J* = 8.2, 1.9 Hz, H6); 6.87 (d, 1H, *J* = 8.2 Hz, H5); 4.74 (dd, 1H, *J* = 6.2, 2.8 Hz, Hα); 4.35 (d, 1H, *J* = 6.1 Hz, Hγ); 4.05 (d, 1H, *J* = 6.2 Hz, α-OH); 3.79 (s, 3H, 3-OMe); 3.78 (s, 3H, 4-OMe); 3.71 (d, 1H, *J* = 6.1 Hz, β-OH); 3.60 (td, 1H, *J* = 6.1, 2.8 Hz, Hβ); 3.41 (s, 3H, γ_a-OMe); 3.38 (s, 3H, γ_b-OMe).

^{13}C NMR (176 MHz, acetone-d_6) δ 149.8 (C3); 149.2 (C4); 136.6 (C1); 119.4 (C6); 112.2 (C5); 111.4 (C2); 105.6 (Cγ); 75.6 (Cβ); 72.9 (Cα); 56.0 (4-OMe); 55.9 (3-OMe); 55.5 (γ_a-OMe); 54.3 (γ_b-OMe).

Synthesis of γ,γ-dimethoxy-β(*S*)-hydroxy-α-veratrylpropanone (Fig. 5). A solution of γ,γ-dimethoxy-α-veratryl-α(*S*),β(*R*)-propanediol (8.9 g, 32.7 mmol) dissolved in 1,4-dioxane (45 mL) was prepared in a 250-mL round-bottom flask. 2,3-dichloro-5,6-dicyano-1,4-benzoquinone (DDQ, 7.8 g, 34.4 mmol) was added to the solution and the contents were stirred at room temperature for 30 min. The reaction mixture was then filtered for the removal of DDQ-H. The solvent was evaporated *in vacuo* and the resulting residue was purified by flash chromatography (1/1 Hexane/EtOAc, R_f = 0.22), yielding γ,γ-dimethoxy-β(*S*)-hydroxy-α-veratrylpropanone as an oil (7.1 g, 81% yield).

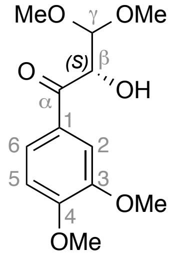

γ,γ-Dimethoxy-β(*S*)-hydroxy-α-veratrylpropanone [synthetic]:
^{1}H NMR (700 MHz, acetone-d_6) δ 7.72 (dd, 1H, J = 8.5, 2.0 Hz, H6); 7.57 (d, 1H, J = 2.0 Hz, H2); 7.04 (d, 1H, J = 8.5 Hz, H5); 5.04 (dd, 1H, J = 7.7, 5.1 Hz, Hβ); 4.52 (d, 1H, J = 5.1 Hz, Hγ); 4.36 (d, 1H, J = 7.7 Hz, β-OH); 3.90 (s, 3H, 4-OMe); 3.87 (s, 3H, 3-OMe); 3.43 (s, 3H, $γ_a$-OMe); 3.32 (s, 3H, $γ_b$-OMe).
^{13}C NMR (176 MHz, acetone-d_6) δ 197.6 (Cα); 154.8 (C4); 149.9 (C3); 129.4 (C1); 124.8 (C6); 112.0 (C2); 111.2 (C5); 106.8 (Cγ); 73.5 (Cβ); 56.1 (4-OMe); 56.1 (3-OMe); 56.0 ($γ_b$-OMe); 54.9 ($γ_a$-OMe).

Synthesis of γ,γ-dimethoxy-β(*S*)-O-triflyl-α-veratrylpropanone (Fig. 5). To a magnetically stirred solution of γ,γ-dimethoxy-β(*S*)-hydroxy-α-veratrylpropanone (0.6 g, 2.2 mmol) and CH_2Cl_2 (16 mL) held in a 100-mL round-bottom flask under inert atmosphere at room temperature, 2,6-lutidine (0.3 g, 2.9 mmol, 0.3 mL) was added and the temperature lowered to 0 °C. Trifluoromethanesulfonic anhydride (0.9 g, 3.3 mmol, 3.3 mL of a 1.0 M solution in CH_2Cl_2) was then added and the contents were stirred at room temperature for 2 h as the mixture was brought back to room temperature. The solvent was evaporated *in vacuo* and the resulting residue was purified by flash chromatography (1/1 Hexane/EtOAc, R_f = 0.72), yielding γ,γ-dimethoxy-β(*S*)-O-triflyl-α-veratrylpropanone as an oil (0.7 g, 59% yield).

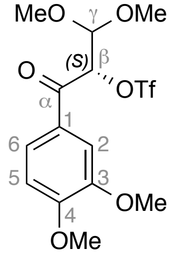

γ,γ-Dimethoxy-β(*S*)-O-triflyl-α-veratrylpropanone:
^{1}H NMR (700 MHz, acetone-d_6) δ 7.78 (dd, 1H, J = 8.5, 2.1 Hz, H6); 7.56 (d, 1H, J = 2.1 Hz, H2); 7.12 (d, 1H, J = 8.5 Hz, H5); 6.18 (d, 1H, J = 5.3 Hz, Hβ); 4.89 (d, 1H, J = 5.3 Hz, Hγ); 3.94 (s, 3H, 4-OMe); 3.90 (s, 3H, 3-OMe); 3.49 (s, 3H, $γ_a$-OMe); 3.41 (s, 3H, $γ_b$-OMe).
^{13}C NMR (176 MHz, acetone-d_6) δ 188.9 (Cα); 155.8 (C4); 150.4 (C3); 128.2 (C1); 125.2 (C6); 119.3 (β-OSO_2-CF_3); 111.5 (C2); 111.5 (C5); 103.7 (Cγ); 84.7 (Cβ); 56.9 ($γ_b$-OMe); 56.3 (4-OMe); 56.1 (3-OMe); 56.0 ($γ_a$-OMe).

Synthesis of γ,γ-dimethoxy-β(*R*)-S-(methyl N-acetyl-cysteinyl)-α-veratrylpropanone, CS-β(*R*)VP (Fig. 5). To a magnetically stirred solution of N-acetyl cysteine methyl ester (0.2 g, 1.3 mmol), K_2CO_3 (0.2 g, 1.4 mmol), and anhydrous dimethyl formamide held in a 100-mL round-bottom flask under inert atmosphere at room temperature, γ,γ-dimethoxy-β(*S*)-O-triflyl-α-veratrylpropanone (0.5 g, 1.3 mmol) was added and the contents were stirred at room temperature for 2 h. The mixture was poured into a 1:1 emulsion of EtOAc and H_2O, and the organic layer was washed once with saturated $NaHCO_3$, once with saturated Na_2CO_3, twice with 1N NaOH, twice with H_2O, and once with brine. After drying the organic layer over $MgSO_4$, solvents were evaporated *in vacuo*, affording γ,γ-dimethoxy-β(*R*)-S-(N-acetyl methyl ester)-α-veratrylpropanone, CS-β(*R*)VP as an oil (0.4 g, 73% yield).

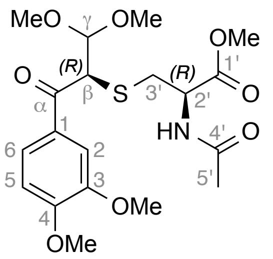

γ,γ-Dimethoxy-β(*R*)-S-(methyl N-acetyl-cysteinyl)-α-veratrylpropanone, CS-β(*R*)VP [synthetic]:

^{1}H NMR (700 MHz, D_2O) δ 7.67 (dd, 1H, *J* = 8.5, 2.0 Hz, H6); 7.45 (d, 1H, *J* = 2.0 Hz, H2); 7.03 (d, 1H, *J* = 8.5 Hz, H5); 4.79 (d, 1H, *J* = 7.6 Hz, Hγ); 4.62 (d, 1H, *J* = 7.6 Hz, Hβ); 4.37 (dd, 1H, *J* = 8.8, 4.7 Hz, H2´); 3.84 (s, 3H, 4-OMe); 3.80 (s, 3H, 3-OMe); 3.53 (s, 3H, 1´-OOMe); 3.42 (s, 3H, γ_a-OMe); 3.30 (s, 3H, γ_b-OMe); 3.11 (dd, 1H, *J* = 14.0, 4.7 Hz, H3´$_a$); 2.80 (dd, 1H, *J* = 14.0, 8.8 Hz, H3´$_b$); 1.74 (s, 3H, 5´).

^{13}C NMR (176 MHz, D_2O) δ 196.9 (Cα); 174.7 (C4´); 172.9 (C1´); 154.8 (C4); 149.2 (C3); 128.9 (C1); 125.5 (C6); 111.8 (C2); 111.6 (C5); 105.4 (Cγ); 57.0 (γ_b-OMe); 56.7 (4-OMe); 56.5 (3-OMe); 55.9 (γ_a-OMe); 53.8 (1´-OOMe); 53.1 (C2´); 50.0 (Cβ); 32.3 (C3´); 22.1 (C5´).

^{1}H NMR (700 MHz, acetone-d_6) δ 7.73 (dd, 1H, *J* = 8.5, 2.1 Hz, H6); 7.55 (d, 1H, *J* = 2.1 Hz, H2); 7.44 (d, 1H, *J* = 7.7 Hz, NH); 7.05 (d, 1H, *J* = 8.5 Hz, H5); 4.85 (d, 1H, *J* = 8.4 Hz, Hγ); 4.70 (ddd, 1H, *J* = 7.7, 7.6, 5.2 Hz, H2´); 4.62 (d, 1H, *J* = 8.4 Hz, Hβ); 3.91 (s, 3H, 4-OMe); 3.89 (s, 3H, 3-OMe); 3.65 (s, 3H, 1´-OOMe); 3.49 (s, 3H, γ_a-OMe); 3.31 (s, 3H, γ_b-OMe); 3.21 (dd, 1H, *J* = 13.5, 5.2 Hz, H3´$_a$); 2.94 (dd, 1H, *J* = 13.5, 7.6 Hz, H3´$_b$); 1.89 (s, 3H, 5´).

^{13}C NMR (176 MHz, acetone-d_6) δ 193.4 (Cα); 171.8 (C1´); 170.0 (C4´); 154.9 (C4); 150.2 (C3); 129.7 (C1); 124.2 (C6); 111.7 (C2); 111.4 (C5); 106.0 (Cγ); 56.2 (4-OMe); 56.1 (3-OMe); 56.0 (γ_b-OMe); 54.2 (γ_a-OMe); 52.8 (C2´); 52.5 (1´-OOMe); 48.7 (Cβ); 33.0 (C3´); 22.6 (C5´).

IN VITRO ENZYMATIC SYNTHESES:

***In vitro* enzymatic synthesis of GS-β(*S*)VG from LigE or LigP (Fig. S1).** β(*R*)GVG (1.7 mg, 1.0 mM) and either LigE (1.0 mg, 6.2 μM) or LigP (1.0 mg, 6.5 μM) were added to 5 mL of assay buffer. Reactions were monitored and analyzed according to the methods described in the main text. The ^{1}H, ^{13}C, COSY, HSQC, and HMBC NMR spectra of the isolated products from both LigE and LigP were found to be identical to those of the chemically synthesized GS-βVG (Fig. S1A).

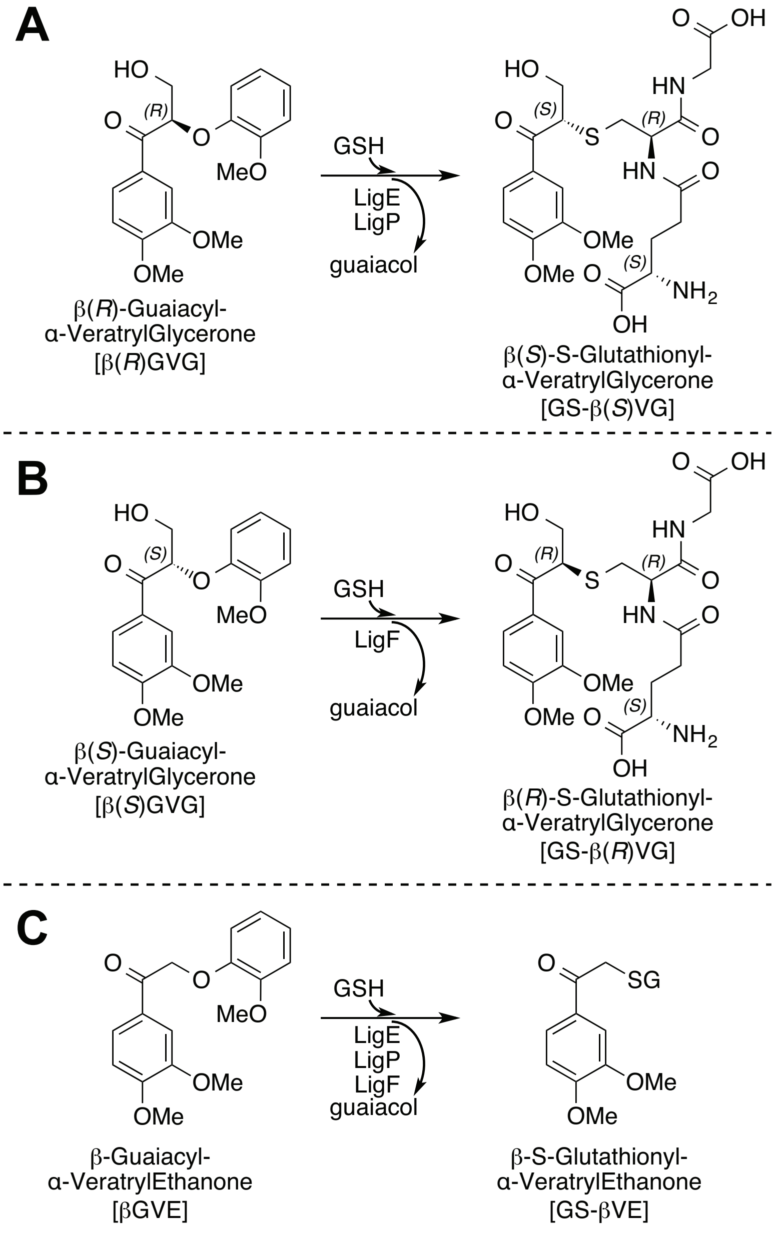

Fig. S1. Scheme for enzymatic synthesis of (**A**) β(*S*)-S-glutathionyl-α-veratrylglycerone [GS-β(*S*)VG] using either LigE or LigP, (**B**) β(*R*)-S-glutathionyl-α-veratrylglycerone [GS-β(*R*)VG] using LigF, and (**C**) β-S-glutathionyl-α-veratrylethanone using LigE, LigP, or LigF.

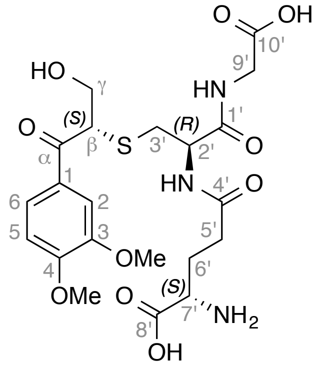

β(*S*)-S-glutathionyl-α-veratrylglycerone, GS-β(*S*)VG [from LigE]: ^{1}H NMR (700 MHz, D_2O) δ 7.68 (dd, 1H, *J* = 8.4, 2.0 Hz, H6); 7.48 (d, 1H, *J* = 2.0 Hz, H2); 7.02 (d, 1 H, *J* = 8.4 Hz, H5); 4.54 (dd, 1H, *J* = 7.9, 5.9 Hz, Hβ); 4.27 (dd, 1H, *J* = 8.8, 5.1 Hz, H2′); 3.98 (dd, 1H, *J* = 11.6, 7.9 Hz, Hγ_a); 3.84 (s, 3H, 4-OMe); 3.83 - 3.78 (m, 1H, Hγ_b); 3.80 (s, 3H, 3-OMe); 3.57 (d, 1H, *J* = 17.3 Hz, H9′$_a$); 3.50 (d, 1H, *J* = 17.3 Hz, H9′$_b$); 3.49 (t, 1H, *J* = 6.4 Hz, H7′); 2.94 (dd, 1H, *J* = 14.2, 5.1 Hz, H3′$_a$); 2.77 (dd, 1H, *J* = 14.2, 8.8 Hz, H3′$_b$); 2.26 – 2.16 (m, 2H, H5′$_{a/b}$); 1.90 – 1.80 (m, 2H, H6′$_{a/b}$). ^{13}C NMR (176 MHz, D_2O) δ 197.2 (Cα); 175.8 (C10′); 174.3 (C4′); 173.7 (C8′); 171.1 (C1′); 153.4 (C4); 148.0 (C3); 127.8 (C1); 124.2 (C6); 110.7 (C2); 110.6 (C5); 60.5 (Cγ); 55.7 (4-OMe); 55.5 (3-OMe); 53.8 (C7′); 52.8 (C2′); 47.6 (Cβ); 43.0 (C9′); 31.1 (C5′); 30.6 (C3′); 25.9 (C6′).

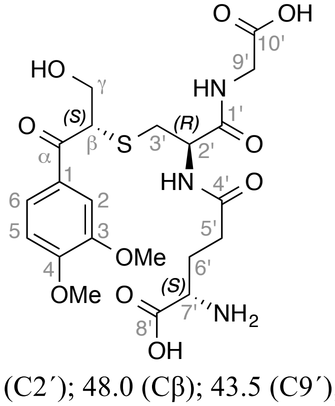

β(*S*)-S-glutathionyl-α-veratrylglycerone, GS-β(*S*)VG [from LigP]: ^{1}H NMR (700 MHz, D_2O) δ 7.69 (dd, 1H, *J* = 8.4, 2.0 Hz, H6); 7.49 (d, 1H, *J* = 2.0 Hz, H2); 7.03 (d, 1H, *J* = 8.4 Hz, H5); 4.54 (dd, 1H, *J* = 7.9, 5.9 Hz, Hβ); 4.27 (dd, 1H, *J* = 8.8, 5.1 Hz, H2′); 3.98 (dd, 1H, *J* = 11.6, 7.9 Hz, Hγ_a); 3.84 (s, 3H, 4-OMe); 3.83 - 3.78 (m, 1H, Hγ_b); 3.81 (s, 3H, 3-OMe); 3.57 (d, 1H, *J* = 17.3 Hz, H9′$_a$); 3.50 (d, 1H, *J* = 17.3 Hz, H9′$_b$); 3.48 (t, 1H, *J* = 6.4 Hz, H7′); 2.94 (dd, 1H, *J* = 14.2, 5.1 Hz, H3′$_a$); 2.77 (dd, 1H, *J* = 14.2, 8.8 Hz, H3′$_b$); 2.26 – 2.15 (m, 2H, H5′$_{a/b}$); 1.90 – 1.80 (m, 2H, H6′$_{a/b}$). ^{13}C NMR (176 MHz, D_2O) δ 198.2 (Cα); 176.9 (C10′); 176.4 (C4′); 175.7 (C8′); 172.3 (C1′); 153.9 (C4); 148.6 (C3); 128.2 (C1); 125.0 (C6); 110.9 (C2); 110.8 (C5); 60.7 (Cγ); 56.1 (4-OMe); 56.0 (3-OMe); 54.1 (C7′); 53.4 (C2′); 48.0 (Cβ); 43.5 (C9′); 31.6 (C5′); 31.3 (C3′); 26.9 (C6′).

***In vitro* enzymatic synthesis of GS-β(*R*)VG from LigF (Fig. S1).** To 5 mL of the enzyme assay buffer, enantiopure β(*S*)GVG (1.7 mg, 1.0 mM) and LigF (1.0 mg, 6.7 μM) were added and the reaction was monitored and analyzed according to the methods described in the main text. The ^{1}H, ^{13}C, COSY, HSQC, and HMBC NMR spectra of the isolated LigF product were found to be identical to those of the chemically synthesized compound GS-βVG (Fig. S1B).

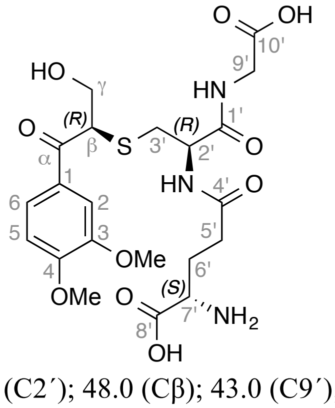

β(*R*)-S-glutathionyl-α-veratrylglycerone, GS-β(*R*)VG [from LigF]: ^{1}H NMR (700 MHz, D_2O) δ 7.67 (dd, 1H, *J* = 8.4, 2.0 Hz, H6); 7.47 (d, 1H, *J* = 2.0 Hz, H2); 7.02 (d, 1H, *J* = 8.4 Hz, H5); 4.58 (dd, 1H, *J* = 8.0, 5.9 Hz, Hβ); 4.35 (dd, 1H, *J* = 9.0, 4.9 Hz, H2′); 3.95 (dd, 1H, *J* = 11.5, 8.0 Hz, Hγ_a); 3.84 (s, 3H, 4-OMe); 3.83 - 3.78 (m, 1H, Hγ_b); 3.80 (s, 3H, 3-OMe); 3.58 (d, 1H, *J* = 17.3 Hz, H9′$_a$); 3.49 (d, 1H, *J* = 17.3 Hz, H9′$_b$); 3.45 (t, 1H, *J* = 6.4 Hz, H7′); 3.04 (dd, 1H, *J* = 14.0, 4.9 Hz, H3′$_a$); 2.69 (dd, 1H, *J* = 14.0, 9.0 Hz, H3′$_b$); 2.26 – 2.16 (m, 2H, H5′$_{a/b}$); 1.90 – 1.80 (m, 2H, H6′$_{a/b}$). ^{13}C NMR (176 MHz, D_2O) δ 197.5 (Cα); 175.8 (C10′); 174.4 (C8′); 173.7 (C4′); 171.1 (C1′); 153.4 (C4); 148.0 (C3); 127.9 (C1); 124.2 (C6); 110.7 (C2); 110.6 (C5); 60.9 (Cγ); 55.7 (4-OMe); 55.5 (3-OMe); 53.8 (C7′); 52.8 (C2′); 48.0 (Cβ); 43.0 (C9′); 31.3 (C3′); 31.1 (C5′); 25.9 (C6′).

***In vitro* enzymatic synthesis of GS-βVE from LigE, LigP, and LigF (Fig. S1).** As described in the main text, βGVE (1.5 mg, 1.0 mM) and either LigE (1.0 mg, 6.2 μM), LigP (1.0 mg, 6.5 μM), or LigF

(1.0 mg, 6.7 μM) was added to 5 mL of assay buffer for enzymatic syntheses of GS-βVEThe ^{1}H, ^{13}C, COSY, HSQC, and HMBC NMR spectra of the isolated products from each of the three enzymes were found to be identical to those of the chemically synthesized compound GS-βVE (Fig. S1C).

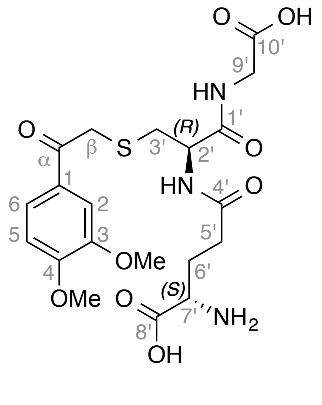

β-S-glutathionyl-α-veratrylethanone, GS-βVE [from LigE]:
^{1}H NMR (700 MHz, D_2O) δ 7.61 (dd, 1H, *J* = 8.4, 2.0 Hz, H6); 7.43 (d, 1H, *J* = 2.0 Hz, H2); 7.00 (d, 1H, *J* = 8.4 Hz, H5); 4.43 (dd, 1H, *J* = 9.1, 4.9 Hz, H2′); 3.82 (s, 3H, 4-OMe); 3.79 (s, 3H, 3-OMe); 3.62 (d, 1H, *J* = 17.3 Hz, H9′$_{a}$); 3.57 (t, 1H, *J* = 6.4 Hz, H7′); 3.56 (d, 1H, *J* = 17.3 Hz, H9′$_{b}$); 2.98 (dd, 1H, *J* = 14.3, 4.9 Hz, H3′$_{a}$); 2.78 (dd, 1H, *J* = 14.3, 9.1 Hz, H3′$_{b}$); 2.33 (t, 2H, *J* = 7.8 Hz, H5′$_{a/b}$); 1.95 (dt, *J* = 7.8, 6.4 Hz, H6′$_{a/b}$).
^{13}C NMR (176 MHz, D_2O) δ 197.8 (Cα); 176.9 (C10′); 175.8 (C4′); 175.3 (C8′); 172.4 (C1′); 154.3 (C4); 148.9 (C3); 128.6 (C1); 125.5 (C6); 111.7 (C2); 111.7 (C5); 56.7 (4-OMe); 56.5 (3-OMe); 55.1 (C7′); 53.5 (C2′); 44.0 (C9′); 37.7 (Cβ); 34.0 (C3′); 32.3 (C5′); 27.7 (C6′).

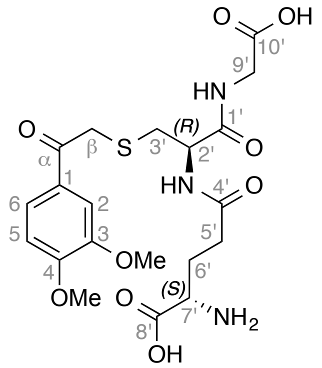

β-S-glutathionyl-α-veratrylethanone, GS-βVE [from LigP]:
^{1}H NMR (700 MHz, D_2O) δ 7.59 (dd, 1H, *J* = 8.4, 2.0 Hz, H6); 7.41 (d, 1H, *J* = 2.0 Hz, H2); 6.98 (d, 1H, *J* = 8.4 Hz, H5); 4.43 (dd, 1H, *J* = 9.1, 4.9 Hz, H2′); 3.82 (s, 3H, 4-OMe); 3.78 (s, 3H, 3-OMe); 3.62 (d, 1H, *J* = 17.3 Hz, H9′$_{a}$); 3.60 (t, 1H, *J* = 6.4 Hz, H7′); 3.56 (d, 1H, *J* = 17.3 Hz, H9′$_{b}$); 2.98 (dd, 1H, *J* = 14.3, 4.9 Hz, H3′$_{a}$); 2.78 (dd, 1H, *J* = 14.3, 9.1 Hz, H3′$_{b}$); 2.34 (t, 2H, *J* = 7.8 Hz, H5′$_{a/b}$); 1.97 (dt, *J* = 7.8, 6.4 Hz, H6′$_{a/b}$).
^{13}C NMR (176 MHz, D_2O) δ 198.1 (Cα); 176.9 (C10′); 175.6 (C4′); 174.8 (C8′); 172.4 (C1′); 154.3 (C4); 148.9 (C3); 128.5 (C1); 125.4 (C6); 111.7 (C2); 111.7 (C5); 56.6 (4-OMe); 56.5 (3-OMe); 54.8 (C7′); 53.5 (C2′); 44.0 (C9′); 37.8 (Cβ); 34.0 (C3′); 32.1 (C5′); 27.0 (C6′).

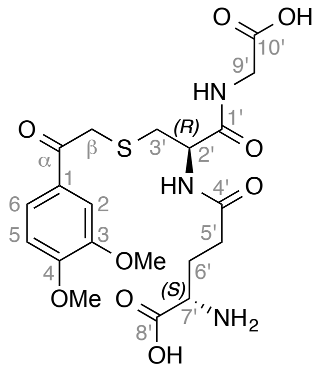

β-S-glutathionyl-α-veratrylethanone, GS-βVE [from LigF]:
^{1}H NMR (700 MHz, D_2O) δ 7.60 (dd, 1H, *J* = 8.4, 2.0 Hz, H6); 7.41 (d, 1H, *J* = 2.0 Hz, H2); 6.99 (d, 1H, *J* = 8.4 Hz, H5); 4.43 (dd, 1H, *J* = 9.1, 4.9 Hz, H2′); 3.82 (s, 3H, 4-OMe); 3.78 (s, 3H, 3-OMe); 3.62 (d, 1H, *J* = 17.3 Hz, H9′$_{a}$); 3.56 (t, 1H, *J* = 6.4 Hz, H7′); 3.56 (d, 1H, *J* = 17.3 Hz, H9′$_{b}$); 2.98 (dd, 1H, *J* = 14.3, 4.9 Hz, H3′$_{a}$); 2.78 (dd, 1H, *J* = 14.3, 9.1 Hz, H3′$_{b}$); 2.33 (t, 2H, *J* = 7.8 Hz, H5′$_{a/b}$); 1.95 (dt, *J* = 7.8, 6.4 Hz, H6′$_{a/b}$).
^{13}C NMR (176 MHz, D_2O) δ 198.1 (Cα); 176.9 (C10′); 175.7 (4′); 175.4 (C8′); 172.4 (C1′); 154.3 (C4); 148.9 (C3); 128.5 (C1); 125.4 (C6); 111.7 (C2); 111.7 (C5); 56.6 (4-OMe); 56.5 (3-OMe); 54.9 (C7′); 53.5 (C2′); 44.0 (C9′); 37.6 (Cβ); 34.0 (C3′); 32.2 (C5′); 27.3 (C6′).

***In vitro* enzymatic synthesis of β-deoxy-α-veratrylglycerone from LigG (Fig. S2).** Chemically synthesized GS-βVG (5.0 mg, 1.0 mM each) and LigG (1.3 mg, 8.5 μM) were added to 5 mL of assay buffer and the reaction was monitored as described in the main text. The ^{1}H, ^{13}C, COSY, HSQC, and HMBC NMR spectra of the isolated product from the organic fraction were identical to those of the chemically synthesized β-deoxy-α-veratrylglycerone (Fig. S2A). The aqueous fraction was then injected into a Biotage 100g C18 reversed phase HPLC column and the unreacted β-epimer of GS-βVG (found to be GS-β(*S*)VG) was purified by chromatography as described previously.

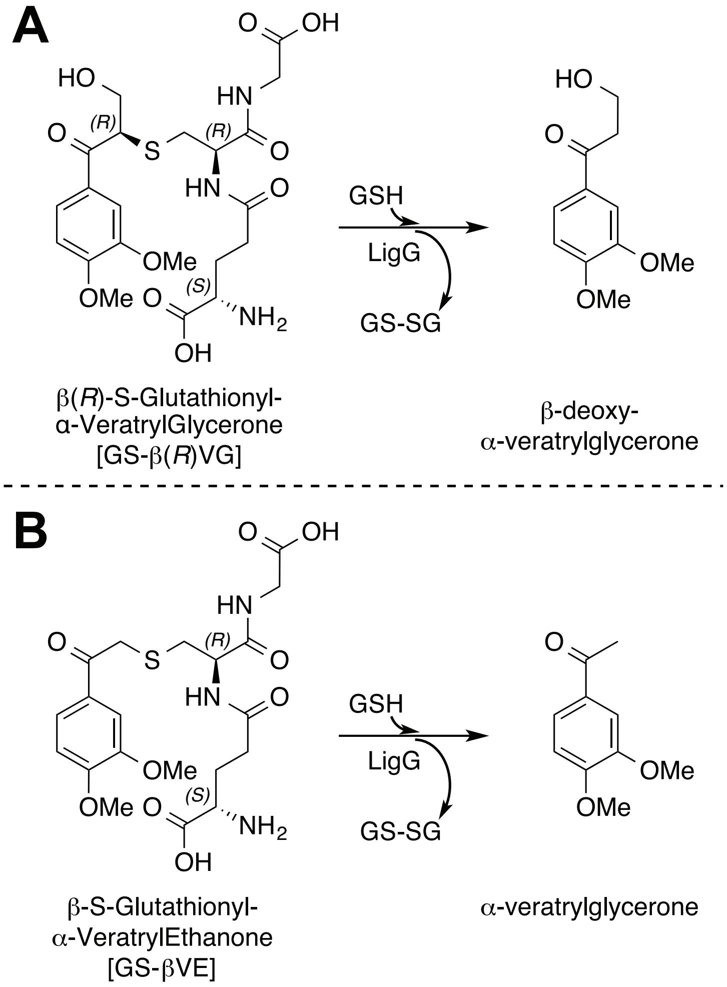

Fig. S2. Scheme for LigG-catalyzed synthesis of (**A**) β-deoxy-α-veratrylglycerone and (**B**) α-veratrylethanone.

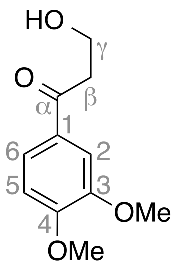

β-deoxy-α-veratrylglycerone [from LigG]:

^{1}H NMR (700 MHz, acetone-*d₆*) δ 7.67 (d, 1H, *J* = 8.4, 2.0 Hz, H6); 7.53 (d, 1H, *J* = 2.0 Hz, H2); 7.05 (d, 1H, *J* = 8.4 Hz, H5); 3.93 – 3.90 (m, 2H, $H\gamma_{a/b}$); 3.90 (s, 3H, 4-OMe); 3.87 (s, 3H, 3-OMe); 3.60 (t, 1H, *J* = 5.7 Hz, γ-OH); 3.16 (d, 2H, *J* = 6.2 Hz, $H\beta_{a/b}$)

^{13}C NMR (176 MHz, acetone-d_6) δ 198.2 (Cα); 154.5 (C4); 150.1 (C3); 131.3 (C1); 123.5 (C6); 111.4 (C5); 111.1 (C2); 58.6 (Cγ); 56.1 (4-OMe); 56.0 (3-OMe); 41.5 (Cβ).

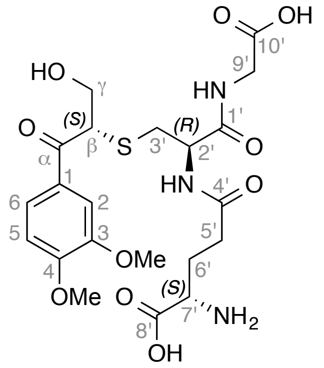

β(*S*)-S-glutathionyl-α-veratrylglycerone, GS-β(*S*)VG [not degraded by LigG]:
^{1}H NMR (700 MHz, D_2O) δ 7.69 (dd, 1H, J = 8.4, 2.0 Hz, H6); 7.48 (d, 1H, J = 2.0 Hz, H2); 7.03 (d, 1 H, J = 8.4 Hz, H5); 4.54 (dd, 1H, J = 7.9, 5.9 Hz, Hβ); 4.27 (dd, 1H, J = 8.8, 5.1 Hz, H2′); 3.98 (dd, 1H, J = 11.6, 7.9 Hz, $H\gamma_a$); 3.84 (s, 3H, 4-OMe); 3.82 - 3.77 (m, 1H, $H\gamma_b$); 3.81 (s, 3H, 3-OMe); 3.57 (d, 1H, J = 17.3 Hz, $H9'_a$); 3.50 (d, 1H, J = 17.3 Hz, $H9'_b$); 3.46 (t, 1H, J = 6.4 Hz, H7′); 2.95 (dd, 1H, J = 14.2, 5.1 Hz, $H3'_a$); 2.77 (dd, 1H, J = 14.2, 8.8 Hz, $H3'_b$); 2.26 – 2.14 (m, 2H, $H5'_{a/b}$); 1.88 – 1.79 (m, 2H, $H6'_{a/b}$).
^{13}C NMR (176 MHz, D_2O) δ 198.3 (Cα); 176.9 (C10′); 175.9 (C4′); 175.6 (C8′); 172.2 (C1′); 154.4 (C4); 149.0 (C3); 128.9 (C1); 125.3 (C6); 111.8 (C2); 111.8 (C5); 61.5 (Cγ); 56.7 (4-OMe); 56.5 (3-OMe); 55.1 (C7′); 53.7 (C2′); 48.5 (Cβ); 44.0 (C9′); 32.2 (C5′); 31.6 (C3′); 26.7 (C6′).

***In vitro* enzymatic synthesis of α-veratrylethanone from LigG (Fig. S2).** To 5 mL of the enzyme assay buffer, chemically synthesized GS-βVE (2.4 mg, 1.0 mM) was added and the buffer was re-adjusted to pH 7.5 using NaOH. Enzyme LigG (1.3 mg, 8.5 μM) was then added and the reaction was monitored and analyzed as described in the main text. The ^{1}H, ^{13}C, COSY, HSQC, and HMBC NMR spectra of the isolated product were identical to those from the chemically synthesized compound α-veratrylglycerone (Fig. S2B).

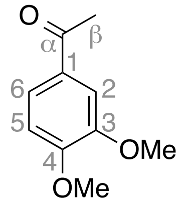

α-Veratrylethanone [from LigG]:
^{1}H NMR (700 MHz, acetone-d_6) δ 7.64 (dd, 1H, J = 8.4, 2.1 Hz, H6); 7.51 (d, 1H, J = 2.1 Hz, H2); 7.04 (d, 1H, J = 8.4 Hz, H5); 3.89 (s, 3H, 4-OMe); 3.86 (s, 3H, 3-OMe); 2.50 (s, 3H, $H\beta_{a/b/c}$).
^{13}C NMR (176 MHz, acetone-d_6) δ 196.5 (Cα); 154.4 (C4); 150.0 (C3); 131.3 (C1); 123.8 (C6); 111.3 (C5); 111.1 (C2); 56.1 (C4-OMe); 56.0 (3-OMe); 25.8 (Cβ).

SUPPLEMENTARY REFERENCES

1. Adler E & Eriksoo E (1955) Guaiacylglycerol and its β-guaiacyl ether. *Acta chemica Scandinavica* 9:341-342.
2. Landucci LL, Geddes SA, & Kirk TK (1981) Synthesis of C-14-labeled 3-methoxy-4-hydroxy-α-(2-methoxyphenoxy)-β-hydroxypropiophenone, a lignin model-compound. *Holzforschung* 35(2):67-70.
3. Gottlieb HE, Kotlyar V, & Nudelman A (1997) NMR chemical shifts of common laboratory solvents as trace impurities. *Journal of Organic Chemistry* 62(21):7512-7515.
4. Fulmer GR*, et al.* (2010) NMR chemical shifts of trace impurities: Common laboratory solvents, organics, and gases in deuterated solvents relevant to the organometallic chemist. *Organometallics* 29(9):2176-2179.

SUPPLEMENTARY INFORMATION - ^{1}H AND ^{13}C NMR SPECTRA OF MODEL COMPOUNDS AND REACTION PRODUCTS

**COMPOUND:**	**Source:**[a]	**Solvent:**[b]	**Page:**
α-Veratrylethanone	Sigma-Aldrich	acetone-d_6	S - 20
α-Veratrylethanone	LigG	acetone-d_6	S - 22
β-deoxy-α-veratrylglycerone	synthetic	acetone-d_6	S - 24
β-deoxy-α-veratrylglycerone	LigG	acetone-d_6	S - 26
β-S-glutathionyl-α-veratrylethanone, GS-βVE	synthetic	D_2O	S - 28
β-S-glutathionyl-α-veratrylethanone, GS-βVE	synthetic	90/10 H_2O/ D_2O	S - 30
β-S-glutathionyl-α-veratrylethanone, GS-βVE	LigE	D_2O	S - 32
β-S-glutathionyl-α-veratrylethanone, GS-βVE	LigP	D_2O	S - 34
β-S-glutathionyl-α-veratrylethanone, GS-βVE	LigF	D_2O	S - 36
β-S-glutathionyl-α-veratrylglycerone, GS-β(*S*)VG and GS-β(*R*)VG	synthetic	D_2O	S - 38
β(*S*)-S-glutathionyl-α-veratrylglycerone, GS-β(*S*)VG	LigE	D_2O	S - 40
β(*S*)-S-glutathionyl-α-veratrylglycerone, GS-β(*S*)VG	LigP	D_2O	S - 42
β(*S*)-S-glutathionyl-α-veratrylglycerone, GS-β(*S*)VG	LigG residual	D_2O	S - 44
β(*R*)-S-glutathionyl-α-veratrylglycerone, GS-β(*R*)VG	LigF	D_2O	S - 46
γ,γ-di-OMe-β(*S*)-S-[Me N-Ac-cysteinyl]-α-veratrylpropanone, CS-β(*S*)VP	synthetic	D_2O	S - 48
γ,γ-di-OMe-β(*S*)-S-[Me N-Ac-cysteinyl]-α-veratrylpropanone, CS-β(*S*)VP	synthetic	acetone-d_6	S - 50
γ,γ-di-OMe-β(*R*)-S-[Me N-Ac-cysteinyl]-α-veratrylpropanone, CS-β(*R*)VP	synthetic	D_2O	S - 52
γ,γ-di-OMe-β(*R*)-S-[Me N-Ac-cysteinyl]-α-veratrylpropanone, CS-β(*R*)VP	synthetic	acetone-d_6	S - 54

a Compounds were obtained from either commercial suppliers (Sigma-Aldrich), organic synthesis (synthetic), products of enzymatic reactions (LigE, LigP, LigF, or LigG), or unreacted substrates of enzymatic reactions (LigG residual).

b ^{1}H and ^{13}C NMR spectra were analyzed in either acetone-d_6, D_2O, or 90%/10% H_2O/D_2O as NMR solvents.

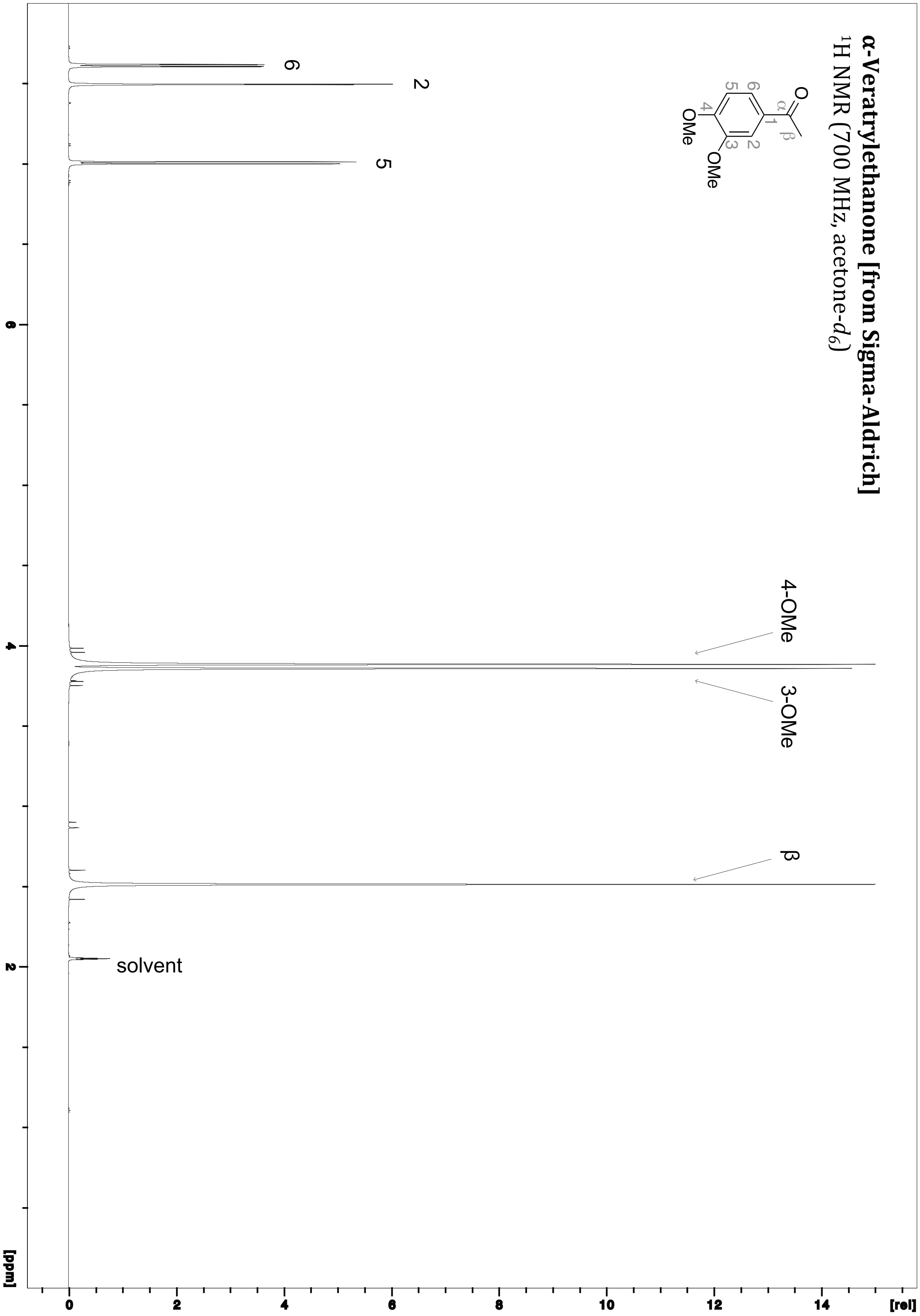
α-Veratrylethanone [from Sigma-Aldrich]
¹H NMR (700 MHz, acetone-d6)
O
α
β
1
2
3
4
5
6
OMe
OMe
6
2
5
4-OMe
3-OMe
β
solvent
8
6
4
2
[ppm]
0
2
4
6
8
10
12
14
[rel]

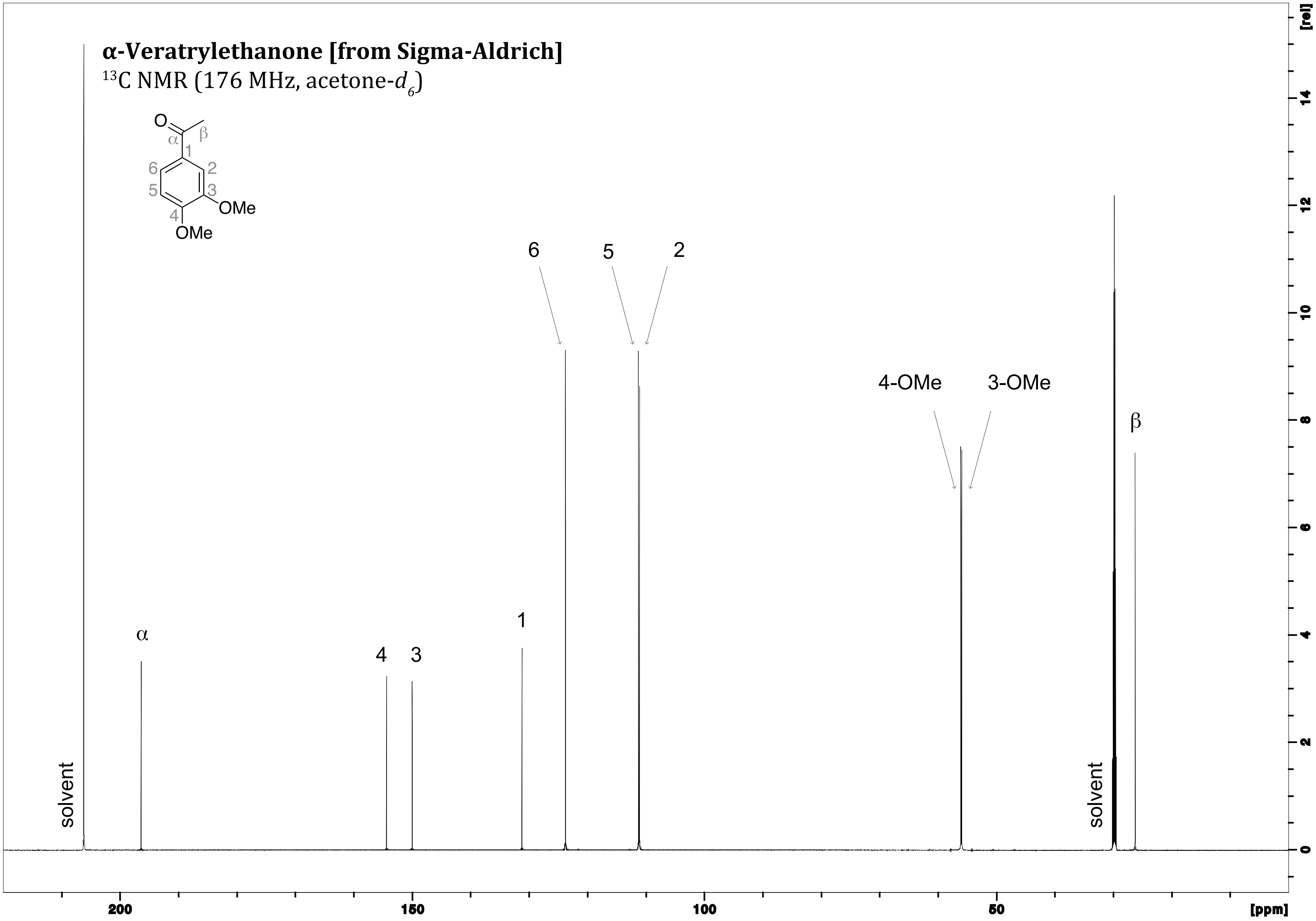
α-Veratrylethanone [from Sigma-Aldrich]
^{13}C NMR (176 MHz, acetone-d_6)
O
α
β
1
2
3
4
5
6
OMe
OMe
solvent
α
4
3
1
6
5
2
4-OMe
3-OMe
solvent
β
200
150
100
50
[ppm]
0
2
4
6
8
10
12
14
[rel]

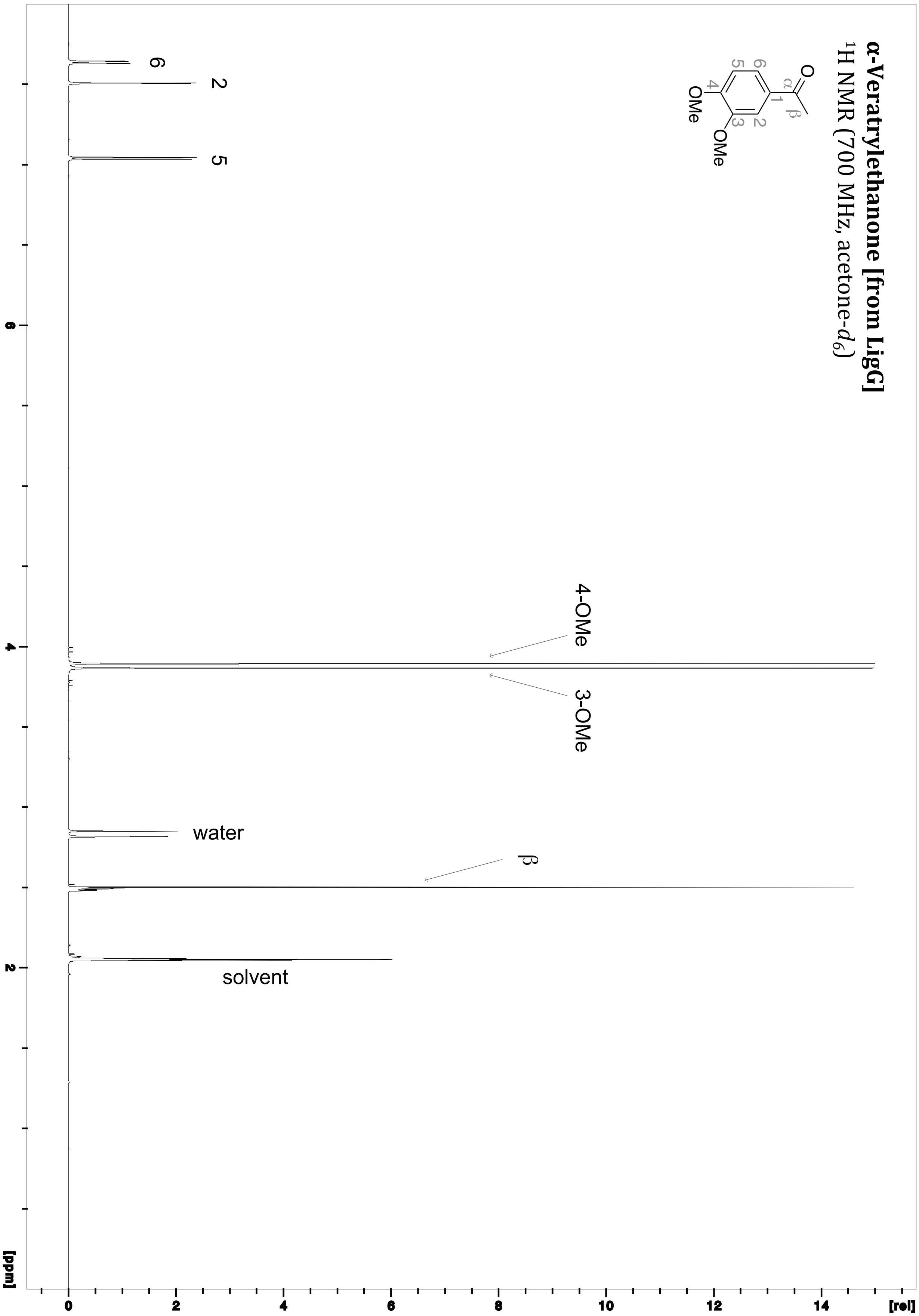

α-Veratrylethanone [from LigG]
¹H NMR (700 MHz, acetone-d6)
O
α
β
1
2
3
4
5
6
OMe
OMe
6
2
5
4-OMe
3-OMe
water
β
solvent
[ppm]
[rel]

α-Veratrylethanone [from LigG]

^{13}C NMR (176 MHz, acetone-d_6)

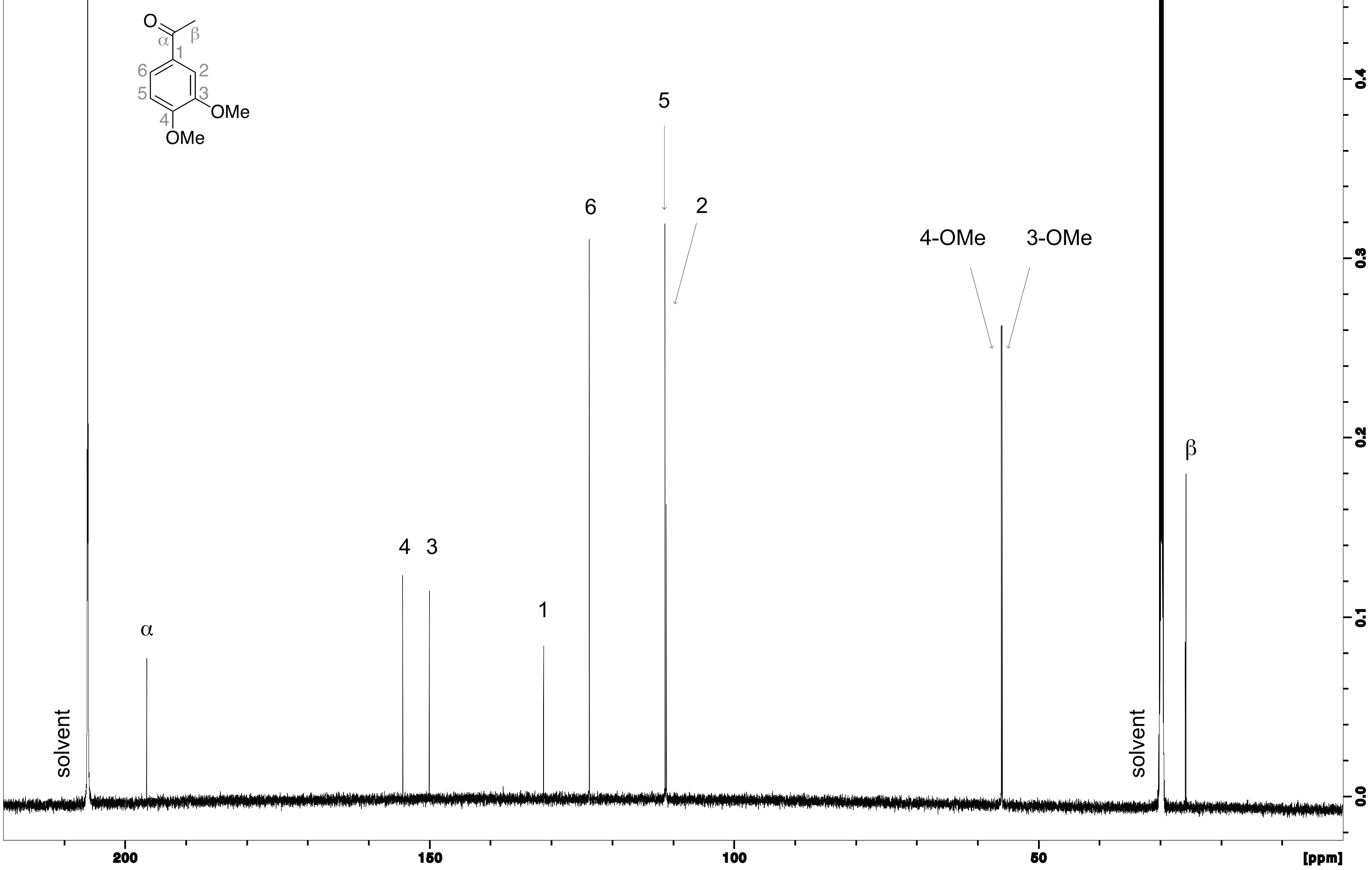

β-Deoxy-α-veratrylglycerone [synthetic]

^{1}H NMR (700 MHz, acetone-d_6)

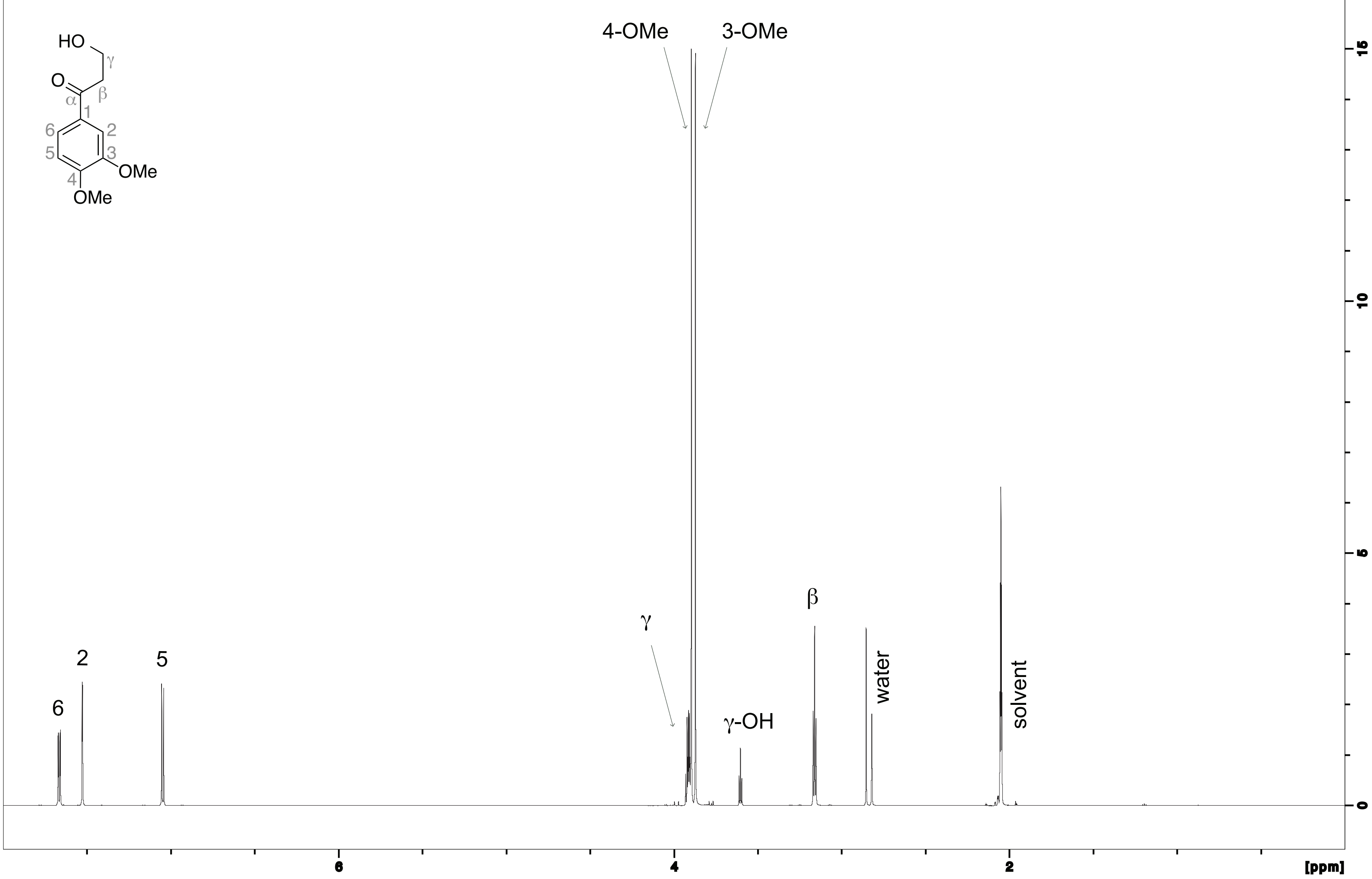

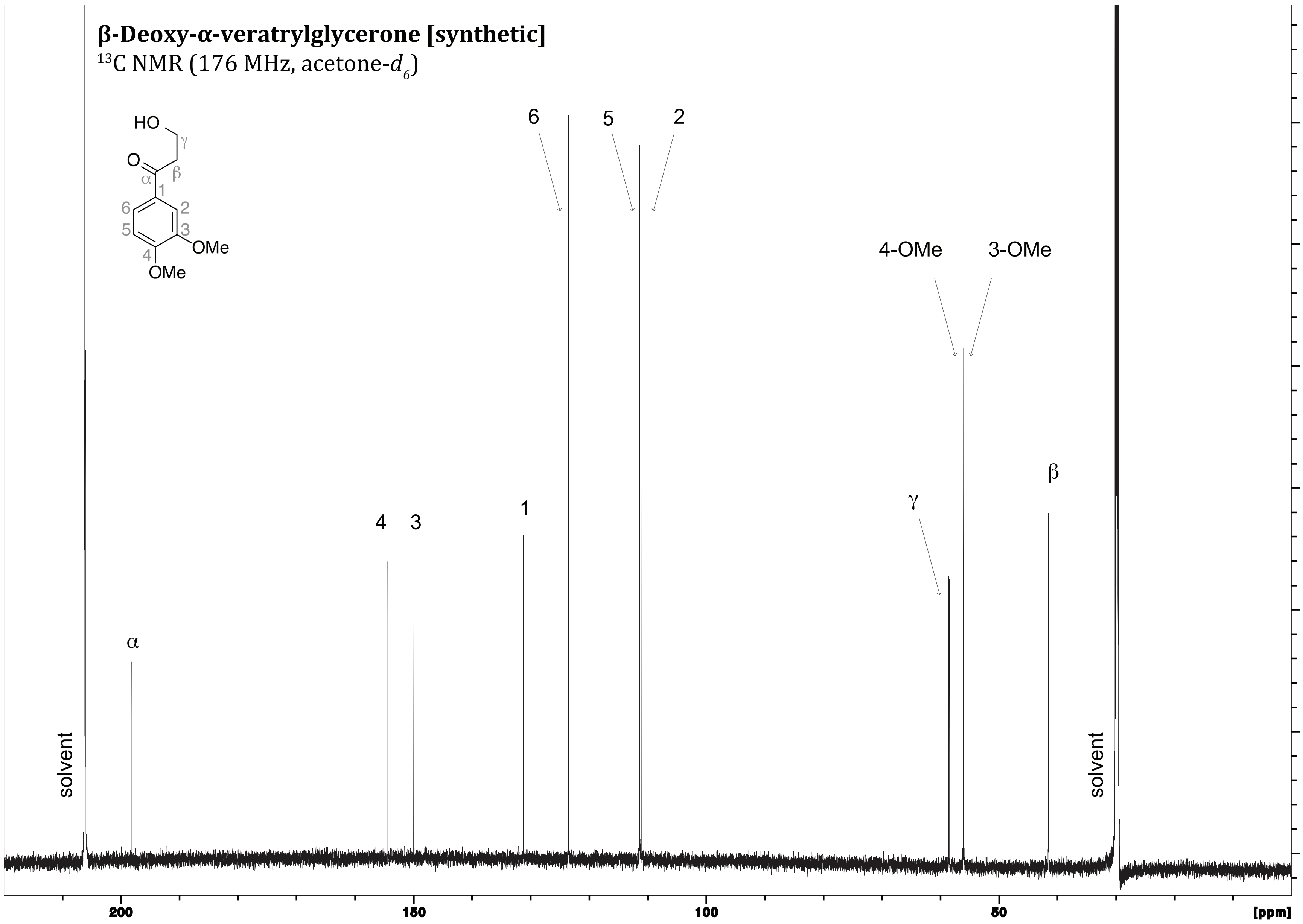
β-Deoxy-α-veratrylglycerone [synthetic]
^{13}C NMR (176 MHz, acetone-d_6)
HO
γ
O
β
α
1
2
3
4
5
6
OMe
OMe
solvent
α
4
3
1
6
5
2
4-OMe
3-OMe
γ
β
solvent
200
150
100
50
[ppm]
0.00
0.05
0.10
0.15
0.20
0.25
0.30
[rel]

β-deoxy-α-veratrylglycerone [from LigG]

^{1}H NMR (700 MHz, acetone-d_6)

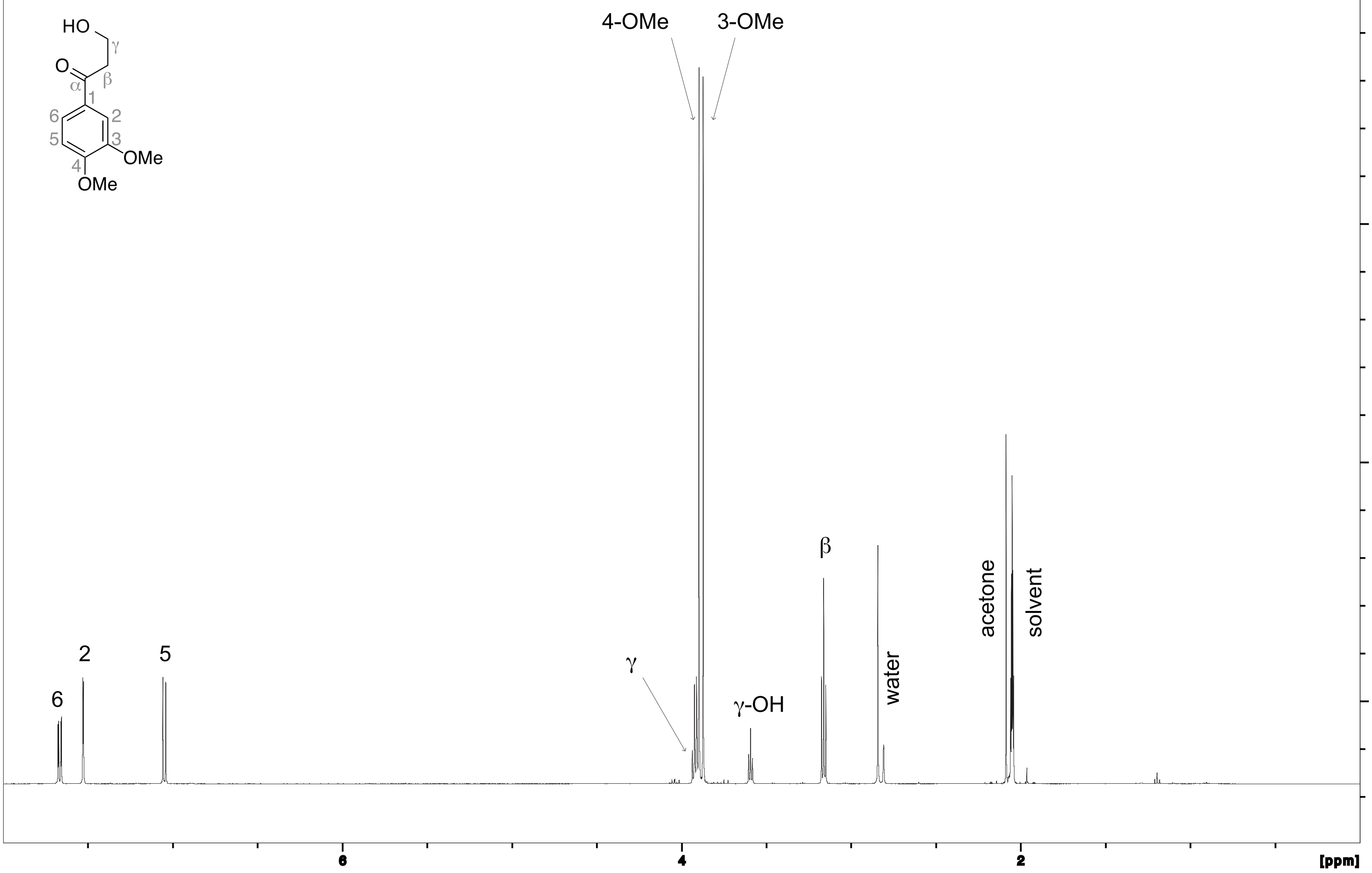

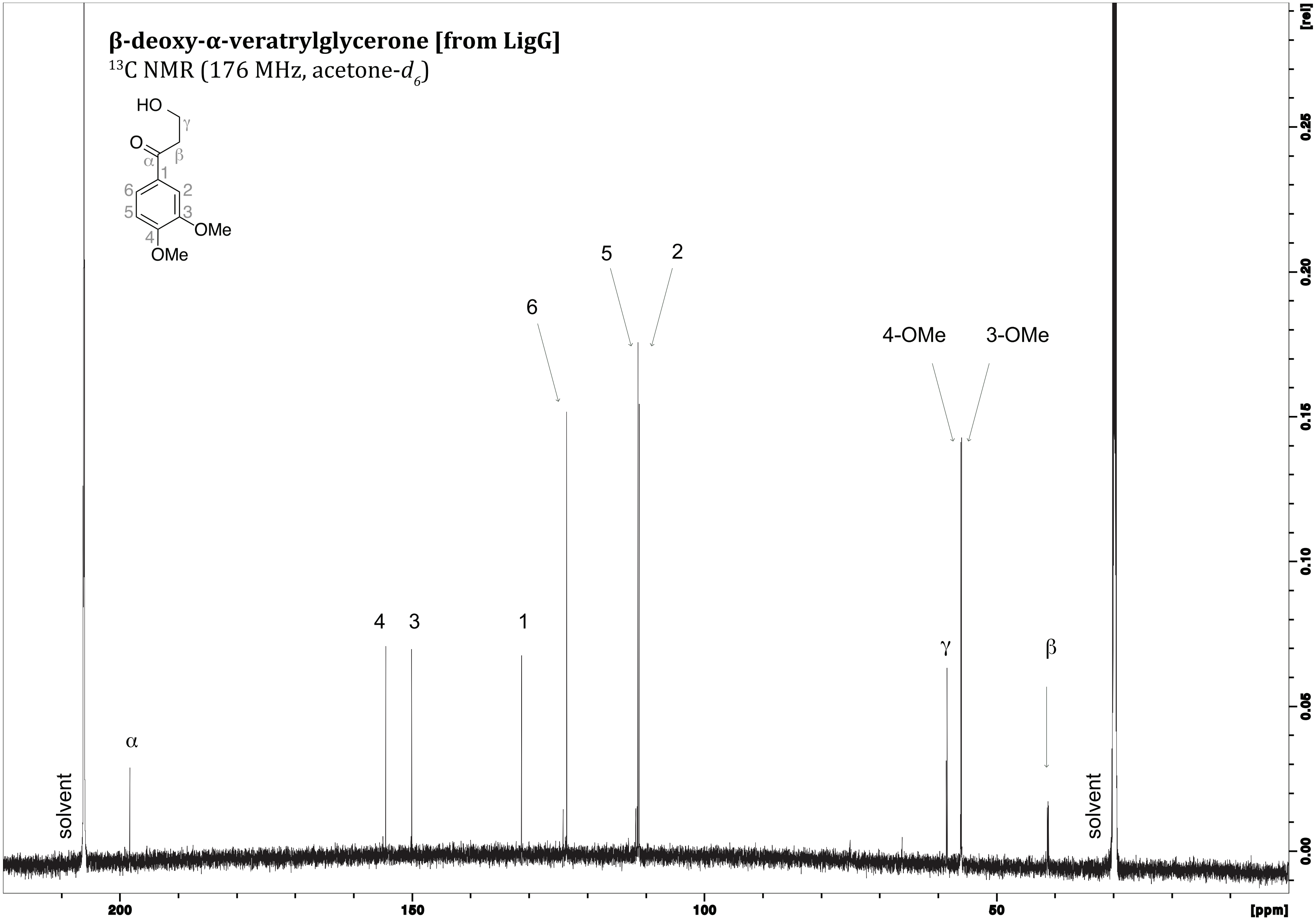
β-deoxy-α-veratrylglycerone [from LigG]
13C NMR (176 MHz, acetone-d6)
HO
O
OMe
OMe
γ
β
α
1
2
3
4
5
6
solvent
α
4
3
1
6
5
2
4-OMe
3-OMe
γ
β
solvent
200
150
100
50
[ppm]
0.00
0.05
0.10
0.15
0.20
0.25
[rel]

β-S-glutathionyl-α-veratrylethanone, GS-βVE [synthetic]

^1H NMR (700 MHz, D_2O)

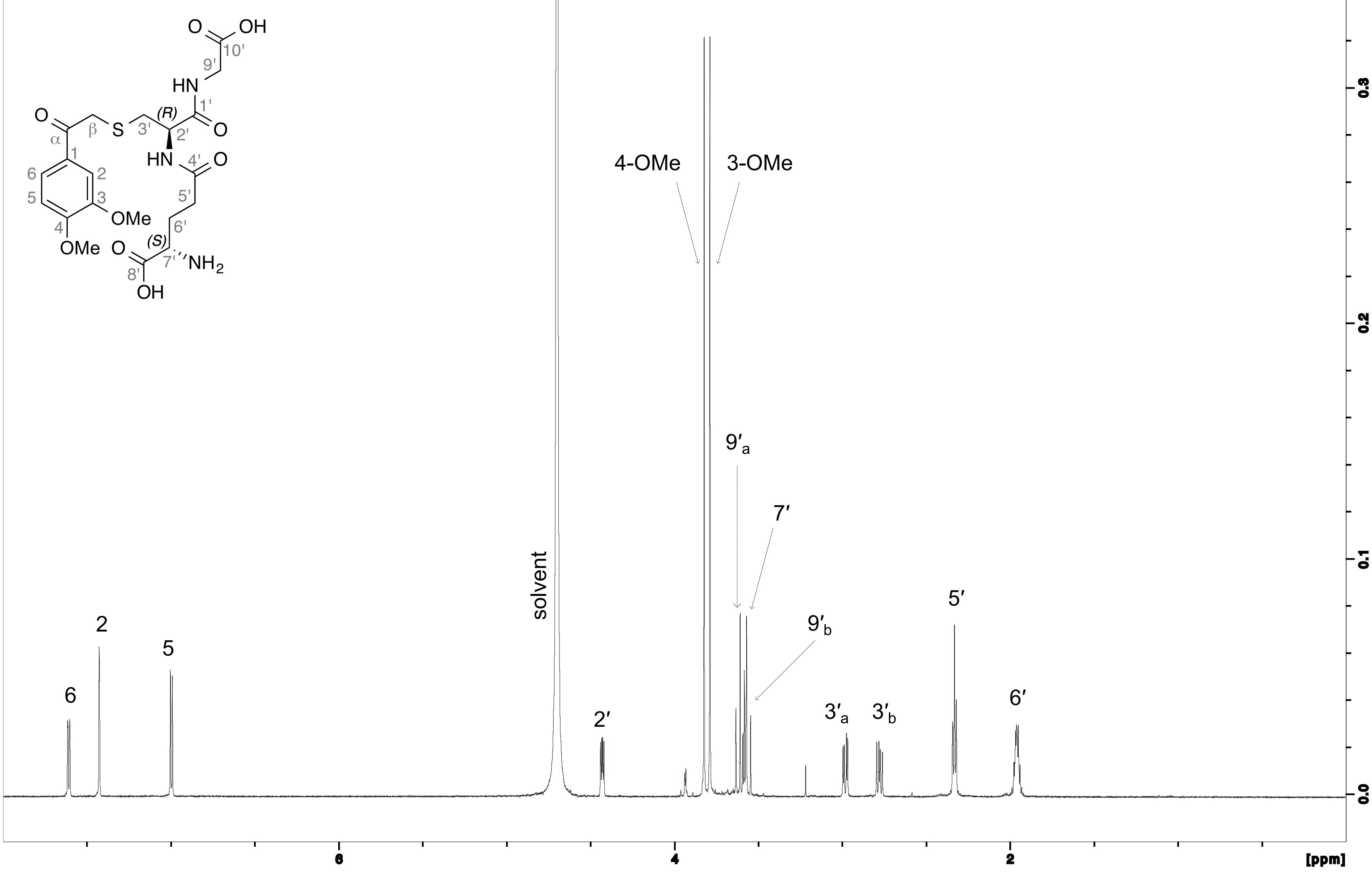

β-S-glutathionyl-α-veratrylethanone, GS-βVE [synthetic]

^{13}C NMR (176 MHz, D_2O)

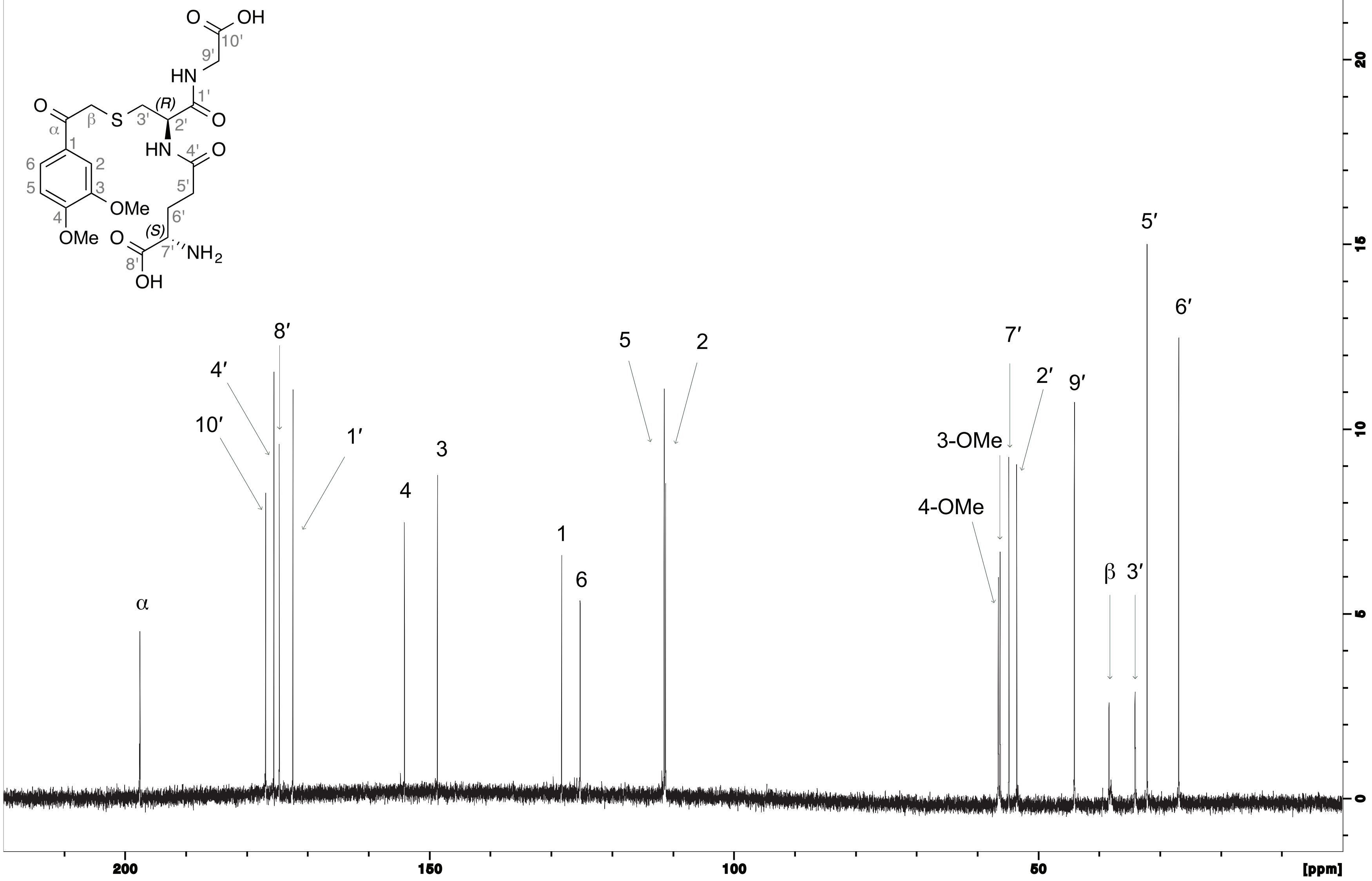

β-S-glutathionyl-α-veratrylethanone, GS-βVE [synthetic]

^{1}H NMR (700 MHz, 90% H_2O/10% D_2O)

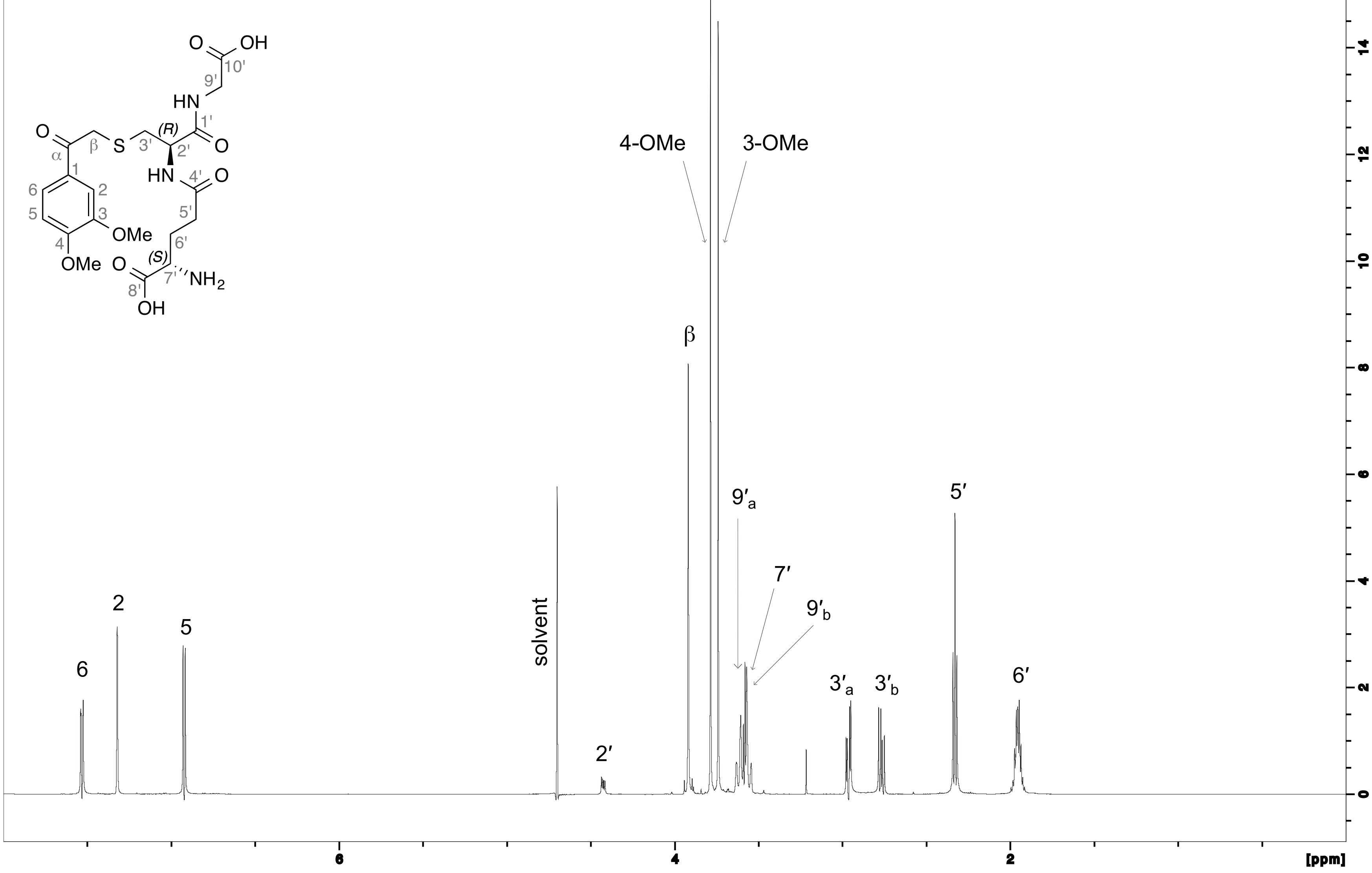

β-S-glutathionyl-α-veratrylethanone, GS-βVE [synthetic]

^{13}C NMR (176 MHz, 90% H_2O/10% D_2O)

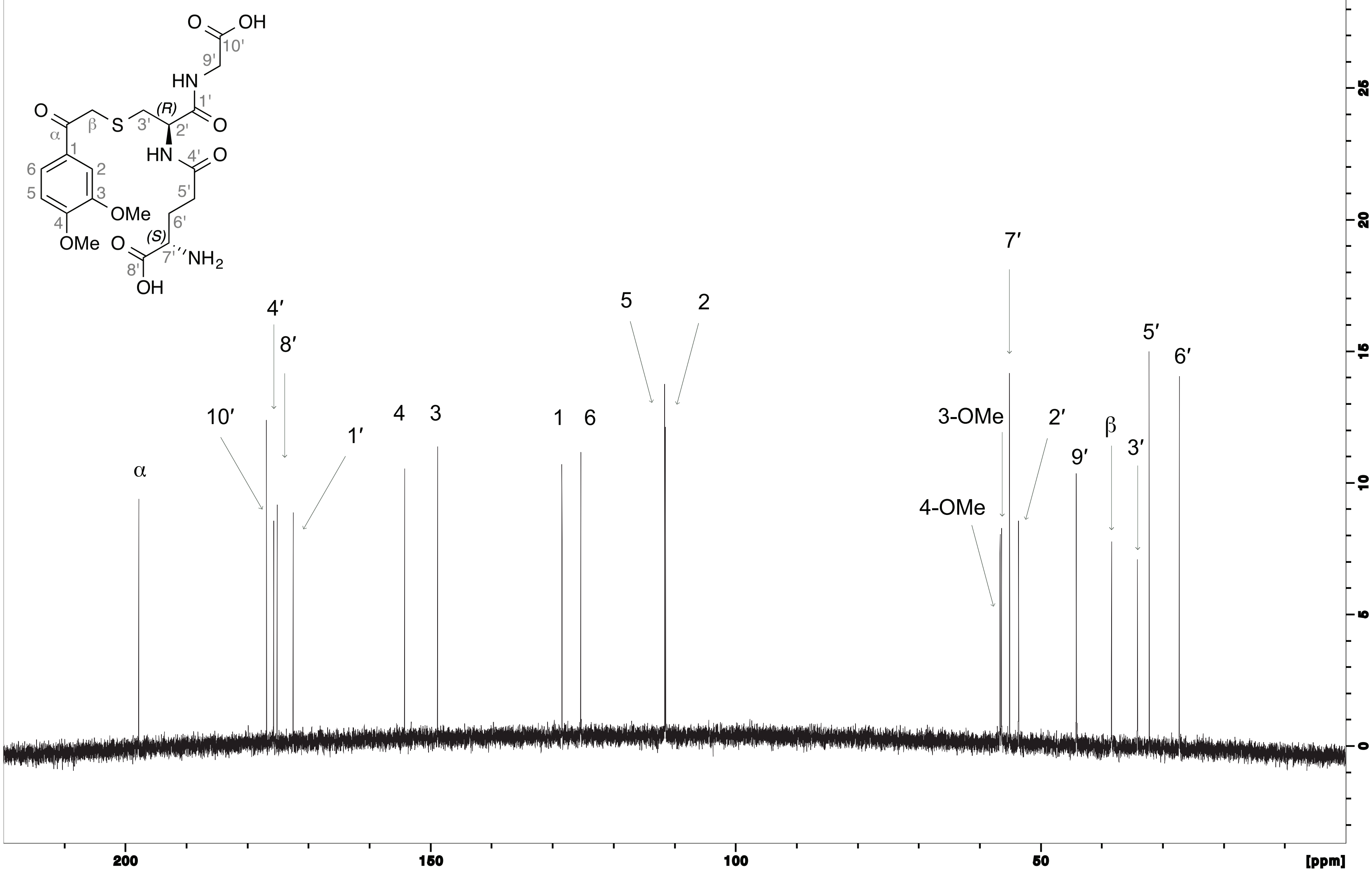

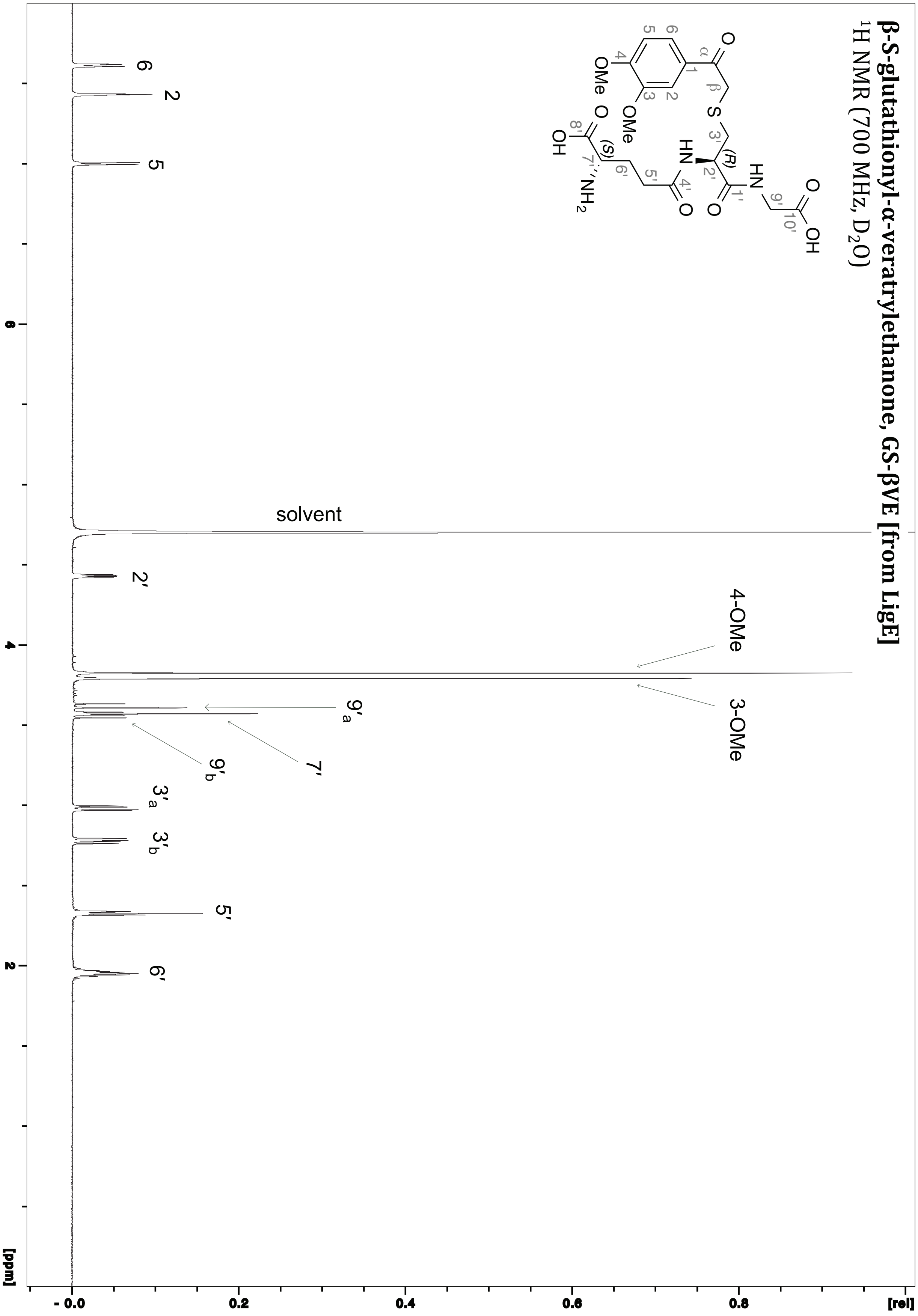

β-S-glutathionyl-α-veratrylethanone, GS-βVE [from LigE]
¹H NMR (700 MHz, D₂O)
solvent
4-OMe
3-OMe
9′a
9′b
7′
2′
3′a
3′b
5′
6′
6
2
5
[ppm]
[rel]

β-S-glutathionyl-α-veratrylethanone, GS-βVE [from LigE]
^{13}C NMR (176 MHz, D_2O)

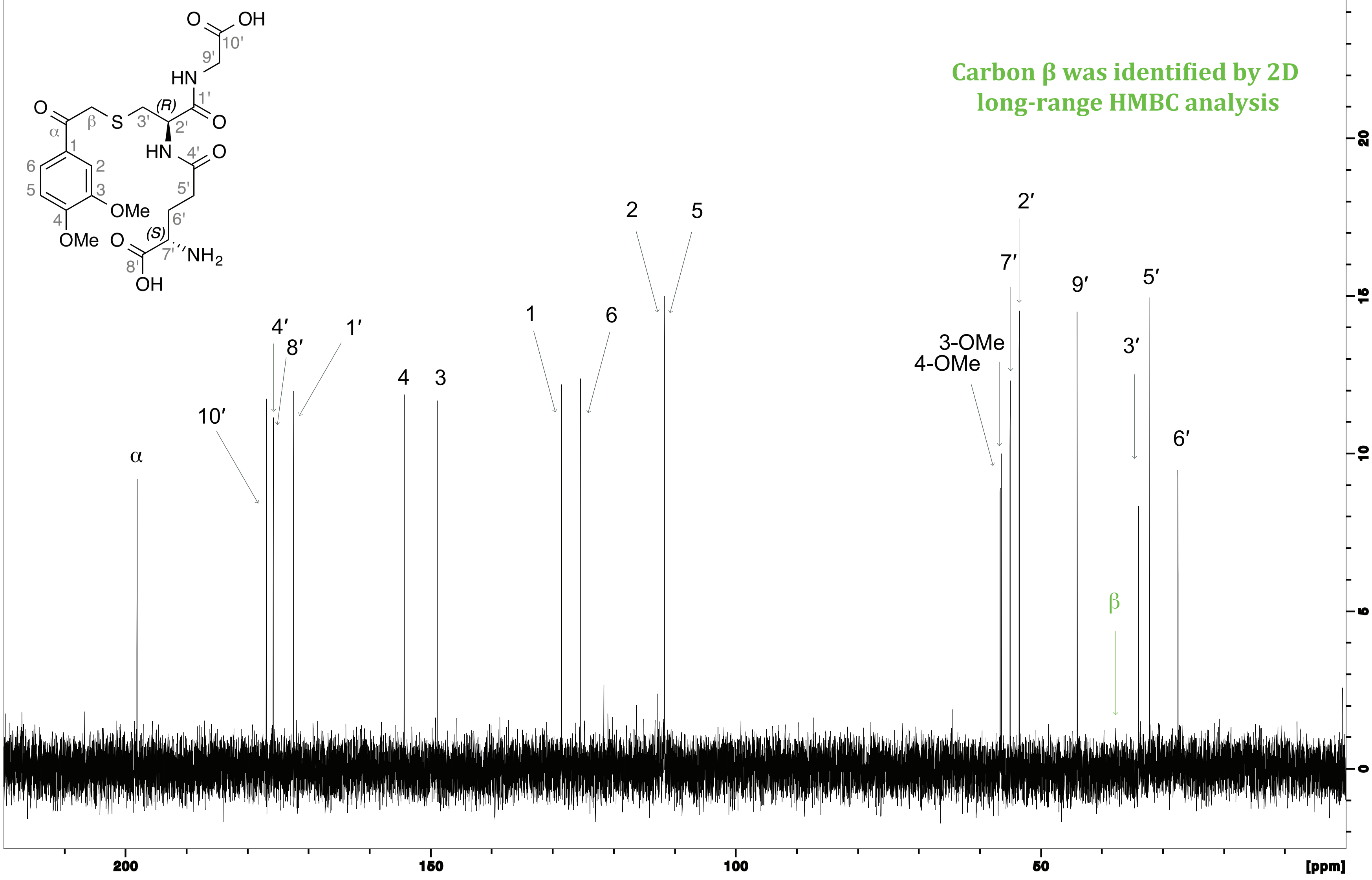

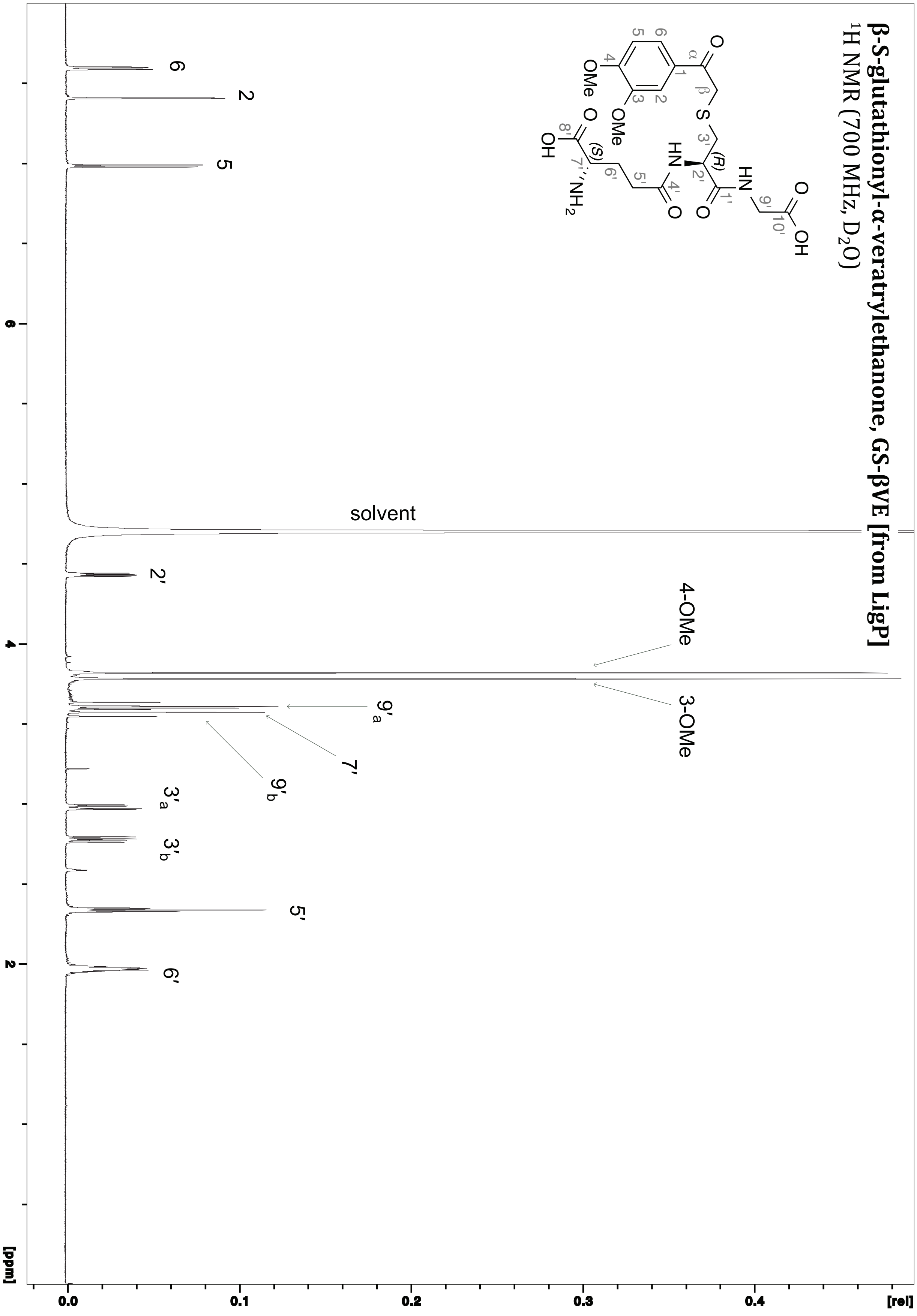

β-S-glutathionyl-α-veratrylethanone, GS-βVE [from LigP]
¹H NMR (700 MHz, D₂O)
6
2
5
solvent
2'
4-OMe
3-OMe
9'a
7'
9'b
3'a
3'b
5'
6'
[ppm]
[rel]

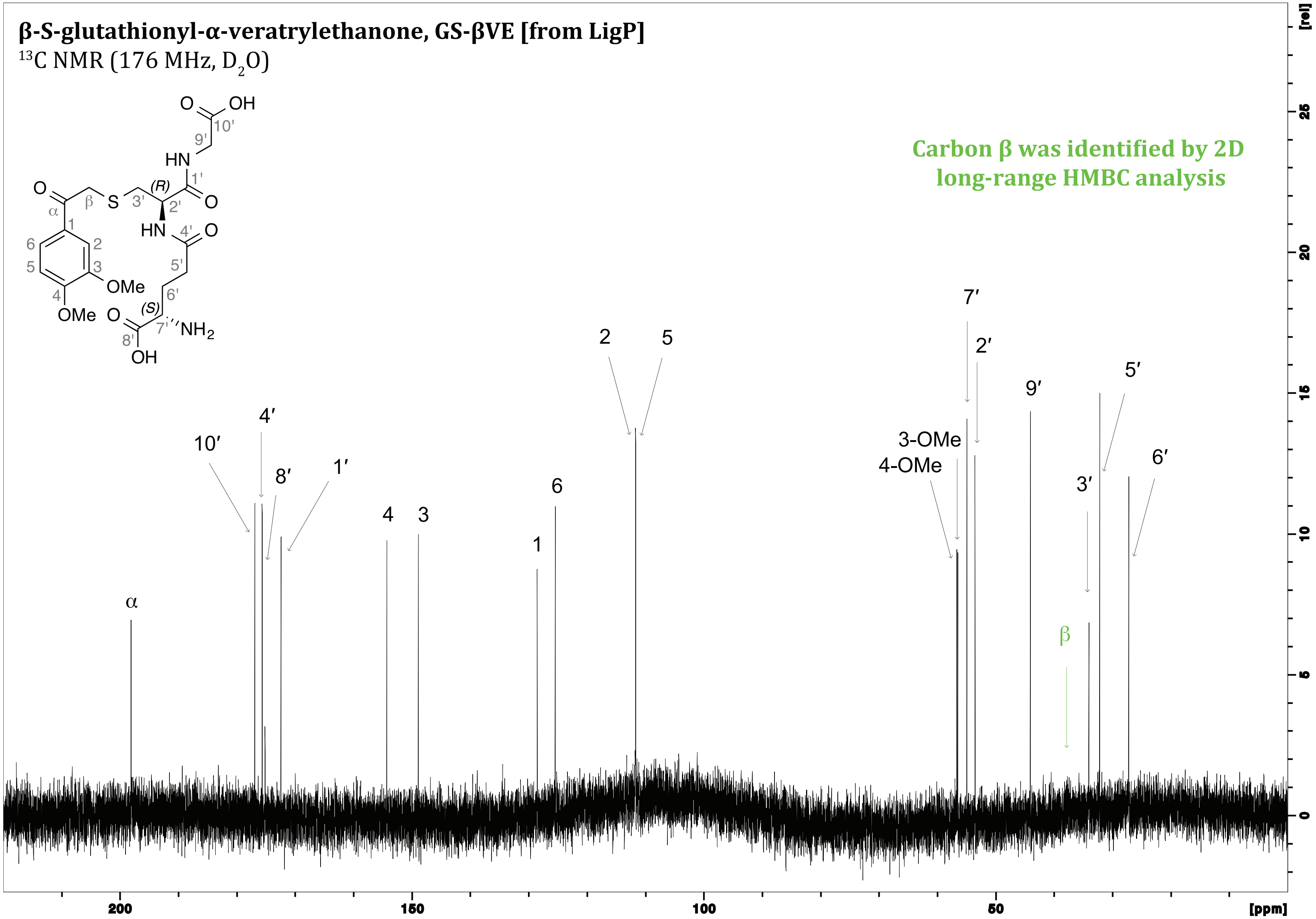

β-S-glutathionyl-α-veratrylethanone, GS-βVE [from LigP]
13C NMR (176 MHz, D2O)
Carbon β was identified by 2D long-range HMBC analysis
α
10′
4′
8′
1′
4
3
1
6
2
5
3-OMe
4-OMe
7′
2′
9′
β
3′
5′
6′
[ppm]
[rel]

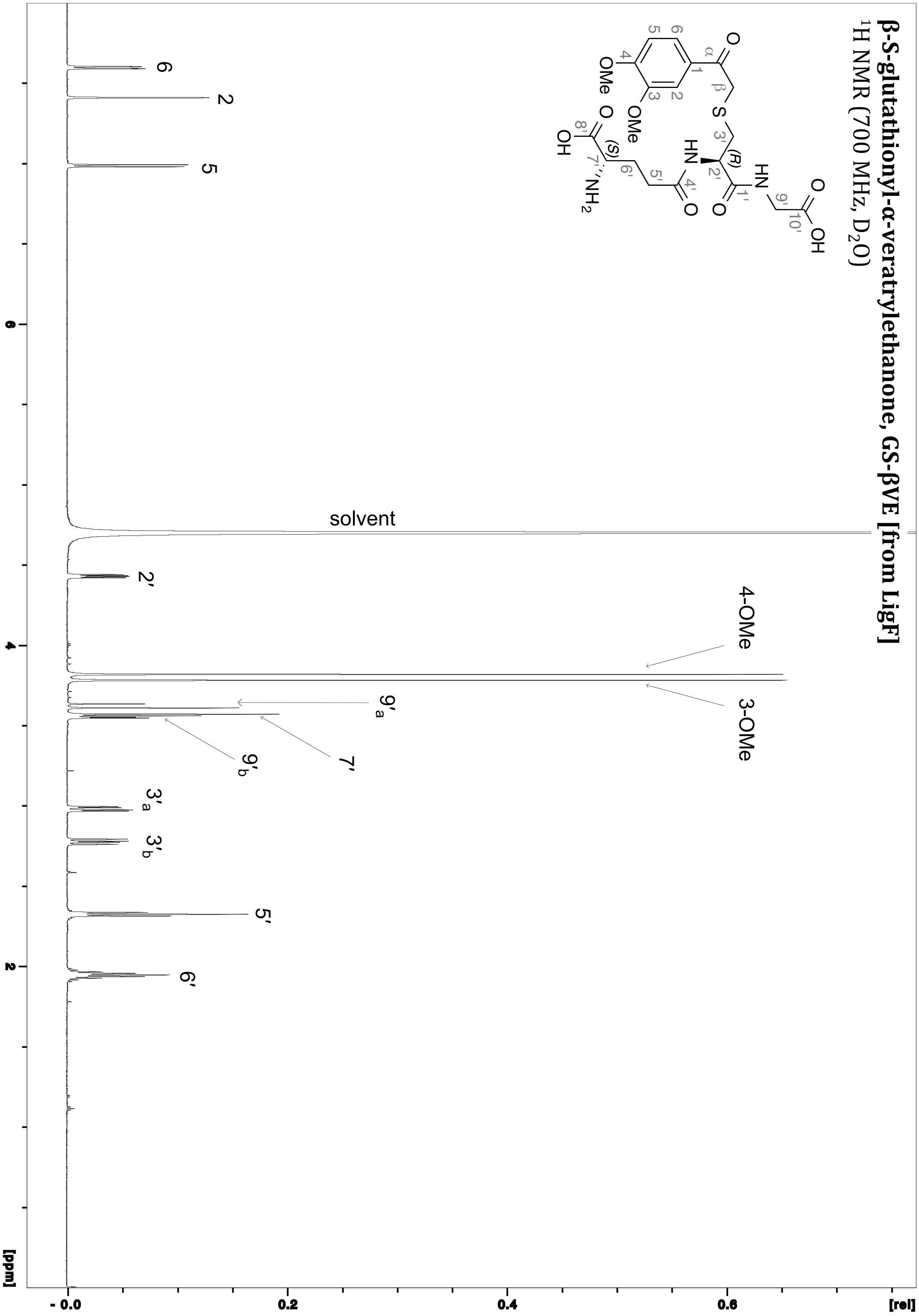

β-S-glutathionyl-α-veratrylethanone, GS-βVE [from LigF]
1H NMR (700 MHz, D2O)
solvent
4-OMe
3-OMe
9'a
9'b
7'
2'
3'a
3'b
5'
6'
6
2
5
[ppm]
[rel]
- 0.0
0.2
0.4
0.6
6
4
2

β-S-glutathionyl-α-veratrylethanone, GS-βVE [from LigF]

^{13}C NMR (176 MHz, D_2O)

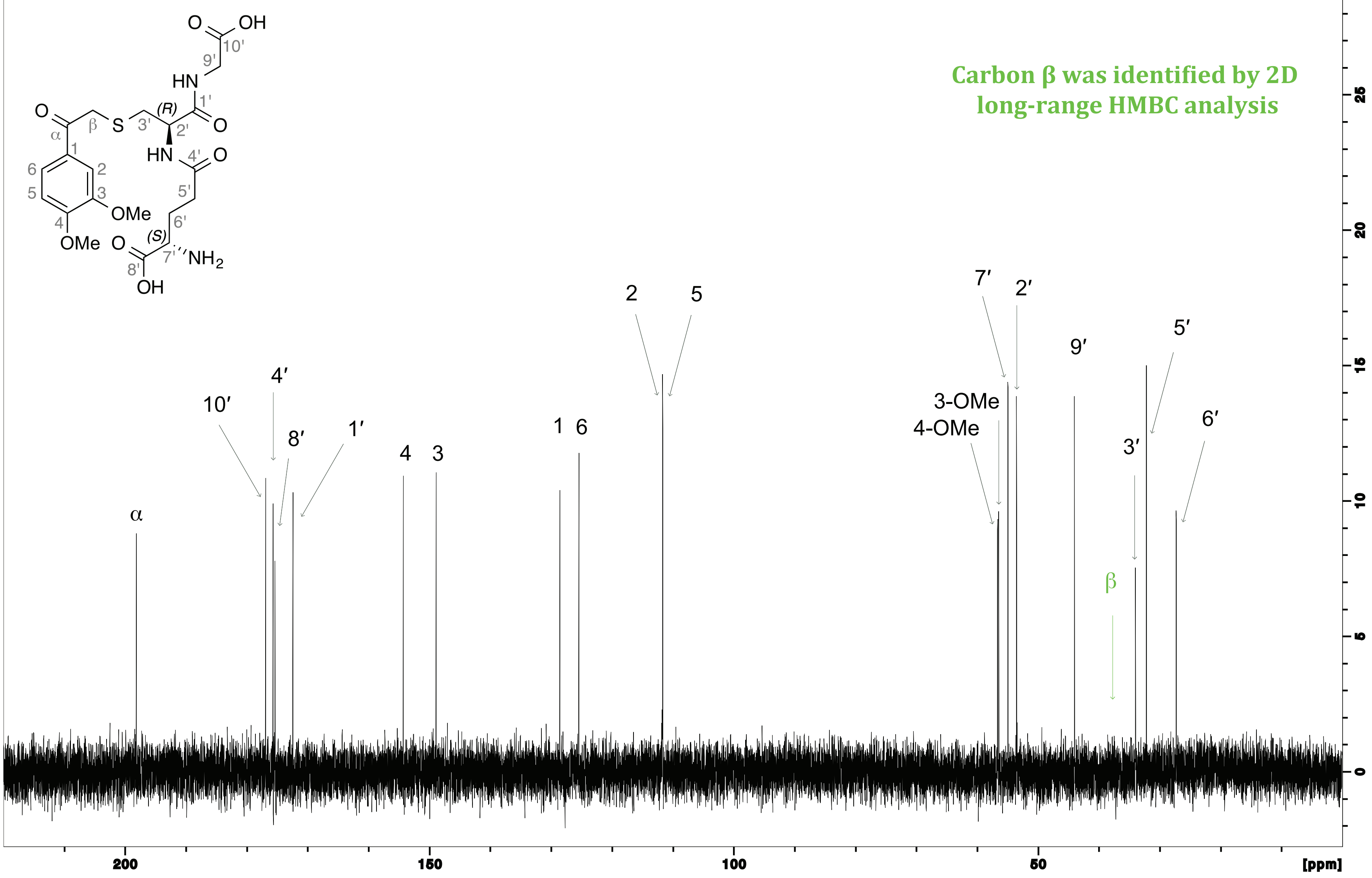

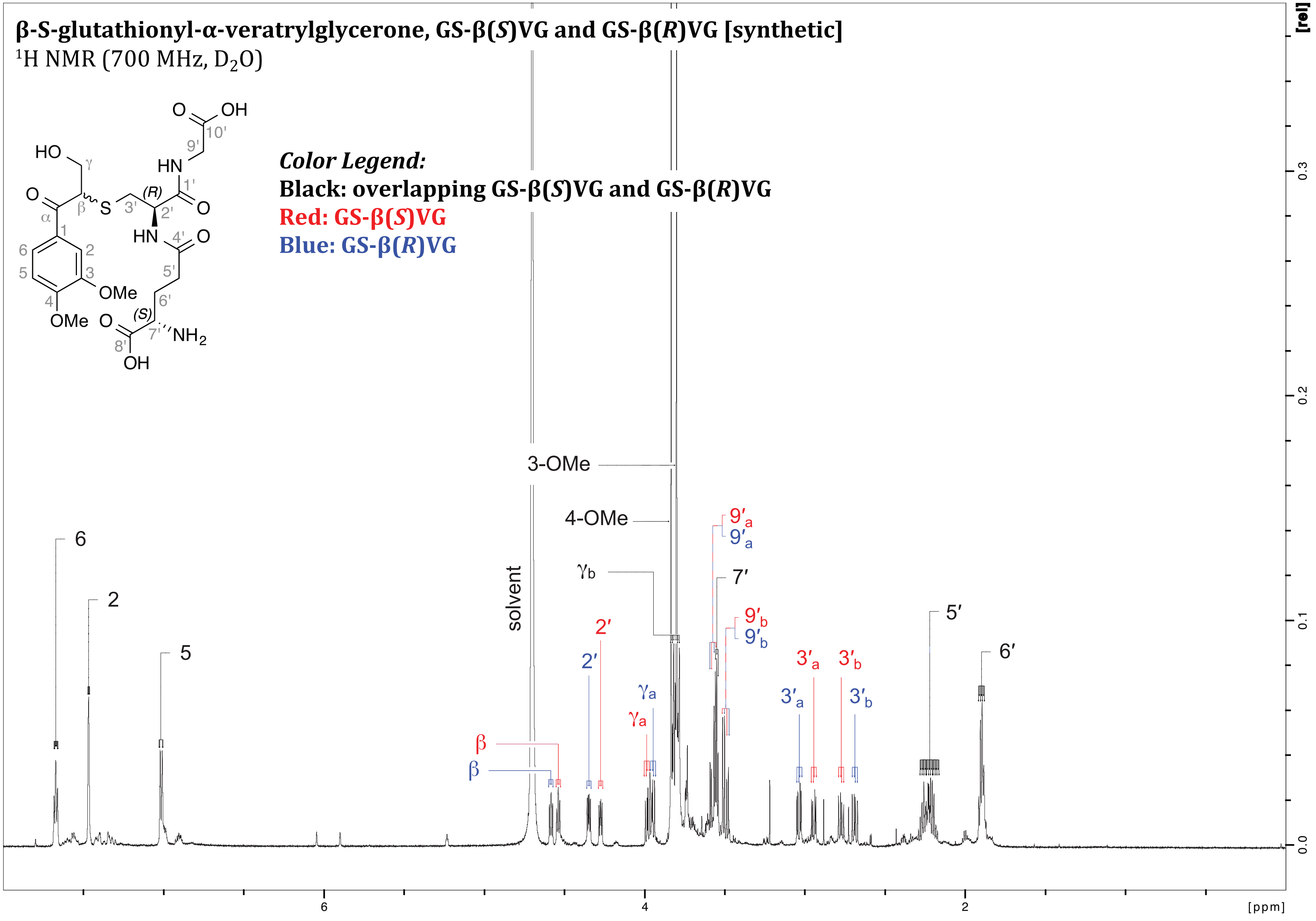
β-S-glutathionyl-α-veratrylglycerone, GS-β(S)VG and GS-β(R)VG [synthetic]
¹H NMR (700 MHz, D₂O)
Color Legend:
Black: overlapping GS-β(S)VG and GS-β(R)VG
Red: GS-β(S)VG
Blue: GS-β(R)VG
HO
O
OH
HN
S
(R)
(S)
OMe
NH₂
6
2
5
solvent
3-OMe
4-OMe
γb
γa
β
2′
7′
9′a
9′b
3′a
3′b
5′
6′
[ppm]
[rel]

β-S-glutathionyl-α-veratrylglycerone, GS-β(*S*)VG and GS-β(*R*)VG [synthetic]

^{13}C NMR (176 MHz, D_2O)

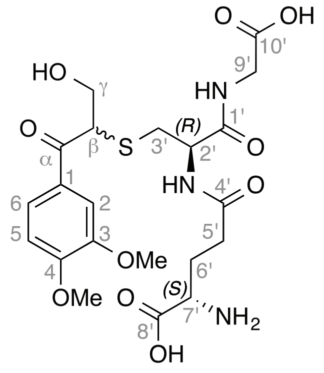

Color Legend:

Black: overlapping GS-β(*S*)VG and GS-β(*R*)VG

Red: GS-β(*S*)VG

Blue: GS-β(*R*)VG

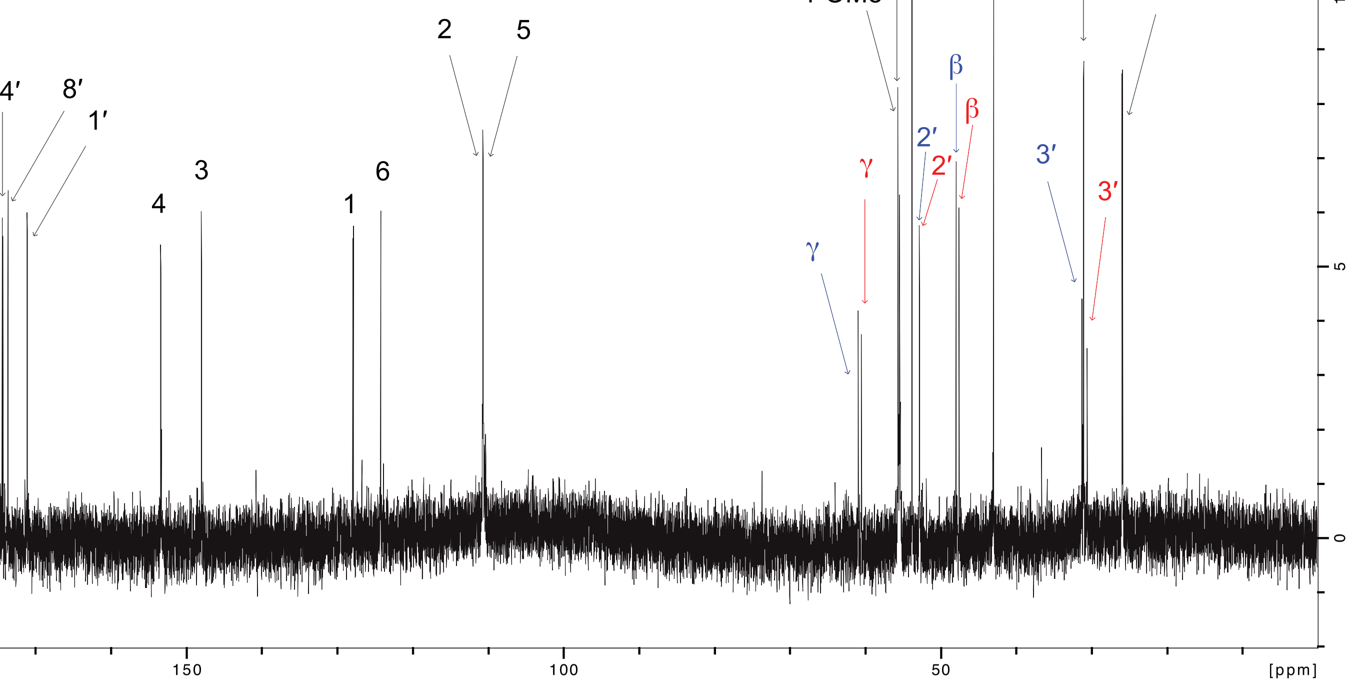

β(*S*)-*S*-glutathionyl-α-veratrylglycerone, GS-β(*S*)VG [from LigE]

^{1}H NMR (700 MHz, D_2O)

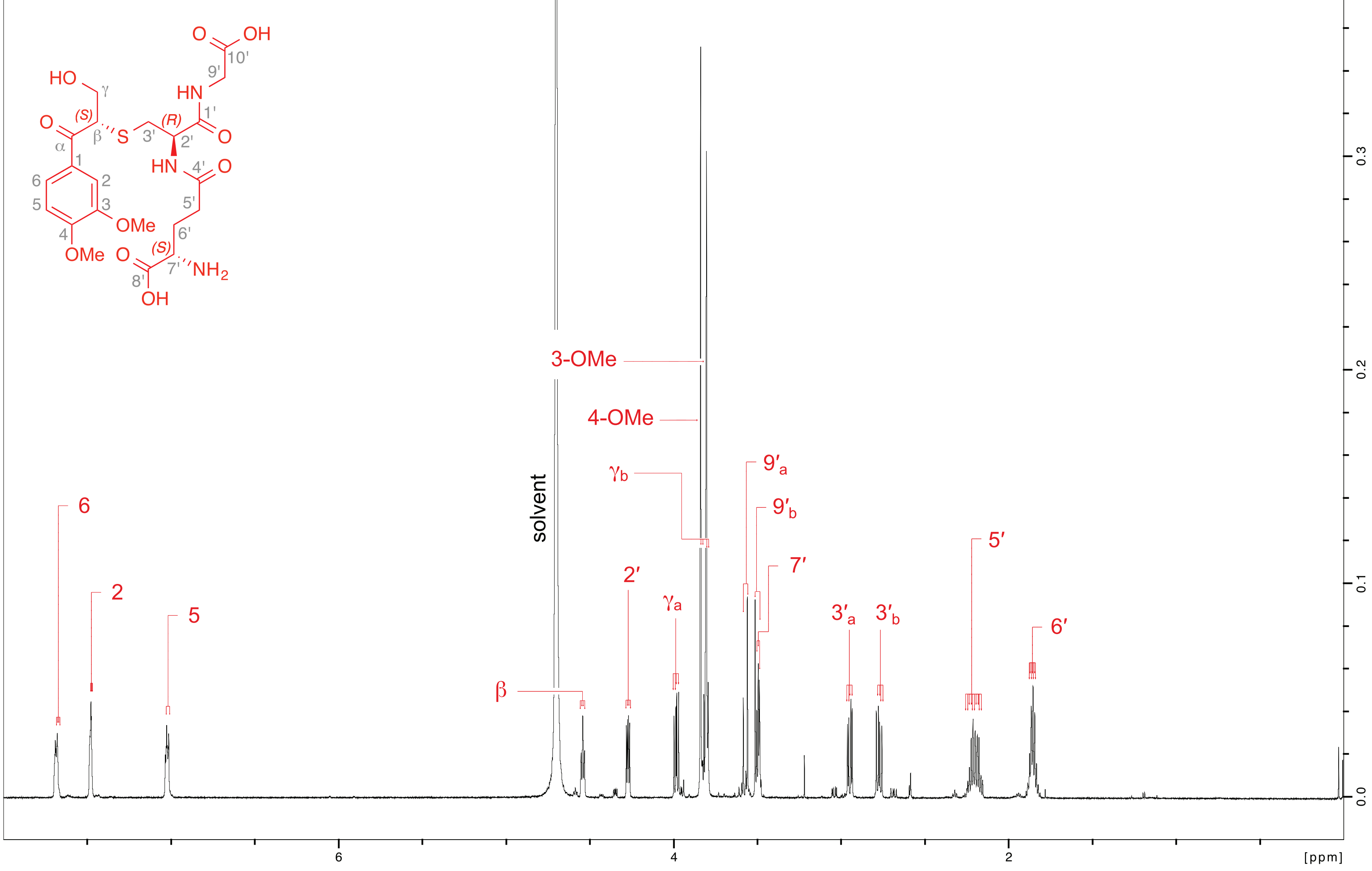

β(*S*)-S-glutathionyl-α-veratrylglycerone, GS-β(*S*)VG [from LigE]

^{13}C NMR (176 MHz, D_2O)

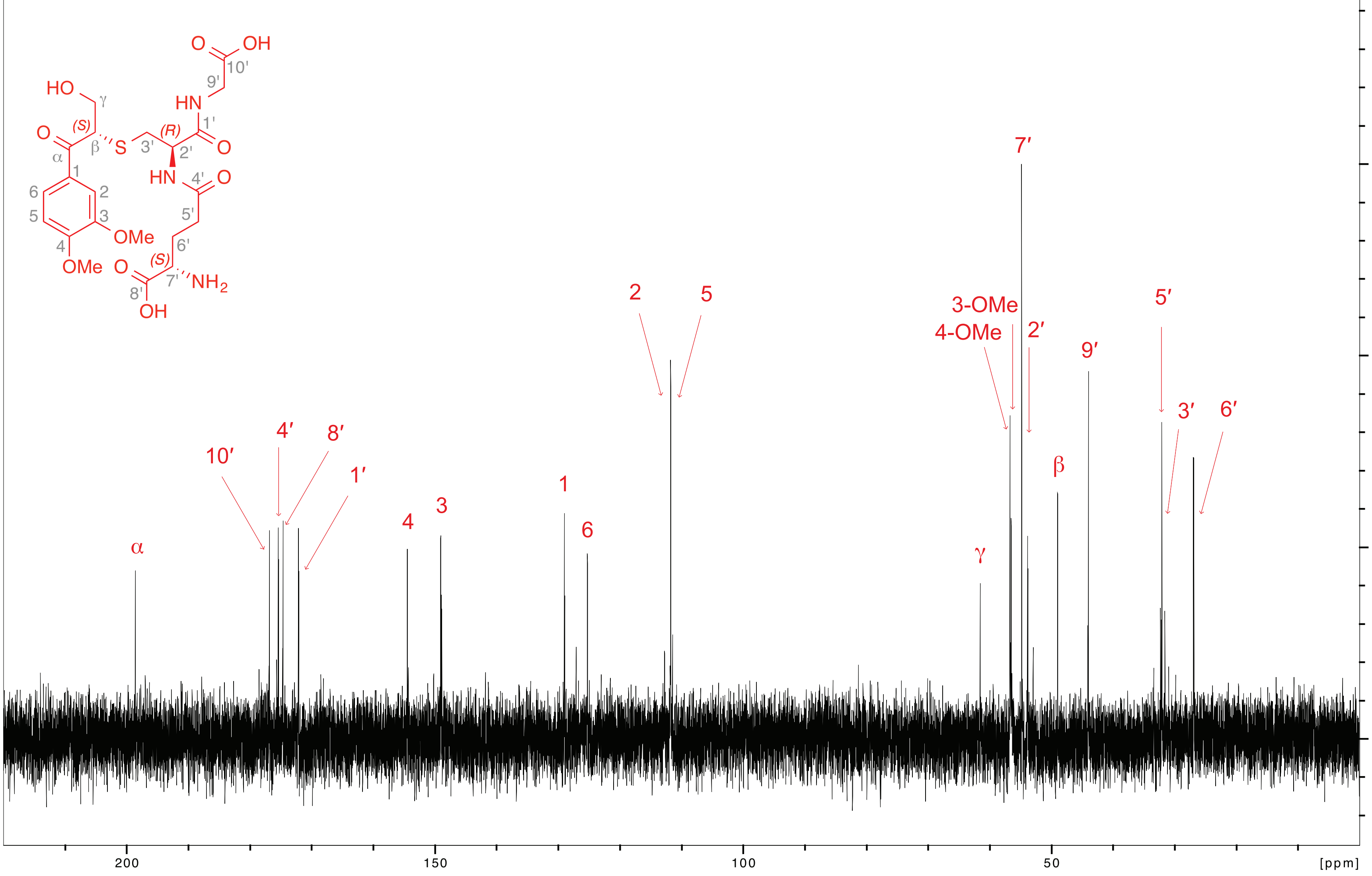

β(*S*)-*S*-glutathionyl-α-veratrylglycerone, GS-β(*S*)VG [from LigP]

^{1}H NMR (700 MHz, D_2O)

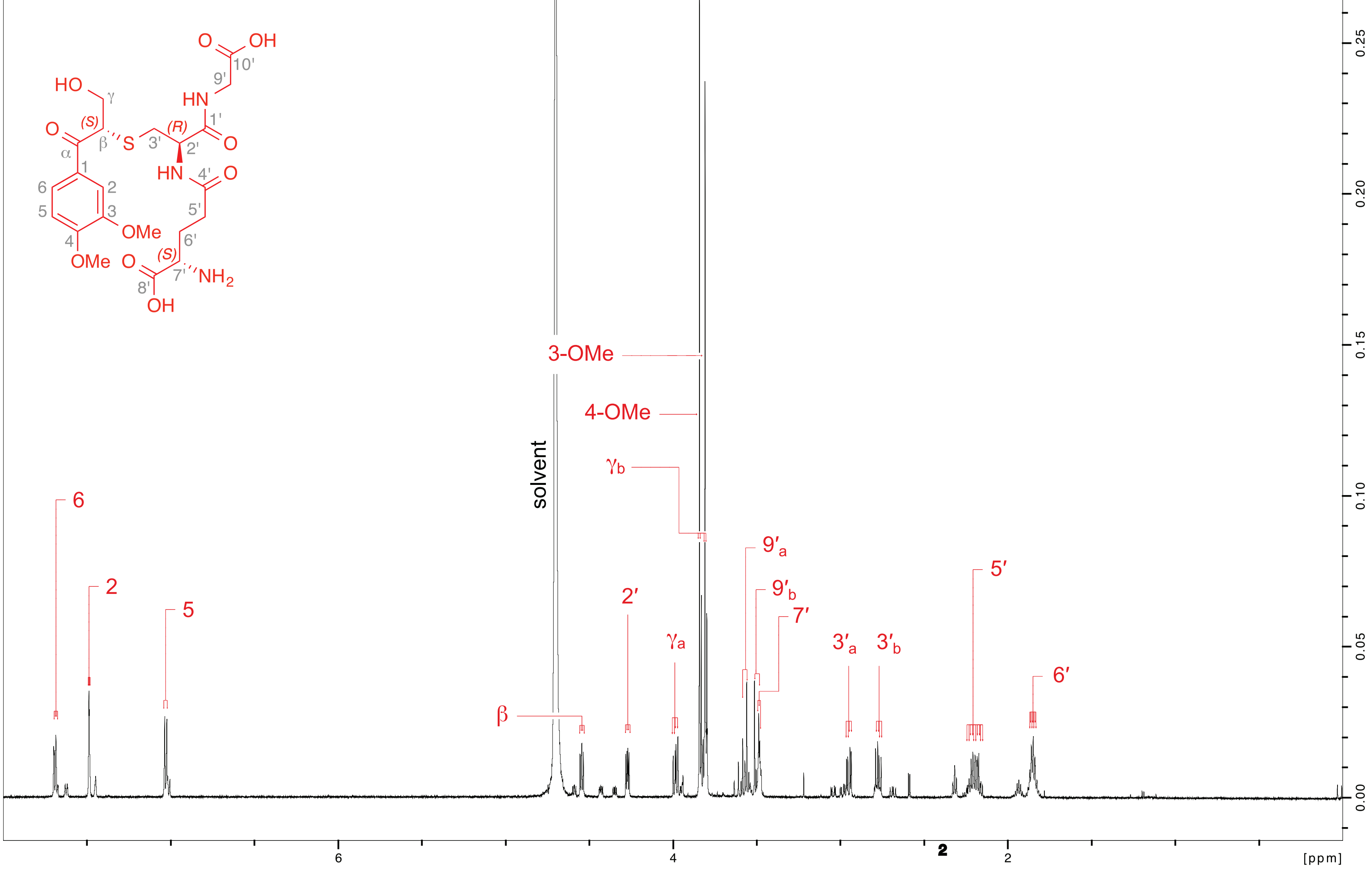

β(*S*)-*S*-glutathionyl-α-veratrylglycerone, GS-β(*S*)VG [from LigP]

^{13}C NMR (176 MHz, D_2O)

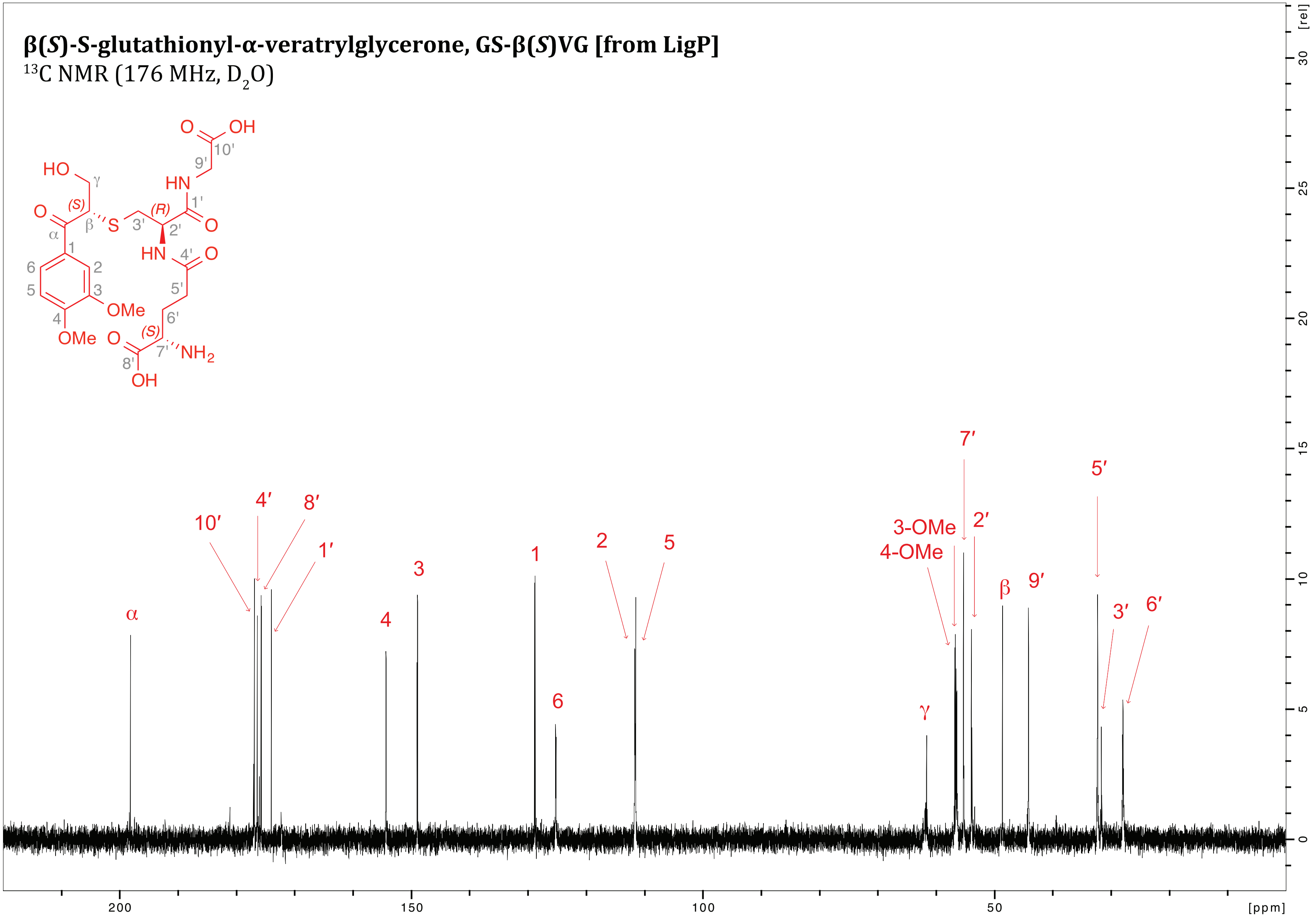

β(*S*)-*S*-glutathionyl-α-veratrylglycerone, GS-β(*S*)VG [not degraded by LigG]

^{1}H NMR (700 MHz, D_2O)

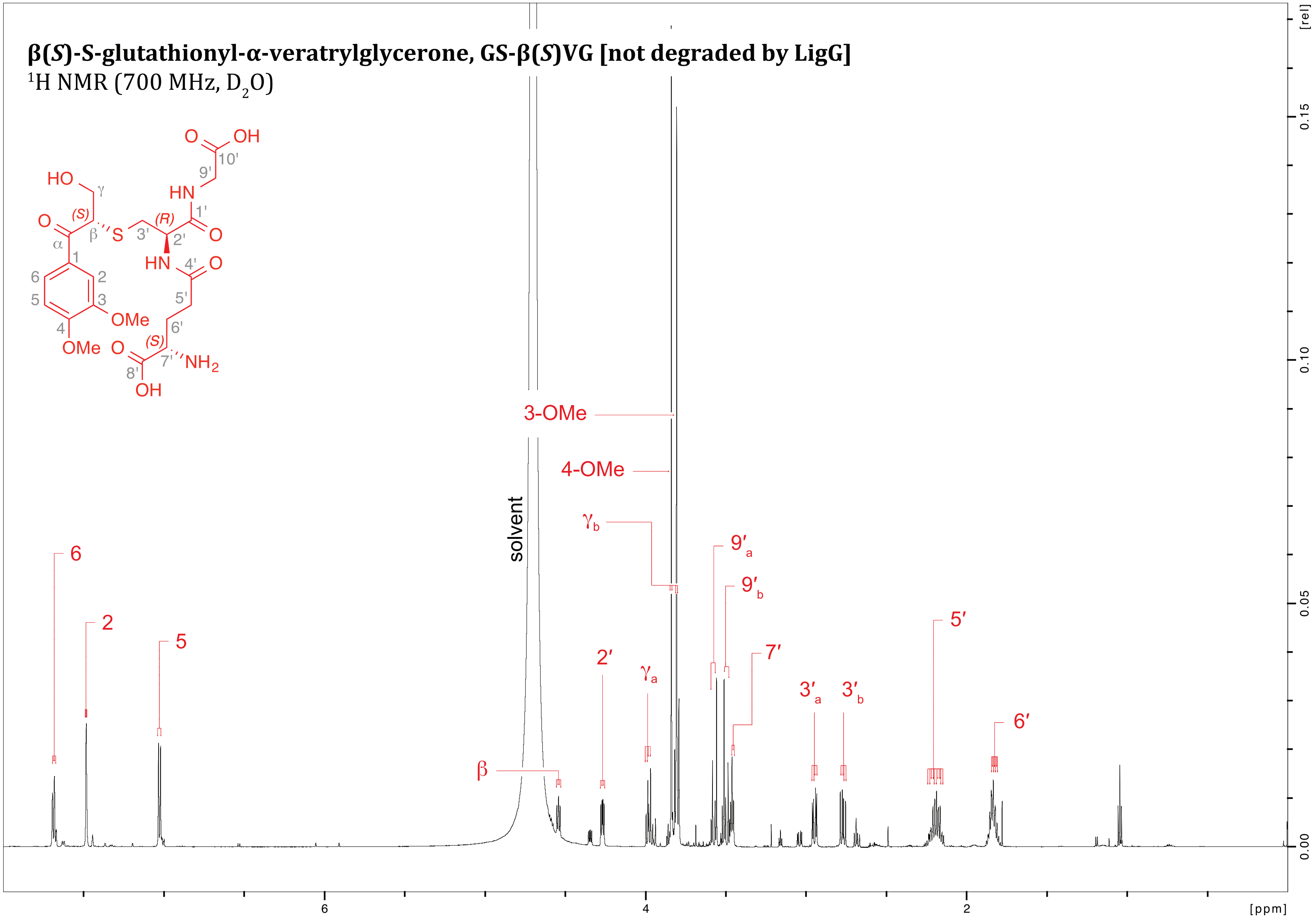

β(*S*)-*S*-glutathionyl-α-veratrylglycerone, GS-β(*S*)VG [not degraded by LigG]

^{13}C NMR (176 MHz, D_2O)

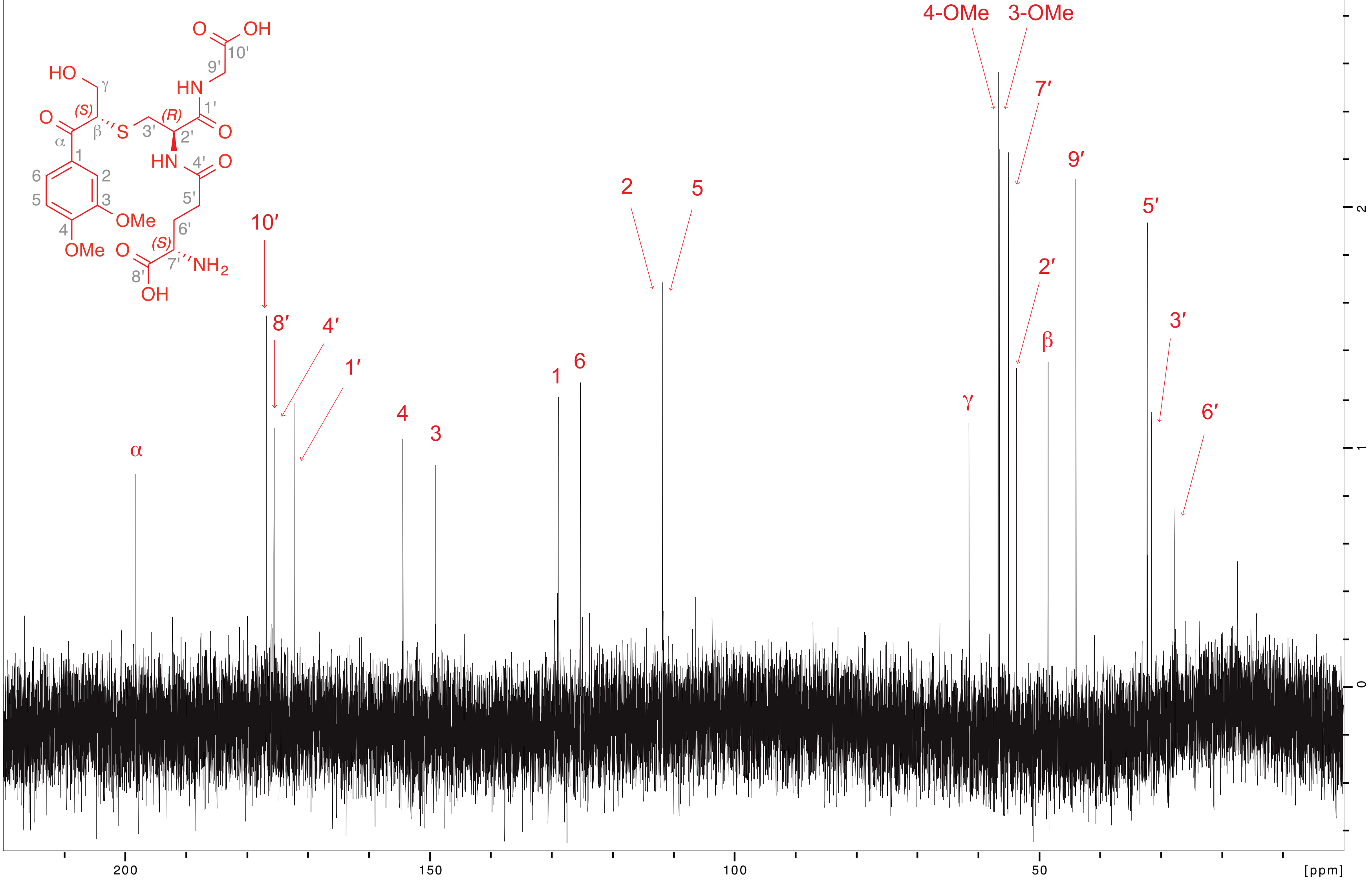

β(*R*)-S-glutathionyl-α-veratrylglycerone, GS-β(*R*)VG [from LigF]

^{1}H NMR (700 MHz, D_2O)

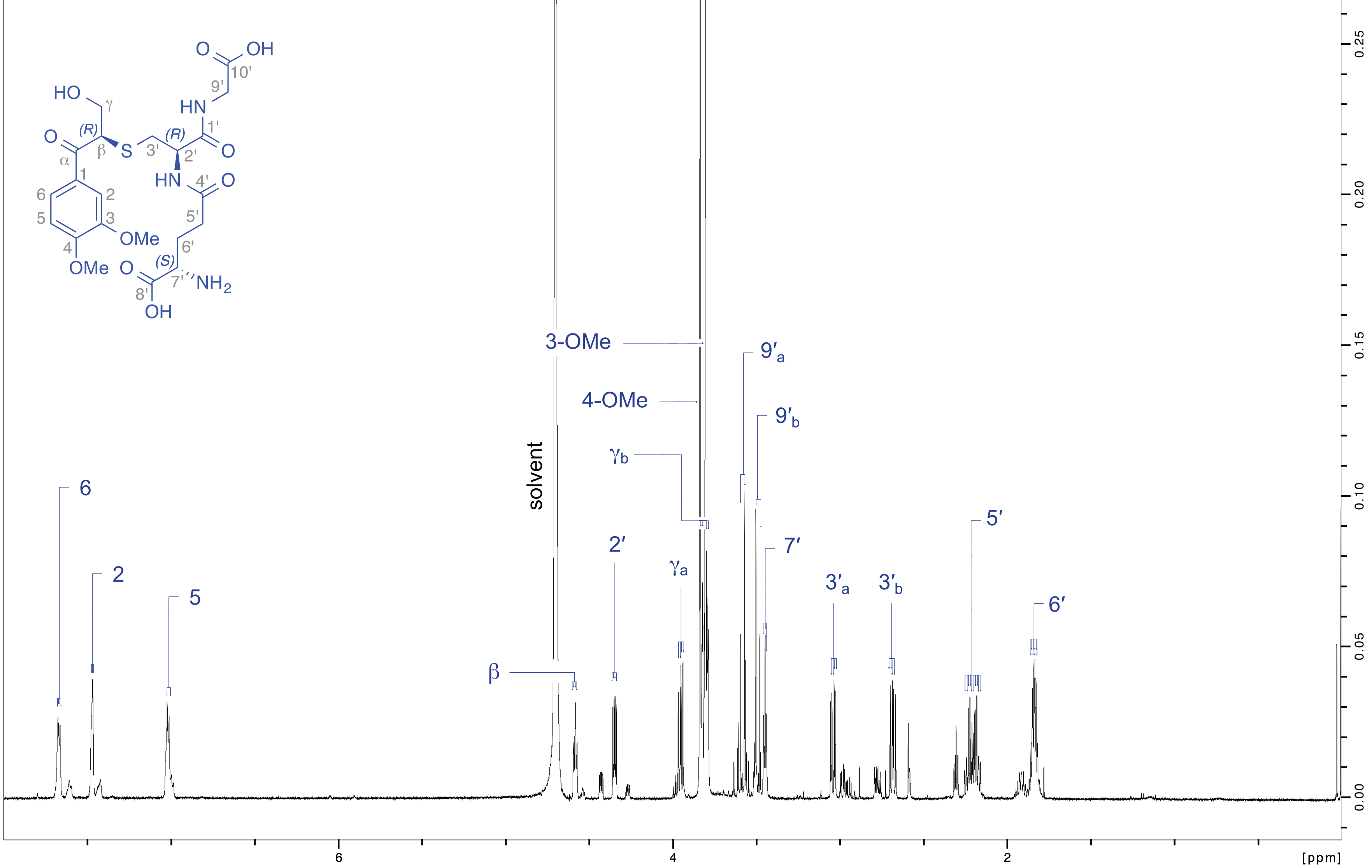

β(*R*)-*S*-glutathionyl-α-veratrylglycerone, GS-β(*R*)VG [from LigF]

^{13}C NMR (176 MHz, D_2O)

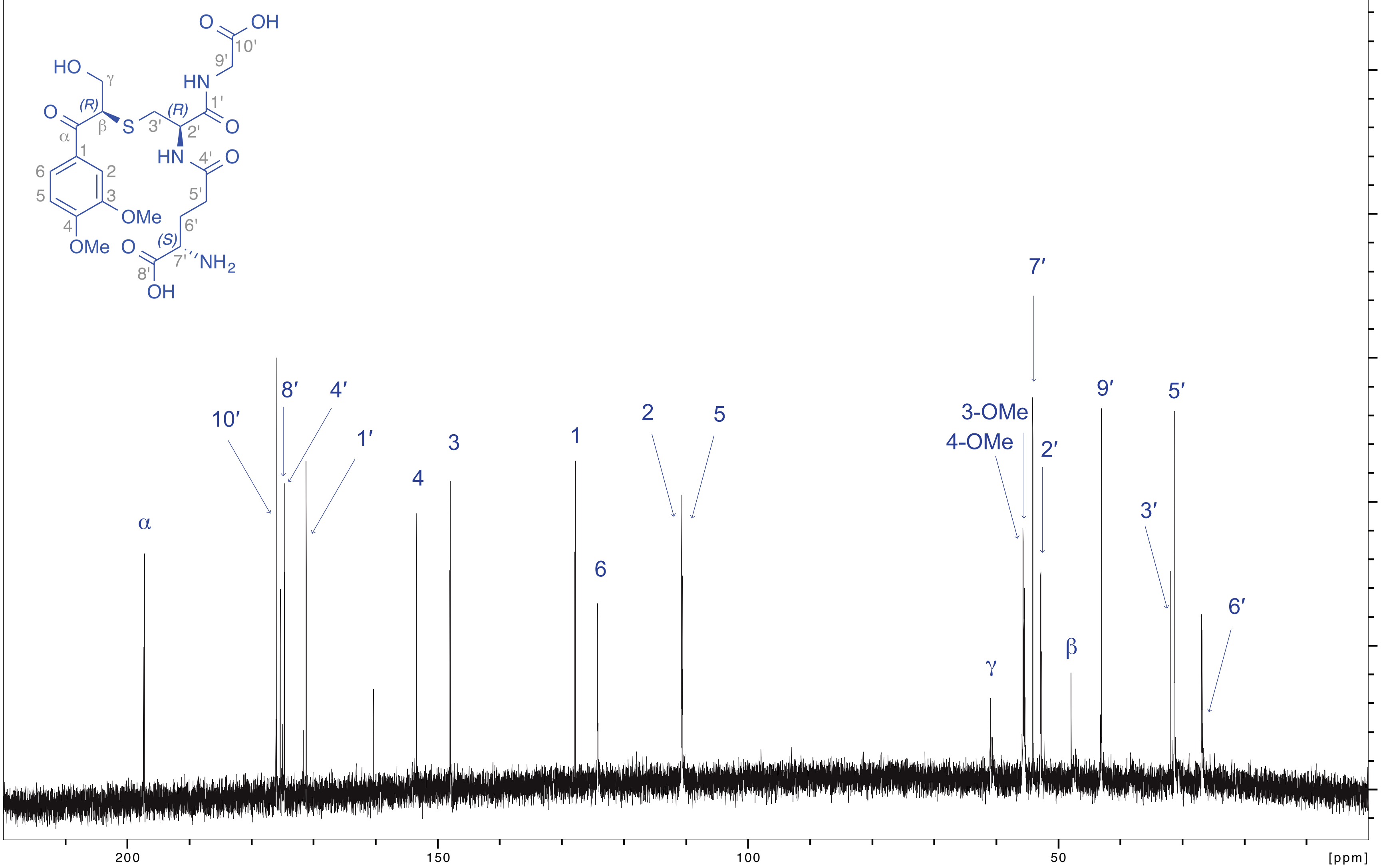

γ,γ-dimethoxy-β(*S*)-S-(methyl N-acetyl-cysteinyl)-α-veratrylpropanone, CS-β(*S*)VP [synthetic]

^{1}H NMR (700 MHz, D_2O)

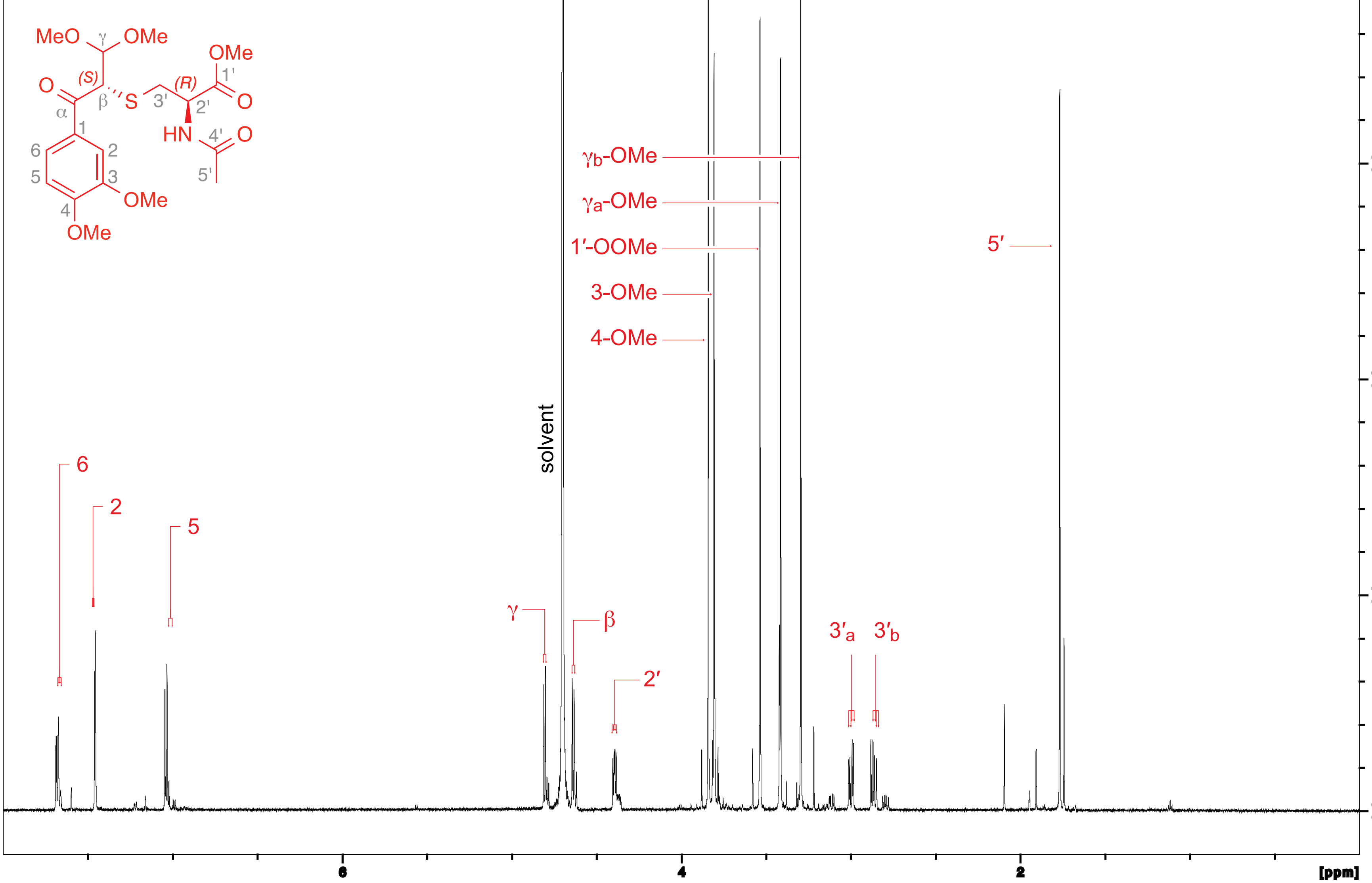

γ,γ-dimethoxy-β(*S*)-S-(methyl N-acetyl-cysteinyl)-α-veratrylpropanone, CS-β(*S*)VP [synthetic]

^{13}C NMR (176 MHz, D_2O)

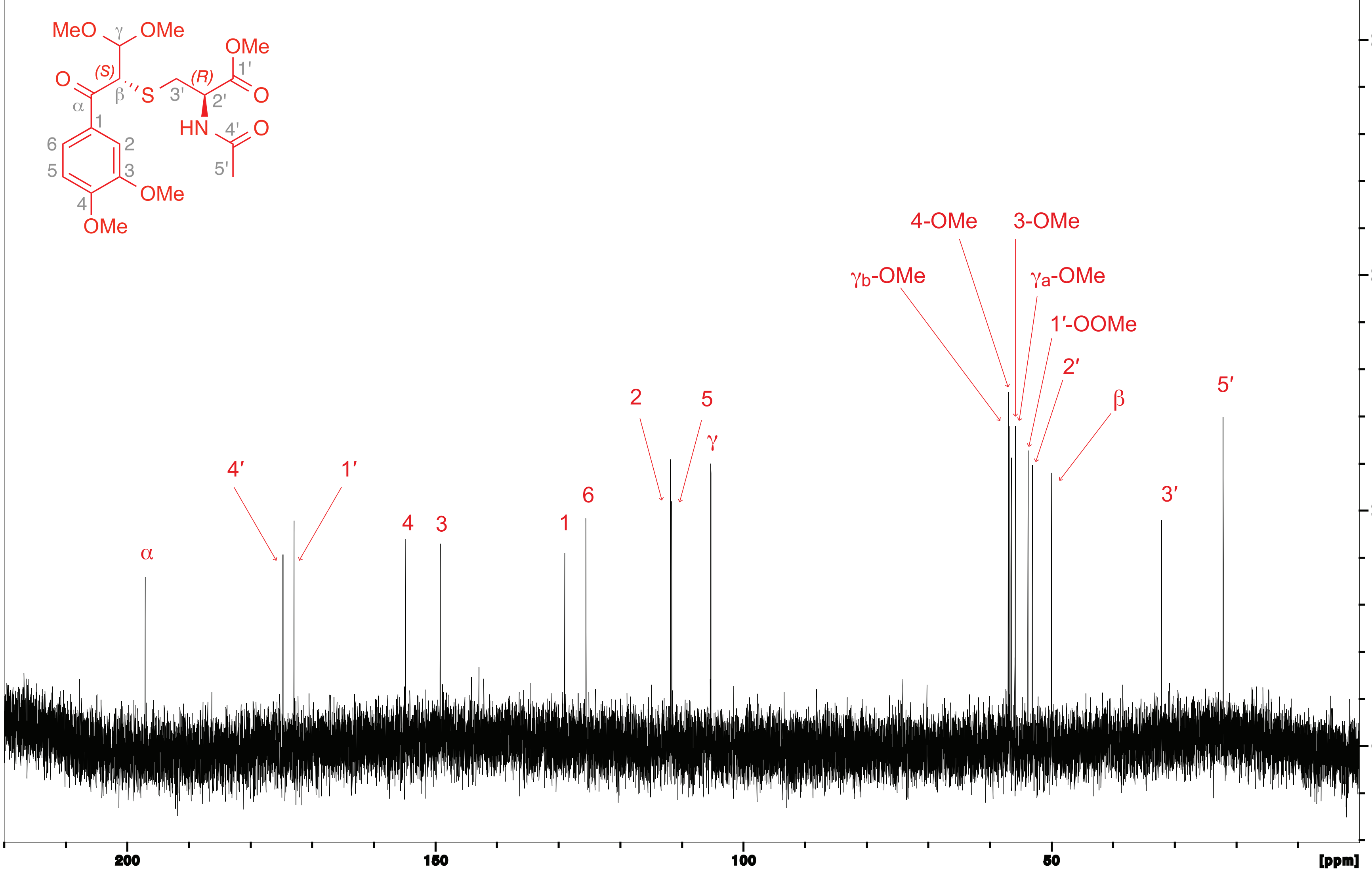

γ,γ-dimethoxy-β(*S*)-S-(methyl N-acetyl-cysteinyl)-α-veratrylpropanone, CS-β(*S*)VP [synthetic]

^{1}H NMR (700 MHz, acetone-d_6)

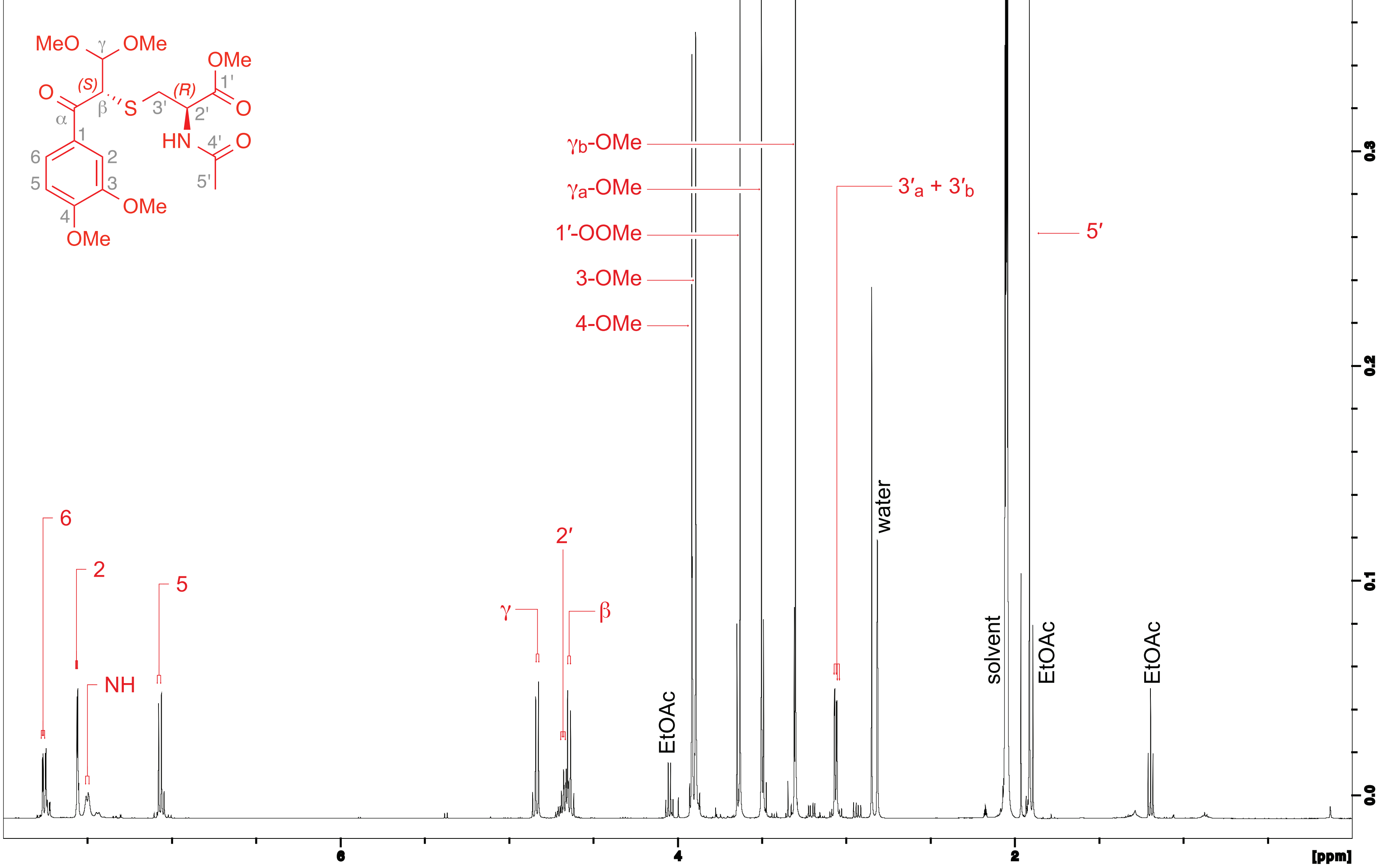

γ,γ-dimethoxy-β(*S*)-S-(methyl N-acetyl-cysteinyl)-α-veratrylpropanone, CS-β(*S*)VP [synthetic]

^{13}C NMR (176 MHz, acetone-d_6)

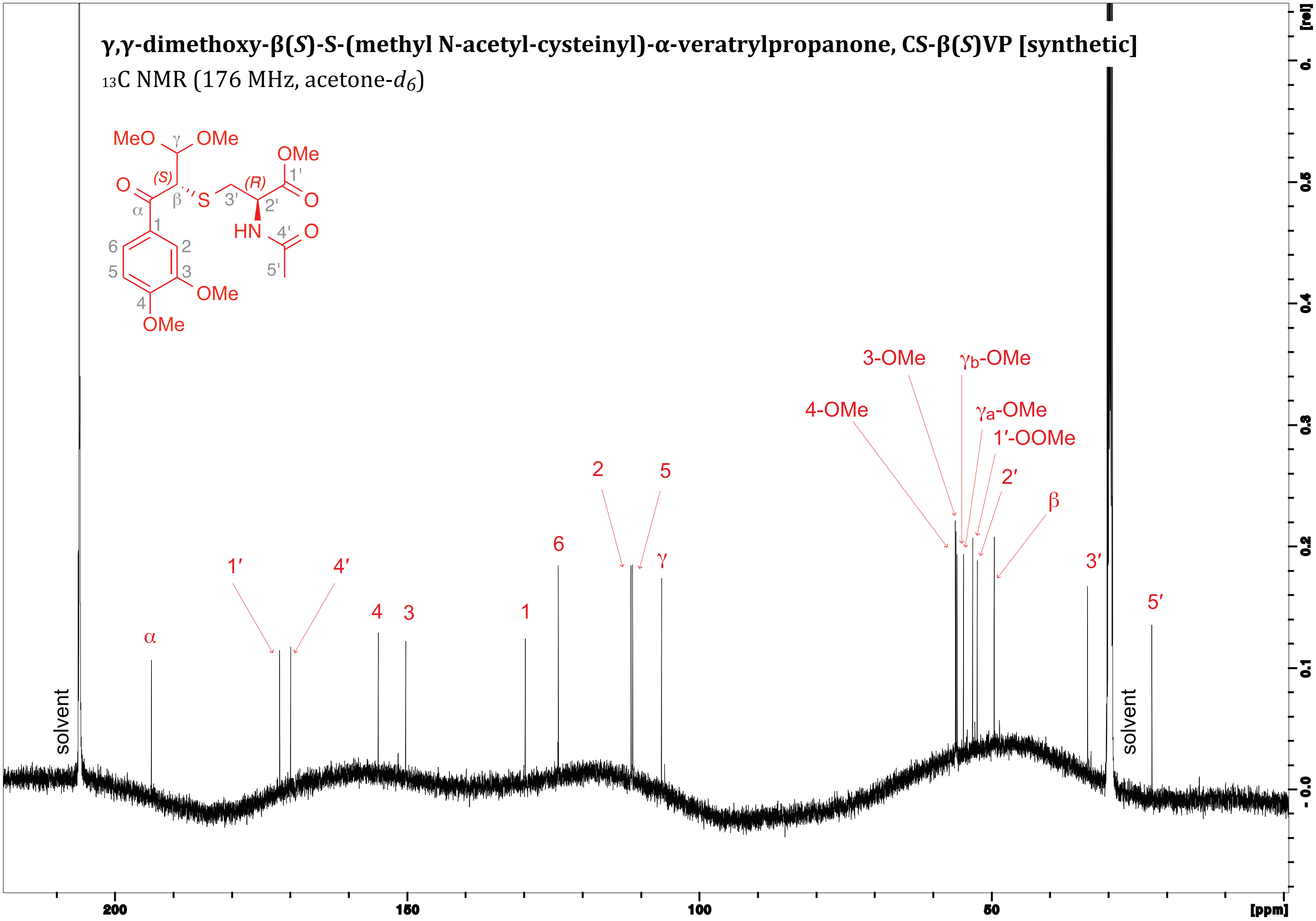

γ,γ-dimethoxy-β(*R*)-*S*-(methyl N-acetyl-cysteinyl)-α-veratrylpropanone, C*S*-β(*R*)VP [synthetic]

$_1$H NMR (700 MHz, D_2O)

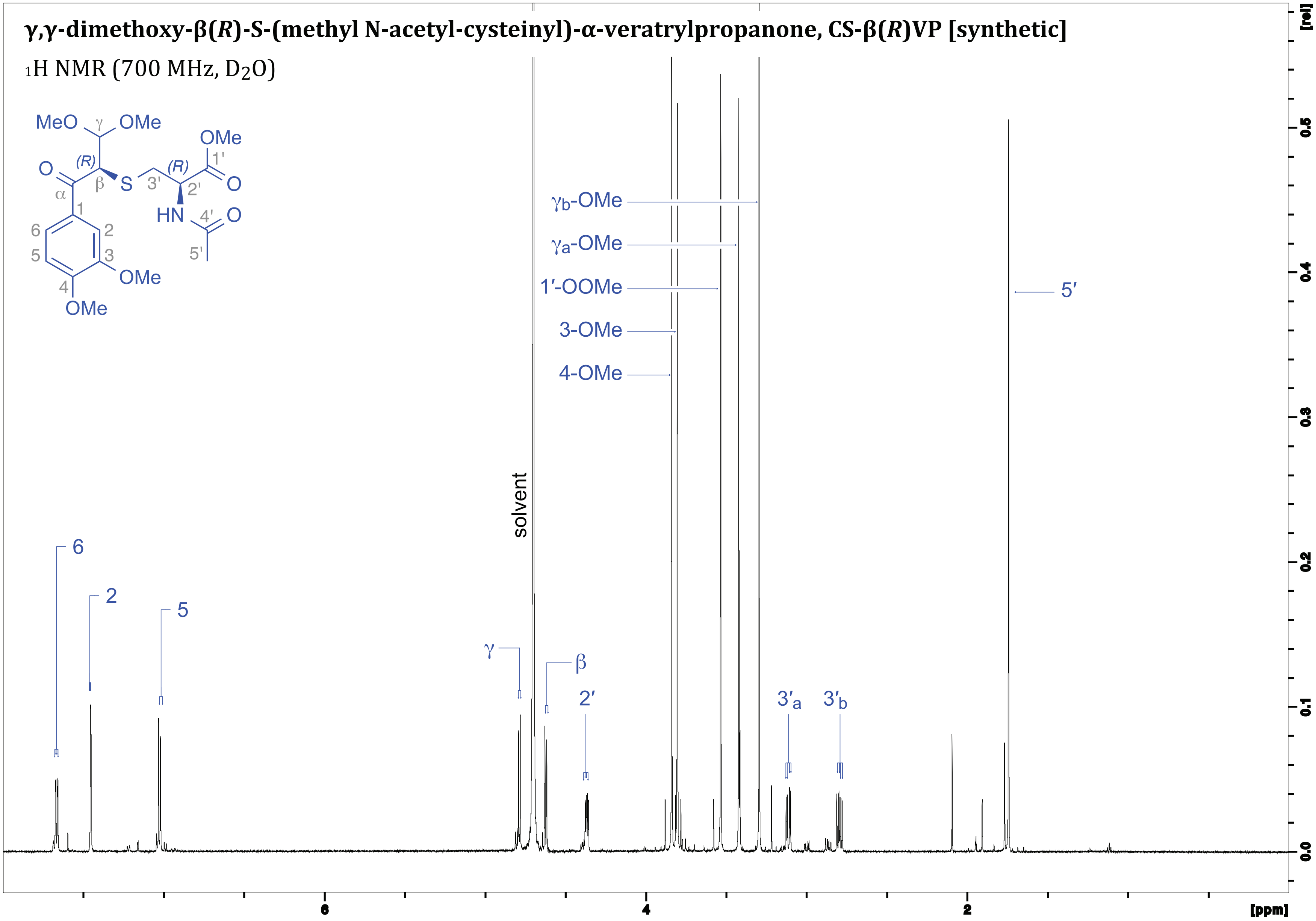

γ,γ-dimethoxy-β(*R*)-S-(methyl N-acetyl-cysteinyl)-α-veratrylpropanone, CS-β(*R*)VP [synthetic]

^{13}C NMR (176 MHz, D_2O)

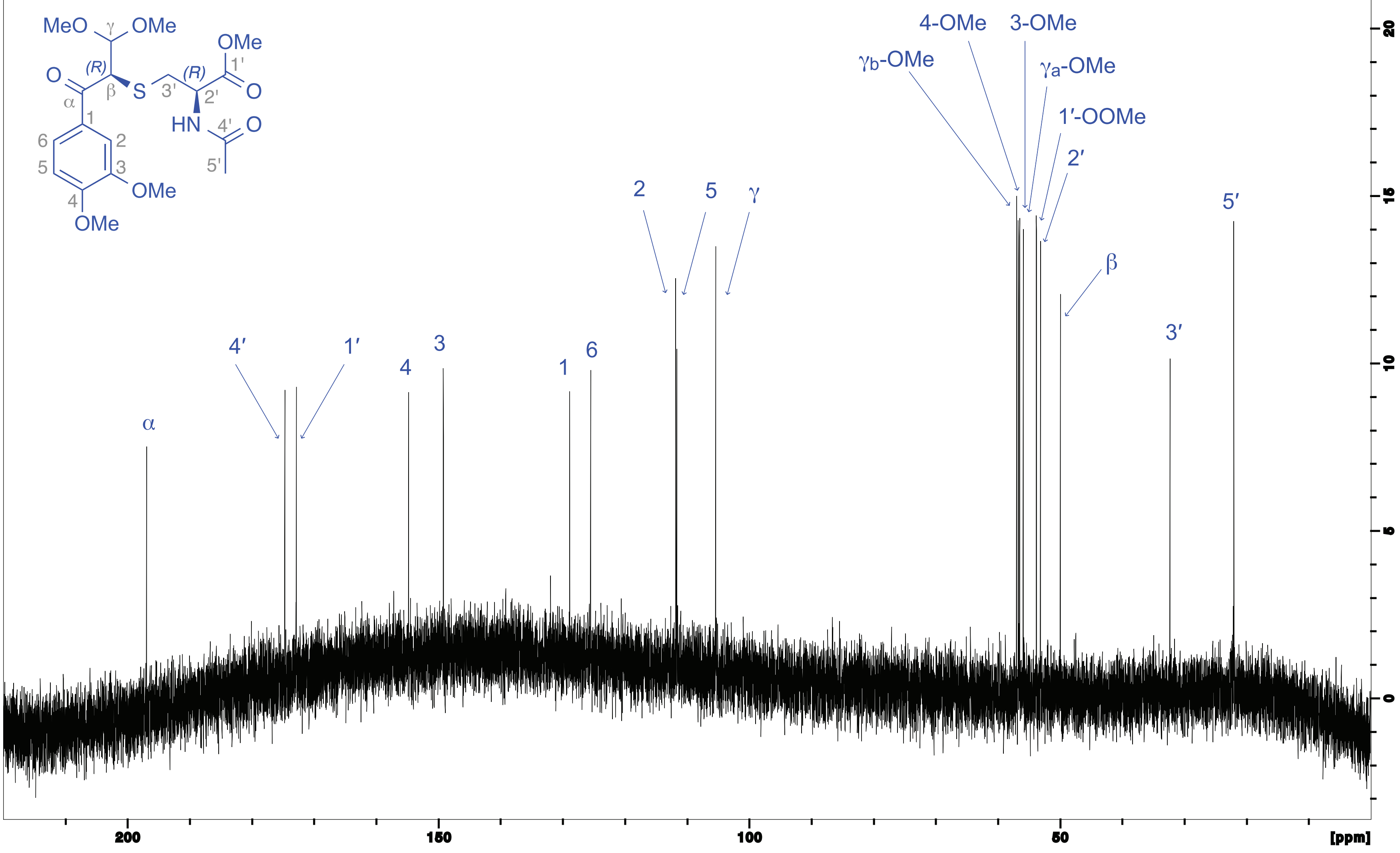

γ,γ-dimethoxy-β(*R*)-*S*-(methyl N-acetyl-cysteinyl)-α-veratrylpropanone, *CS*-β(*R*)VP [synthetic]

^{1}H NMR (700 MHz, acetone-d_6)

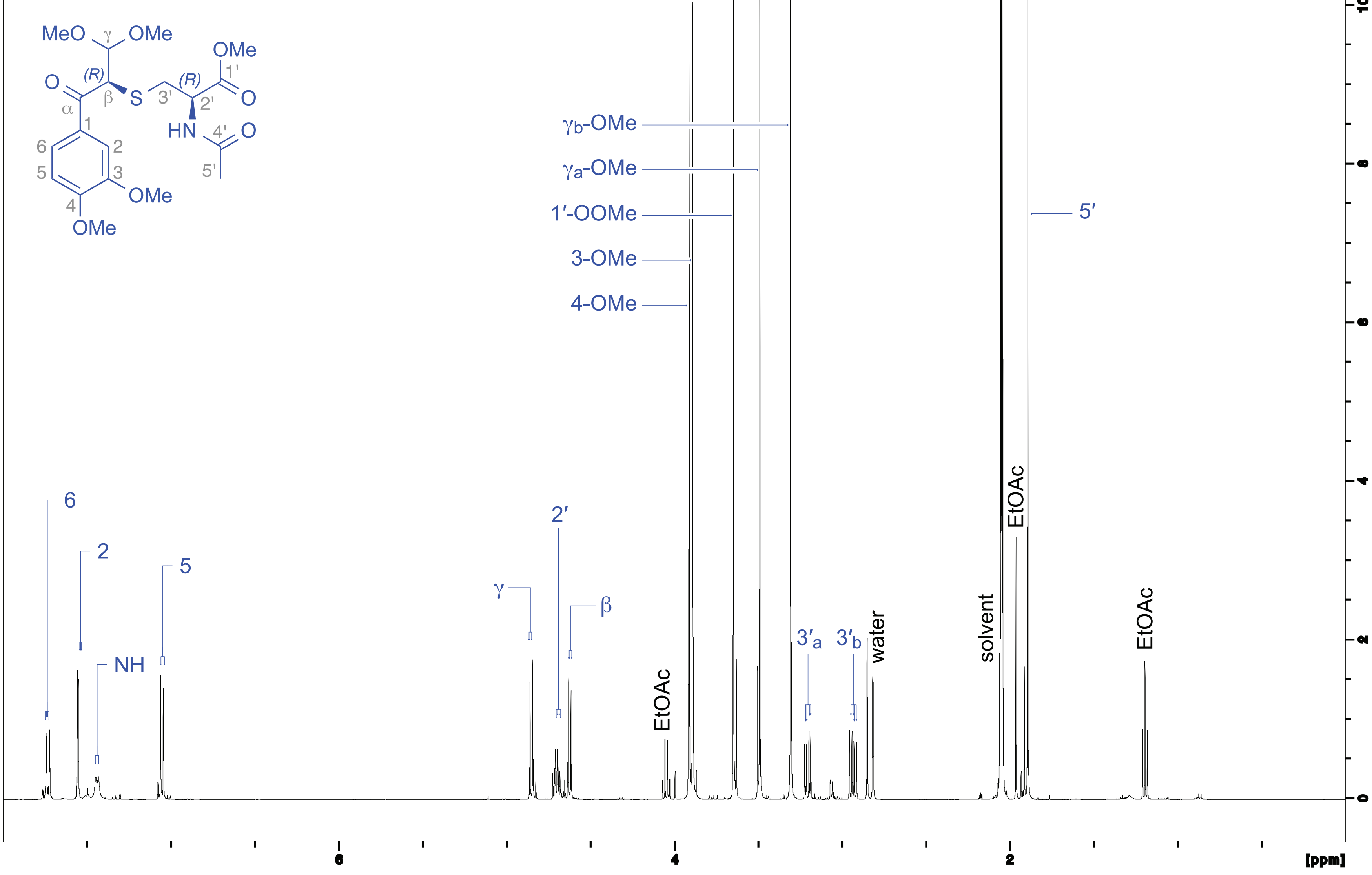

γ,γ-dimethoxy-β(*R*)-S-(methyl N-acetyl-cysteinyl)-α-veratrylpropanone, CS-β(*R*)VP [synthetic]

^{13}C NMR (176 MHz, acetone-d_6)

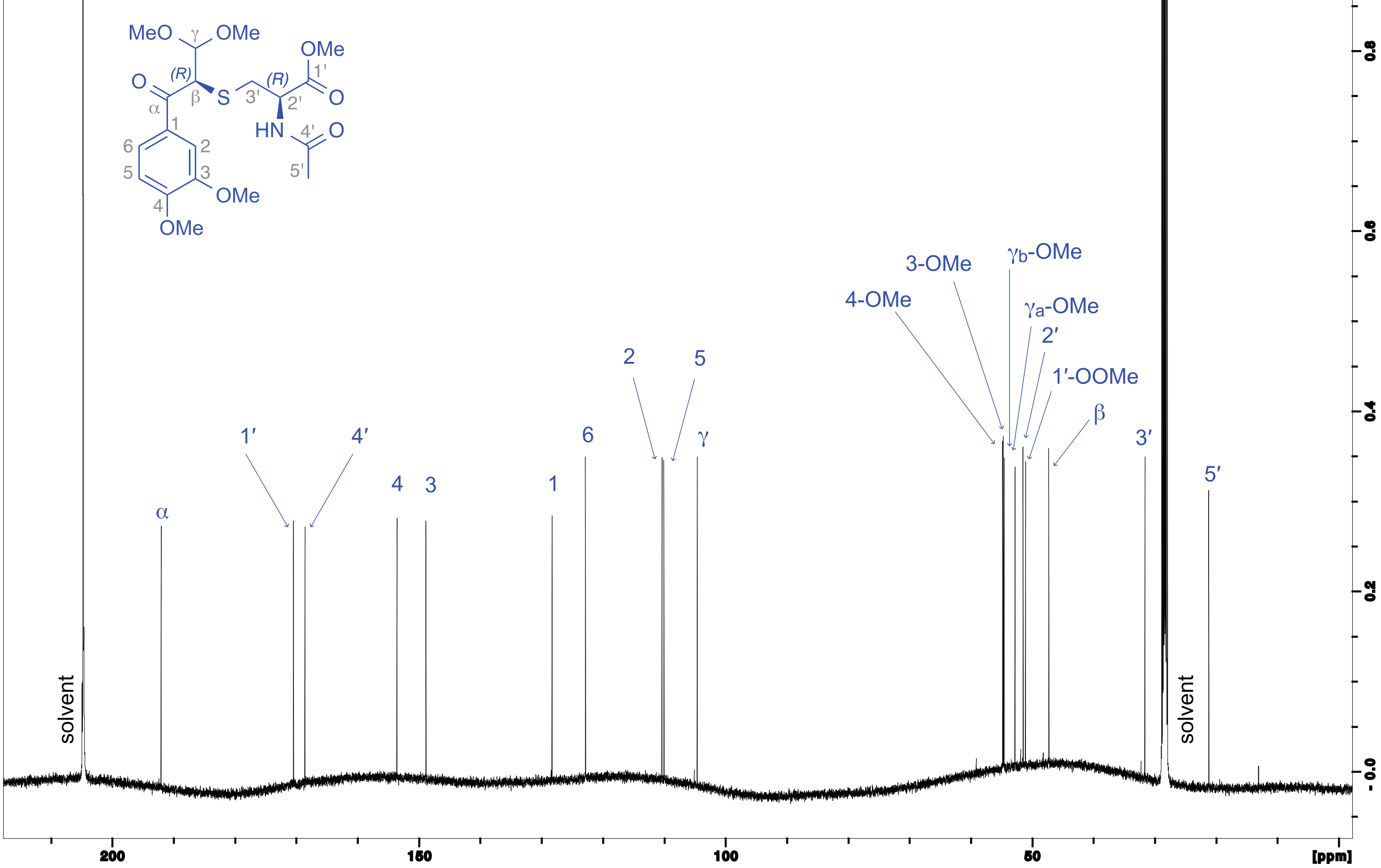

Supplement: Supplemental Data [file supp_M113.536250_jbc.M113.536250-1.pdf]
